# Proteomic and metabolomic responses of priority bacterial pathogens to subinhibitory concentration of antibiotics

**DOI:** 10.1038/s44259-025-00147-7

**Published:** 2025-09-16

**Authors:** Monika Subanovic, Dean Frawley, Ciara Tierney, Trinidad Velasco-Torrijos, Fiona Walsh

**Affiliations:** 1 Department of Biology, The Kathleen Lonsdale Human Health Institute, Maynooth University, Maynooth, Ireland; 2 Department of Chemistry, The Kathleen Lonsdale Human Health Institute, Maynooth University, Maynooth, Ireland

**Keywords:** Computational biology and bioinformatics, Microbiology, Molecular biology, Systems biology

## Abstract

This study employed a comprehensive proteomic and metabolomic analysis to characterize adaptive cellular mechanisms of priority pathogens—*Escherichia coli, Klebsiella pneumoniae, Enterococcus faecium*, and *Staphylococcus aureus*—under sub-inhibitory concentrations of antibiotics. Despite significant metabolomic perturbations, some pathogens had minimal or no significant changes in their proteome. Notably, trimethylamine metabolism was consistently altered across all species, suggesting its role in survival under antibiotic stress. Shared adaptive responses to chloramphenicol in *S. aureus* and *E. faecium* are related to translation, oxidative stress management, protein folding and stability, biofilm formation capacity, glycine metabolism and osmoprotection. Alterations in quaternary amines and trimethylamine metabolism suggest alternative nitrogen and carbon utilization pathways in response to antibiotic stress. In *S. aureus*, vancomycin suppressed metabolism, including D-alanine metabolism, and global regulators LytR, CodY and CcpA. These findings offer insights into early antimicrobial resistance mechanisms and highlight critical proteins and metabolites linked to antibiotic tolerance.

## Introduction

Antibiotics have been important in saving lives and enabling advances in modern medicine^1^. However, the rapid emergence of antibiotic resistance in bacteria poses a critical threat to public health. The World Health Organization (WHO) reported that antibiotic-resistant bacteria cause approximately 700,000 deaths annually, with projections exceeding 10 million deaths by 2050^2,3^. Nosocomial infections, prevalent in up to 50% of hospitalized patients, are especially concerning due to high antibiotic usage contributing to emergence and spread of multidrug-resistant (MDR) bacteria and untreatable infections^4,5^. In 2017, the WHO identified 12 bacterial families as 'priority pathogens', including Enterobacteriaceae like *Klebsiella pneumoniae* and *Escherichia coli* resistant to carbapenems or third generation cephalosporins^6,7^. Resistance genes such as *bla*_*KPC*_ and *bla*_*NDM-1*_ exacerbate treatment challenges, often necessitating alternative antibiotics, which can further drive multi drug resistance (MDR) evolution^8,9^. Similarly*, E. coli* is implicated in diverse clinical infections and include strains resistant to major antibiotic classes, with resistance genes like extended-spectrum beta-lactamases (ESBLs) and carbapenemases contributing to global health concerns^10,11^.

The Gram-positive pathogens *Staphylococcus aureus* and *Enterococcus faecium* also pose significant threats to human health. *Staphylococcus aureus*, part of the human microbiota, causes infections like osteomyelitis and infective endocarditis. The most important pathogen methicillin-resistant *S. aureus* (MRSA) is usually treated with vancomycin; however, vancomycin-resistant *S. aureus* (VRSA) has emerged^12–14^. Additionally, strains resistant to other antibiotics like chloramphenicol and oxacillin are prevalent. Chloramphenicol resistance is mediated by the *cfr* gene^15^, while the *mecA* gene contributes to oxacillin resistance^14^. The emergence of resistance to multiple classes of antibiotics in *S. aureus* highlights the pressing need for further research into the systematic changes that occur in this species in response to antibiotic stress. *E. faecium*, associated with hospital outbreaks, has shown rising rates of vancomycin-resistant Enterococci (VRE) in both the EU and the USA, with infection rates exceeding 14.9% and 30%, respectively^16,17^. Although combination therapies like chloramphenicol and daptomycin show promise in treating VRE infections^18^, resistance to chloramphenicol in *E. faecium* underscores the need to understand bacterial responses to maximize drug efficacy^19^.

Metabolomics and proteomics are valuable for analysing bacterial responses to environmental stress, including antibiotic treatment. Changes in upstream protein cascades influence downstream metabolomic profiles, directly affecting phenotypes^20,21^. Proteomics helps in identification of genes and proteins required for antibiotic resistance, while metabolomics reveals alterations in metabolic pathways that influence drug susceptibility and resistance development^22,23^. Combined, these technologies offer insights into the mechanisms driving resistance and susceptibility.

This study aimed to define the proteomic and metabolomic profiles of antibiotic-susceptible *E. coli, K. pneumoniae, E. faecium*, and *S. aureus* under sub-inhibitory antibiotic stress. Sub-inhibitory concentrations of antibiotics are present in an environment and may be encountered at the site of infection in treated patients^24^. These low concentrations of antibiotics could have a biological effect on environmental or clinical strains including such that leads to development of resistant strains^24^. However, these systemic changes in susceptible strains have not been sufficiently explored yet. Gram-negative species were treated with cefotaxime, ciprofloxacin, kanamycin, and imipenem, while Gram-positive species were exposed to chloramphenicol, vancomycin or oxacillin. The effect of these antibiotics was studied due to emergence of strains resistant to chosen antibiotics while chloramphenicol is predicted to be re-introduced as a last resort antibiotic to treat infections caused by drug-resistant strains. By integrating these -omics approaches, we aimed to comprehensively characterize poorly understood initial cascades of bacterial responses to these important antibiotics.

## Results

### Effect of sub-MIC of antibiotics on proteomes of *E. coli* MG1655, *K. pneumoniae* NCTC418*, E. faecium* NCTC13169, and *S. aureus* NCTC8325

To determine the sub-inhibitory concentrations for both proteomic and metabolomic studies, the MICs of these antibiotics were identified. For *K. pneumoniae*, MIC of cefotaxime, ciprofloxacin, kanamycin, and imipenem equalled 0.0625, 0.008, 8 and 0.25 *μ*g/ml, respectively. For *E. coli*, MIC of same antibiotics equalled 0.0312, 0.008, 16 and 0.5 μg/ml, respectively. For *E. faecium* and *S. aureus*, MIC of chloramphenicol was 8 *μ*g/ml. For *S. aureus*, MIC of oxacillin and vancomycin were 0.125 and 1 *μ*g/ml, respectively.

The total number of detected proteins at the pre-filtering stage was consistent across biological replicates, averaging 1337, 1472, 649 and 797 proteins across all treatment groups in *E. coli* MG1655, *K. pneumoniae* NCTC418, *E. faecium* NCTC13169 and *S. aureus* NCTC8325, respectively (Supplementary Fig. 1). The intra-group variability was assessed based on median coefficient of variation (CV) being in a range from 12 to 25% across all sample groups, indicating quality replicate reproducibility^25^ (Supplementary Fig. 2).

In MG1655 and NCTC418 experimental groups, PCA revealed weak or no treatment-driven separation of imputed intensities (Supplementary Figs. 3, 4). However, the Pearson correlation coefficient of imputed intensities between these samples of the same or different treatment group was significant and strong (*r* > 0.9, *P* < 0.05) (Supplementary Figs. 3, 4). In NCTC13169 and NCTC8325 experimental groups, except for oxacillin vs. control group, PCA strongly separated antibiotic-treated from control groups (Supplementary Fig. 5). For all those except for oxacillin vs. control group, intra-group Pearson correlation of imputed intensities between samples was high and significant (*r* > 0.88, *P* < 0.05), while inter-group variability was higher compared to intra-group variability (Supplementary Fig. 5). Thus, sub-MIC of tested antibiotics mainly had a weak effect on proteomes of Gram negative (−) species and strong effect on proteomes of Gram positive (+) species.

The total number of proteins that were significantly differentially abundant upon treatment with antibiotics in Gram(-) species was low with a total maximum of 27 differentially abundant proteins (DAPs), while kanamycin- vs. control-treated group for NCTC418 did not have significant DAPs (Table 1). At least 98 proteins were differentially abundant upon treatment with chloramphenicol or vancomycin in Gram(+) species, while oxacillin- vs. control-treated group did not have significant DAPs (Table 2). Summary statistics of peptide counts, raw and imputed LFQ intensities and differential abundance analysis statistics for all experimental groups are presented in Supplementary Data 2. Volcano plots of differentially abundant proteins are presented in Supplementary Figs. 6, 7 for Gram(−) and Gram(+) species, respectively.

**Table 1:** Total number of proteins significantly increased/decreased in abundance and their total number in *Klebsiella pneumoniae* NCTC418 and *Escherichia coli* MG1655 treated with kanamycin, imipenem, cefotaxime or ciprofloxacin relative to the control treatment

	KAN^a^ vs. CT^a^			IMI^a^ vs. CT			CTX^a^ vs. CT			CPFX^a^ vs. CT		
Species	Up^a^	Down^a^	Σ^a^	Up	Down	Σ	Up	Down	Σ	Up	Down	Σ
*K. pneumoniae*	0	0	**0**	2	1	**3**	1	1	**2**	20	7	**27**
*E. coli*	0	1	**1**	11	6	**17**	2	1	**3**	3	0	**3**

^a^*KAN* kanamycin, *CT* control, *IMI* imipenem, *CTX* cefotaxime, *CPFX* ciprofloxacin, *Σ* total number of altered proteins highlighted in bold, *Up* increased in abundance, *Down* decreased in abundance.

**Table 2:** Total number of proteins significantly increased/decreased in abundance and their total number in *Enterococcus faecium* NCTC13169 and *Staphylococcus aureus* NCTC8325 treated with chloramphenicol, vancomycin or oxacillin relative to the control treatment

	CHL^a^ vs. CT^a^			VAN^a^ vs. CT			OXA^a^ vs. CT		
Species	Up^a^	Down^a^	Σ^a^	Up	Down	Σ	Up	Down	Σ
*E. faecium*	117	66	**183**	-	-	**-**	-	-	**-**
*S. aureus*	40	58	**98**	3	267	**270**	0	0	**0**

^a^*CHL* chloramphenicol, *VAN* vancomycin, *OXA* oxacillin, *Σ* total number of altered proteins highlighted in bold, *Up* increased in abundance, *Down* decreased in abundance.

### Effect of sub-MIC of antibiotic on metabolomes of *E. coli* MG1655, *K. pneumoniae* NCTC418, *E. faecium* NCTC13169 and *S. aureus* NCTC8325

**Fig. 1:**
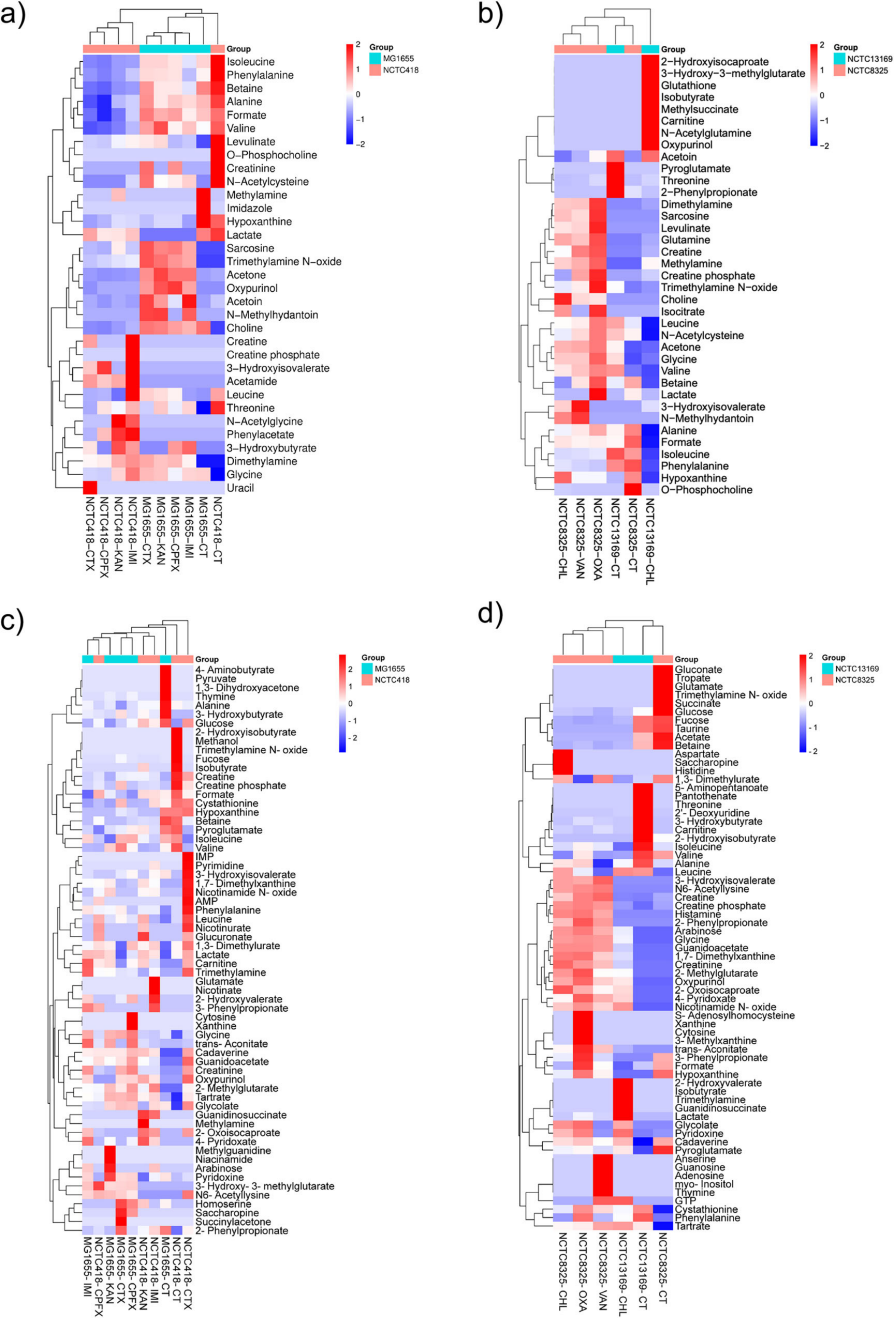
Heatmap profiles of metabolites in studied Gram(-) and Gram (+) species upon treatment with sub-MIC of antibiotics as compared to the control. Intracellular (**a**, **b**) and extracellular (**c**, **d**) metabolomes were detected by ^1^H NMR in at least two biological replicates in at least one group in (**a**, **c**) Gram(-) *E. coli* MG1655 and *K. pneumoniae* NCTC418, and in (**b**, **d**) Gram(+) *E. faecium* NCTC13169 and S. aureus NCTC8325. Blue and red colours represent respective decreased and increased metabolite abundances scaled in the row direction relative to the mean and standard deviation of the metabolite concentrations. KAN kanamycin, CT control, IMI imipenem, CTX cefotaxime, CPFX ciprofloxacin, CHL chloramphenicol, VAN vancomycin, OXA oxacillin.

Untargeted ^1^H NMR metabolomics was performed to compare intracellular and extracellular metabolomes between antibiotic-treated and control groups for select Gram(-) and Gram(+) species. Overlays of ^1^H NMR spectra for each antibiotic treatment group and species are presented in Supplementary Fig. 8, while those for water control treatment groups are presented in published protocol^26^. Inter-group variability was confirmed with PCA score plots, which strongly separated control from antibiotic group in MG1655 (Supplementary Fig. 9), NCTC418 (Supplementary Fig. 10), NCTC13169 and NCTC8325 (Supplementary Fig. 11). The total amount of detected EM was on average 1.8 higher than the number of detected IC metabolites in all tested species irrespective of the treatment (Supplementary Fig. 12, Supplementary Data 3). Of the 33 and 37 distinct IC metabolites identified in Gram(-) and Gram(+) species in at least one experimental group and species, 23 and 21 were common within each group (Supplementary Data 3). A total of 2 and 8 IC metabolites were unique to MG1655 and NCTC418, respectively, while 9 and 7 were specific to NCTC13169 and NCTC8325, respectively (Supplementary Data 3). Similarly, among 64 and 67 EC metabolites identified in Gram(-) and Gram(+) species, 41 and 36 were common within each group (Supplementary Data 3). A total of 10 and 13 EC metabolites were unique to MG1655 and NCTC418, respectively while 8 and 23 were unique to NCTC13169 and NCTC8325, respectively (Supplementary Data 3). Hierarchical clustering of IC metabolite concentrations in the Gram(-) group separated MG1655 and NCTC428, indicating distinct IC metabolome profiles between these species, regardless of treatment (Fig. 1). In MG1655, antibiotic-treated IC profiles were similar but differed from the control, indicating a consistent response to sub-MIC of antibiotics (Fig. 1). In Gram(+), clustering of IC or EC metabolites did not separate NCTC13169 from NCTC8325, though antibiotic-treated NCTC8325 profiles were similar and distinct from their controls (Fig. 1). Overall, IC and EC metabolome profiles differed between antibiotic and control groups and between species exposed to the same treatment. Thus, sub-MIC of all tested antibiotics altered the metabolomes of both Gram(-) and Gram(+) species, in unique ways.

Total numbers of differentially abundant metabolites ranged from 3-14 (IC) and 6-17 (EC) in Gram(-) species (Table 3), and 8-20 (IC) and 18-32 (EC) in Gram(+) species (Table 4). The total number of altered EC metabolites was on average 2.1 and 1.2 times higher than the number of IC metabolites in Gram(+) and Gram(-) species, respectively. Generally, the addition of sub-MICs of the selected antibiotics affected more metabolites in Gram(+) than Gram(-) species.

**Table 3:** Total number of intracellular (IC) or extracellular (EC) metabolites significantly increased or decreased in abundance and their total number in *Klebsiella pneumoniae* NCTC418 and *Escherichia coli* MG1655 treated with kanamycin, imipenem, cefotaxime or ciprofloxacin relative to the control treatment

		KAN^a^ vs. CT^a^			IMI^a^ vs. CT			CTX^a^ vs. CT			CPFX^a^ vs. CT		
Species	CC^a^	Up^a^	Down^a^	Σ^a^	Up	Down	Σ	Up	Down	Σ	Up	Down	Σ
NCTC418	IC	9	1	**9**	13	1	**14**	7	2	**9**	7	1	**8**
	EC	14	3	**17**	7	3	**10**	4	2	**6**	3	8	**11**
MG1655	IC	7	2	**9**	7	2	**9**	2	1	**3**	9	2	**11**
	EC	14	3	**17**	7	1	**8**	8	1	**9**	5	1	**6**

^a^*CC* cellular compartment, *IC* intracellular, *EC* extracellular, *KAN* kanamycin, *CT* control, *IMI* imipenem, *CTX* cefotaxime, *CPFX* ciprofloxacin, *Σ* total number of altered metabolites highlighted in bold, *Up* increased in abundance, *Down* decreased in abundance.

**Table 4:** Total number of intracellular (IC) or extracellular (EC) metabolites significantly increased or decreased in abundance and their total number in *Enterococcus faecium* NCTC13169 and *Staphylococcus aureus* NCTC8325 treated with chloramphenicol, vancomycin or oxacillin relative to the control treatment

		CHL^a^ vs. CT^a^			VAN^a^ vs. CT			OXA^a^ vs. CT		
Species	CC^a^	Up^a^	Down^a^	Σ^a^	Up	Down	Σ	Up	Down	Σ
NCTC13169	IC	11	9	**20**	-	-	**-**	-	-	**-**
	EC	13	5	**18**	-	-	**-**	-	-	**-**
NCTC8325	IC	9	3	**12**	7	2	**9**	7	1	**8**
	EC	16	7	**23**	21	11	**32**	20	11	**31**

^a^*CC* cellular compartment, *IC* intracellular, *EC* extracellular, *CHL* chloramphenicol; *VAN* vancomycin, *OXA* oxacillin, *Σ* total number of altered metabolites highlighted in bold, *Up* increased in abundance, *Down* decreased in abundance.

**Fig. 2:**
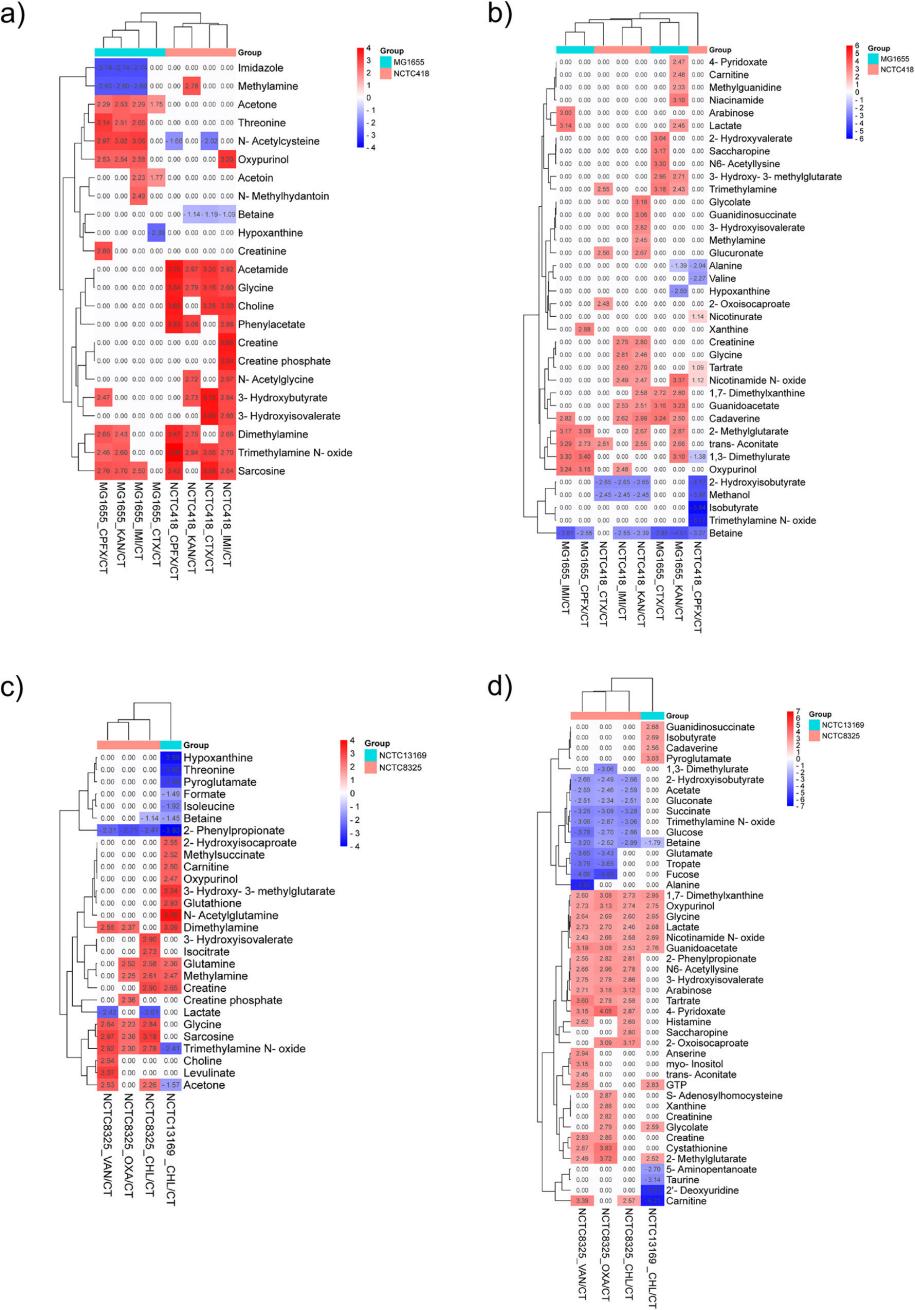
Heatmap profiles of differentially abundant metabolites in studied Gram(-) and Gram (+) species upon treatment with sub-MIC of antibiotics as compared to the control. Intracellular (**a**, **c**) and (**b**, **d**) extracellular significant differentially abundant metabolites in (**a**, **b**) *Klebsiella pneumoniae* NCTC418, *Escherichia coli* MG1655, **c**, **d** *Enterococcus faecium* NCTC13169 and *Staphylococcus aureus* NCTC8325 were hierarchically clustered. The colour scale refers to log_2_ fold change values (FDR < 0.05).

Hierarchically clustered heatmap profiles of significantly altered metabolites revealed generally distinct responses to the same antibiotic between the two tested Gram(-) or (+) species, and variable responses of the same species to different antibiotics (Fig. 2). Several metabolites were linked to general stress response – those that were altered irrespective of the treatment or irrespective of antibiotic’s mode of action. More than half (n = 7, 54%) of the total distinct IC metabolites responsive to imipenem, kanamycin and ciprofloxacin in MG1655 showed consistent alterations across antibiotic treatments including decreased abundance of imidazole and methylamine and increased abundance of acetone, threonine, N-acetylcysteine, oxypurinol and sarcosine. All tested antibiotics showed an increased abundance of acetone in MG1655. In NCTC418, the abundance of IC acetamide, glycine and trimethylamine N-oxide (TMAO) consistently increased with all tested antibiotics.

In NCTC8325, all tested antibiotics increased abundance of IC glycine, sarcosine and TMAO and decreased abundance of 2-phenylpropionate. Alteration of IC 2-phenylpropionate and TMAO abundance was common to both Gram(+) species. Nearly half of distinct EC metabolites (*n* = 19, 48%) were detected across oxacillin, vancomycin and chloramphenicol-treated NCTC8325, suggesting a general stress response. Abundances of seven EC metabolites were altered by all tested antibiotics in both Gram(+) species indicating their involvement in general stress responses common to those particular Gram(+) species.

Metabolites with similar responses to antibiotics sharing modes of action were also observed: more than half (n = 8, 53%) of total distinct IC metabolites responded similarly to cell-wall targeting^27^ imipenem and cefotaxime in NCTC418, which includes decreased abundance of betaine and increased abundance of acetamide, glycine, choline, 3-hydroxybutyrate, 3-hydroxyisovalerate, TMAO and sarcosine; More than half (n = 25, 64%) of total distinct EC metabolites responsive to cell wall-targeting vancomycin and oxacillin^27^ in NCTC8325 had the same response profiles to those antibiotics. Of those, decreased glutamate, tropate, fucose and increased creatine, cystathionine and 2-methylglutarate were specifically responsive to those antibiotics only in NCTC8325. Altered abundance of IC betaine and creatine was specific to chloramphenicol response in both Gram(+) species. Interestingly, to our knowledge, altered abundance of trimethylamine metabolites has not been previously reported as a response to antibiotics in human pathogens.

### Functional annotation of differentially abundant proteins under sub-MIC of antibiotics in *E. coli* MG1655 and *K. pneumoniae* NCTC418

As shown in Table 1, a total number of DAPs under sub-MIC of antibiotics in Gram(-) species was low, with no significant DAPs observed in kanamycin-treated NCTC418. To investigate the functions of DAPs following treatment with sub-MIC antibiotics in Gram-negative species, the UniProt functions of annotated DAPs were analysed (Table 5). Cefotaxime-altered proteins were mainly associated with metabolism and included AMR gene-coded oxygen-insensitive NAD(P)H nitroreductase NfsB (P38489), which can reduce nitroaromatic compounds including antibiotics nitrofurazone and nitrofurantoin^28,29^. Kanamycin altered the abundance of Cca (P06961), involved in translation in MG1655, and did not significantly alter any proteins in NCTC418. Ciprofloxacin mainly altered proteins involved in DNA replication and repair, and SOS response (RecA, UvrA, UvrD). Of those, increased DNA gyrase inhibitor SbmC (A0A2V3K1U3) in NCTC418 is 64% identical to GyrI (P33012) from *E. coli*, which when overexpressed, enhanced resistance against ciprofloxacin^30^. Also, succinate semialdehyde dehydrogenase (Sad), which was increased in NCTC418, is positively associated with heritable resistance to ciprofloxacin in *Pseudomonas aeruginosa*^31^. Imipenem increased the abundance of survival and oxidative stress-associated proteins in MG1655 (metalloprotease LoiP, catalase, iron-sulfur cluster assembly protein) and mainly decreased the abundance of motility-associated proteins. Heat shock proteins were increased in both Gram(-) species in the presence of sub-MIC of imipenem. After manual inspection, we did not find any association between differentially abundant enzymes and metabolites.

**Table 5:** Differentially abundant proteins (*P.adj* < 0.05) upon treatment with sub-MIC of antibiotics in *E. coli* MG1655 and *K. pneumoniae* NCTC418, and their corresponding protein accessions, abbreviated UniProt functions and log_2_ fold changes

Species	Treatment^a^	Protein accession^b,c^	Uniprot protein function^b^	log_2_FC^d^
MG1655	CTX	P0ABJ9	Energy metabolism; oxidative phosphorylation	2.4
		P15639	de novo purine nucleotide synthesis	1.1
		P38489	Reduction of nitroaromatic compounds using NADH or NADPH	-1
	CPFX	P03018	DNA replication and DNA repair	3.4
		P0A698	DNA repair	1.6
		P0A7G6	DNA repair, homologous recombination	1.8
	KAN	P06961	RNA repair, tRNA surveillance and tRNA 3’terminal CCA addition	-3.4
	IMI	P25894	Cleaves substrates preferentially between Phe-Phe residues; membrane permeability; promotes survival	4.5
		P77522	SufBCD complex; assembly or repair of oxygen-labile iron-sulfur clusters under oxidative stress	3.8
		Q7DFV3	N/A	3.3
		P0ABU7	TonB-dependent energy-dependent uptake of receptor-bound substrates	2.4
		P04949	Polymerizes to form the filaments of bacterial flagella	2.2
		P0AD59	Inhibitor of lysozyme	2.2
		P0AES9	Required for optimal acid stress protection. Exhibits a chaperone activity at low pH.	1.7
		P08660	Lysine biosynthesis	1.4
		P21179	Decomposes hydrogen peroxide into water and oxygen	1.4
		P37665	Accessory protein of BamD with OmpA-like domain	1.4
		P36929	Pre-mRNA splicing	1.1
		P0ABZ1	Forms the rotor-mounted switch complex (C ring)	-1.2
		P0ABX8	Controls the rotational direction of flagella during chemotaxis	-1.3
		P75937	Flagellum-dependent swarming motility	-1.3
		P0A6S0	Assembles around the rod to form the L-ring	-1.6
		P77808	Contains domain usually involved in biosynthesis of molybdopterin cofactor	-2.8
		P75960	Removes acetyl groups on target proteins	-3.1
NCTC418	CTX	WP_002890400.1(A0A0H3GNZ4)	Protein export across membrane	-6.6
		WP_002918250.1(B5XSX4)	Translation initiation	1.2
	CPFX	WP_004189779.1(A0A2X3IN34)	N/A	4.3
		WP_004174799.1(W9BE36)	Recombinational DNA repair	4.1
		WP_023284987.1(A0A367NTT1)	N/A	3.8
		WP_004901674.1(N/A)	N/A	3.7
		WP_002911889.1(A0A2V3K1U3)	Inhibits activity of DNA gyrase; Inhibits activity of toxins that target DNA gyrase	3.4
		WP_004899639.1(N/A)	N/A	3.2
		WP_004175424.1(A0A080T2P3)	May be involved in the SOS response (PF07130)	3.2
		WP_004146620.1(W9BQI5)	DNA repair	3.1
		WP_002914769.1(A0A0W8ARI3)	DNA repair; SOS response by activating LexA autocleavage.	2.9
		WP_004146105.1(A0A0S4FW95)	N/A	2.8
		WP_004177783.1(A0A080SRA5)	N/A	2.7
		WP_004216876.1(A0A330VFM9)	N/A	2.6
		WP_002882514.1(W9BK79)	L-asparagine biosynthesis	2.3
		WP_004176765.1(W9BEZ2)	In the presence of manganese, represses expression of *mntH* and *mntS;* Up-regulates expression of *mntP*	2.3
		WP_004148860.1(A0A060VSN7)	Rescue of blocked DNA replication forks via replication fork reversal	2.3
		WP_004222153.1(A0AAP6NZ59)	N/A	2.1
		WP_002883398.1(W9BQA0)	DNA recombination, recombinational repair	1.5
		WP_004223766.1(N/A)	N/A	1.4
		WP_023302796.1(A0A5Q2DPX1)	N/A	1.2
		WP_004151744.1(A0A0W8APX7)	DNA replication, recombination and repair.	1.1
		WP_002906792.1(A0A0W8AUF7)	N/A	-1
		WP_004151134.1(W9BAI4)	Probably phosphorylates lipids; the in vivo substrate is unknown.	-1.1
		WP_004222112.1(A0A483LDB0)	Protein secretion	-1.3
		WP_002901785.1(A0A0W8APA5)	N/A	-1.3
		WP_004224588.1(A0AAP8XMX9)	N/A	-1.3
		WP_004179015.1(A0A0C7F0W9)	L-methionine salvage from methylthioadenosine	-2.5
		WP_004224272.1(A0A486PSC8)	Tryptophan biosynthetic pathway	-2.7
	IMI	WP_002918250.1(A0A0W8AWZ2)	Protein synthesis	1.4
		WP_004146576.1(A0A0W8APP2)	Biosynthesis of succinyl-L-homoserine	-2.3
		WP_004899449.1(A0A9J6RTL0)	Heat shock protein 70 family	3.8

^a^*KAN* kanamycin, *IMI* imipenem, *CTX* cefotaxime, *CPFX* ciprofloxacin

^b^N/A refers to the information not being available

^c^For MG1655, all accessions are UniProt accessions; For NCTC418, UniProt Mapper tool was used to map each RefSeq accession to the Uniprot accession provided in brackets

^d^FC refers to the fold change

### Central biological processes and pathways affected by sub-MIC chloramphenicol in *E. faecium* NCTC13169

**Fig. 3:**
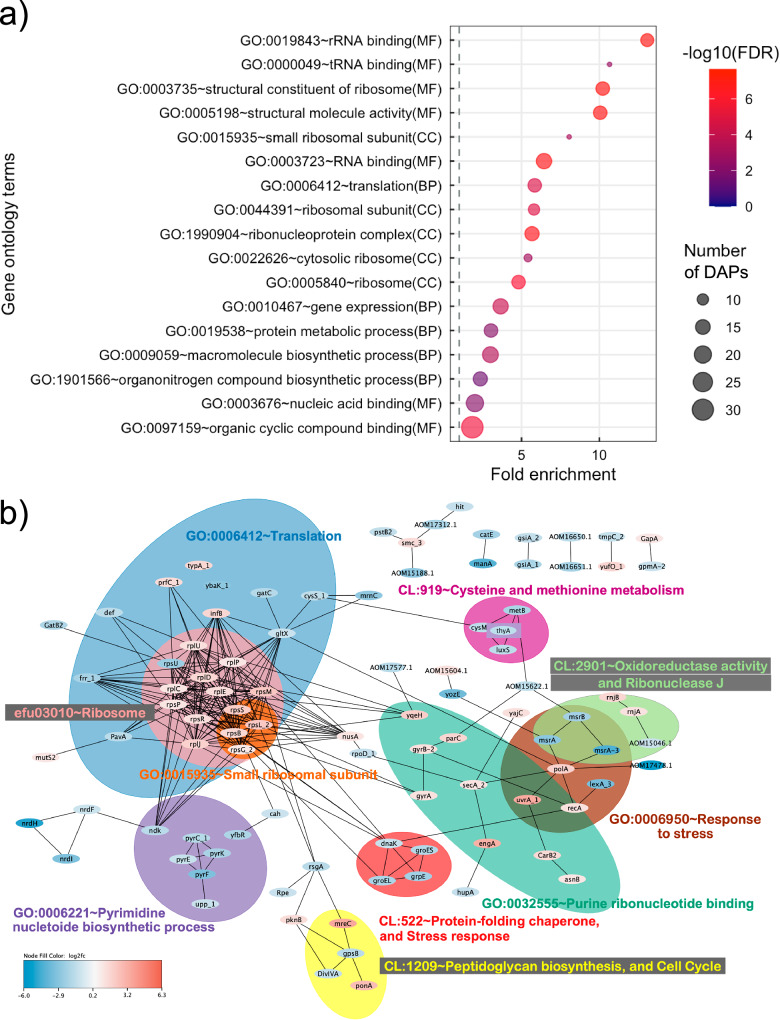
Functional annotation and protein-protein interactions prediction of differentially abundant proteins (DAPs) in *E. faecium* NCTC13169 under sub-MIC of chloramphenicol treatment. Figure (**a**) presents gene ontology (GO) terms significantly enriched in proteins increased in abundance. The coloured scale on the right represents negative logarithmic scale with a base 10 of false discovery rate (FDR) values for each of GO terms. Figure (**b**) presents STRING network of DAPs. Coloured scale on the bottom left refers to logarithmic scale with a base 2 of fold changes for each of these DAPs with blue colour denoting proteins decreased in abundance and red denoting proteins increased in abundance. Coloured ellipses represent sub-networks represented by GO term, KEGG pathway or STRING cluster enriched with DAPs. Abbreviations: MF, molecular function; CC, cell compartment; BP, biological process.

To study NCTC13169 DAPs, all but six RefSeq accessions matched a UniProt accession, which were utilized for enrichment analysis. GO terms and KEGG pathways were not enriched with proteins decreased in abundance (Supplementary Data 4). Based on a fold enrichment and FDR among the GO terms most enriched with proteins increased in abundance were rRNA and tRNA binding, small ribosomal subunit and translation (Fig. 3a, Supplementary Data 4). The protein-protein interaction (PPI) of all DAPs and functional enrichment of the sub-networks were examined (Fig. 3b). The summary of DAPs with their corresponding STRING accessions and functional annotations is in Supplementary Data 5. A total of 80 proteins were singletons without any interacting partners and three of those (ThyA, TypA_1, YbaK_1) were presented in enriched clusters (Fig. 3b, Supplementary Data 5). A central sub-network was enriched in DAPs related to ribosome assembly and/or translation process (Fig. 3b). It interacted with sub-networks related to pyrimidine nucleotide biosynthetic process, purine nucleotide binding, peptidoglycan biosynthesis and cell cycle, protein folding, stress response, oxidoreductase activities, and cysteine and methionine metabolism (Fig. 3b). A sub-network associated with peptidoglycan biosynthesis and cell cycle represents a divisome complex consisting of decreased essential cell division protein DivIVA, cell division regulator GpsB, and increased family penicillin-binding protein PBP1A (also known as PonA) and rod-shaped determining MreC (Fig. 3b). DivIVA is orthologous (65% identical) to DivIVA (V7ZKB8) from *Enterococcus faecalis* involved in cell division, viability, septum closure, polar growth, chromosome segregation, morphogenesis and biofilm formation^32–35^. GTP-hydrolysing ribosome small subunit-dependent GTPase A (RsgA) interacted with the aforementioned sub-network and ribosome/translation-associated sub-network (Fig. 3b). Decreased abundance of RsgA associates with an increased abundance of EC GTP (Fig. 2d). Apart from aforementioned DivIVA, S-ribosylhomocysteine lyase (LuxS) was decreased in abundance (Supplementary Data 5) and its depletion negatively affected biofilm formation in *E. faecalis*^36^.

We explored in more detail the protein repair and oxidative stress-associated proteins altered by chloramphenicol. Four chaperones in the sub-network CL:522 (GroES, GroEL, DnaK, GrpE), three methionine sulfoxide reductases in sub-network CL:2901 (MsrA, MsrA-3, MsrB) (Fig. 3b), protein folding catalyst PPi, and protease Pcp, were decreased in abundance (Supplementary Data 5). Of those, pyroglutamyl peptidase (Pcp) removes pyroglutamate (pGlu) residue from peptides and proteins in bacteria^37^. The IC and EC pGlu were decreased and increased in abundance, respectively (Fig. 2c, d), associating with altered abundance of Pcp.

Two global regulators involved in oxidative stress response were decreased - SpxA and multiple antibiotic resistance regulator (MarR) (Fig. 3b, Supplementary Data 5). SpxA is activated by oxidative or disulphide stress and regulates genes involved in oxidative stress protection^38^. Six DAPs are known SpxA target genes in *B. subtilis* under disulphide stress^39^: transcription elongation factor GreA, 2,5-diketo-D-gluconate reductase A (YvgN), methionine sulfoxide reductases (MsrA, MsrB), carboxypepti—dase (YpwA) and nucleoside diphosphate kinase (Ndk) (Supplementary Data 5). MarR is 99.3% identical to oxidative-sensing regulator enterococcal antibiotic and stress response regulator (AsrR; I6ZX04) involved in resistance to antibiotics^40,41^. The type I glyceraldehyde-3-phosphate dehydrogenase (GapA) and DNA mismatch protein (MutS2) are AsrR target genes^40^, and both are increased in abundance (Fig. 3b). Additional oxidative stress associated proteins were altered by chloramphenicol (Supplementary Data 5): increased pyruvate oxidase SpxB, which catalyses production of hydrogen peroxide^42^, and decreased oxidoreductases including thioredoxin DsbG involved in thiol maintenance and repair^43^. Increased abundance of IC glutathione (Fig. 2c), which protects from oxidative stress^44^, and decreased abundance of oxidative stress inducer hypoxanthine (Fig. 2c)^45^ provide metabolomic evidence of induced oxidative stress. Overall, sub-MIC of chloramphenicol affected management of oxidative stress response and protein repair machinery.

Finally, we explored additional proteomic changes associated with metabolic changes upon chloramphenicol stress. ProV and OpuA are involved in response to high osmolarity and transmembrane transport of osmoprotectants glycine, betaine and proline^46^ (Supplementary Data 5). Their increased abundance associates with decreased abundance of IC and EC betaine (Fig. 2c, d). Decreased abundance of IC phenylalanine (although not statistically significant) associates with increased abundance of phenylalanine/tyrosine decarboxylase PheDC/TyrDC (Fig. 1c, Supplementary Data 5). PheDC/TyrDC uses phenylalanine/tyrosine as a substrate to produce antimicrobial β-phenylethylamine, which is a growth and biofilm formation inhibitor^45,47,48^. Asparagine synthetase (AsnB) catalysing reversible synthesis of asparagine from aspartate and glutamine was increased in abundance (Supplementary Data 5), which associates with an increased abundance of IC glutamine (Fig. 2c). Further, glycine-cleavage system (GCS) protein H (GcvH) involved in glycine oxidation^49^ was decreased (Supplementary Data 5). IC glycine was detected only in chloramphenicol-treated NCTC13169, while EC glycine was increased in the presence of chloramphenicol (Figs. 1c and 2d). Thus, decreased abundance of GcvH associates with altered abundance of IC and EC glycine. Global nitrogen and carbon metabolism regulator, the catabolite control protein A (CcpA) and its regulator phosphocarrier protein (Hpr) were increased and decreased in abundance, respectively (Supplementary Data 5). Their perturbances associate with altered abundances of IC glutamine, isoleucine, and EC lactate (Fig. 2c, d) as these metabolites are part of pathways regulated by CcpA^50^. Trimethylamine metabolism is an alternative carbon and nitrogen-deriving metabolic pathway in bacteria^51,52^. To our knowledge, this study for the first time reported a role for trimethylamine metabolism in the response to chloramphenicol treatment in *E. faecium*. These metabolites are increased EC trimethylamine (TMA, only detected in chloramphenicol-treated group), decreased IC TMA N-oxide (TMAO), and increased IC dimethylamine (DMA) and methylamine (Figs. 1d, 2c, d) in the presence of chloramphenicol.

### Central biological processes and pathways affected by sub-MIC chloramphenicol in *S. aureus* NCTC8325

**Fig. 4:**
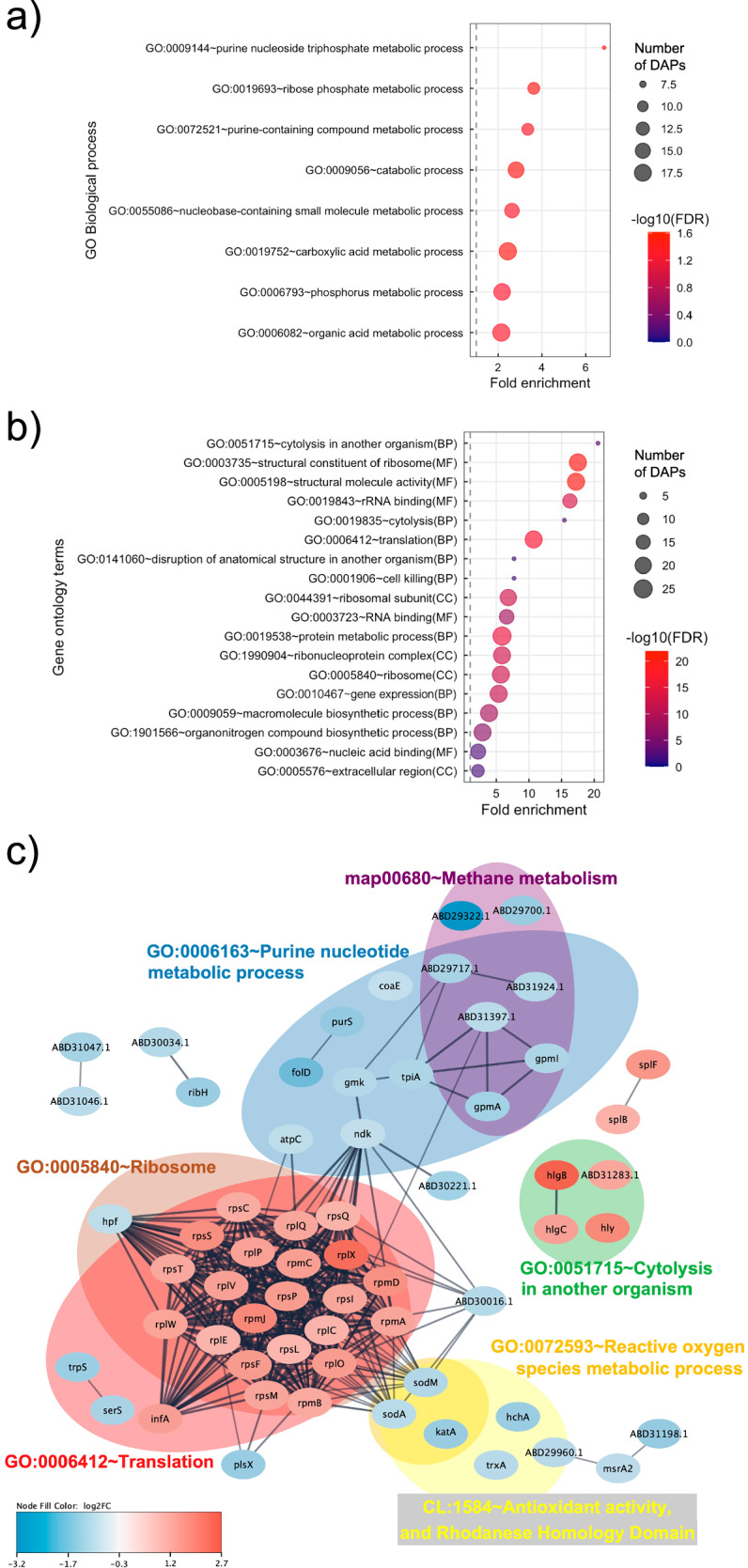
Functional annotation and protein-protein interactions prediction of differentially abundant proteins (DAPs) in *S. aureus* NCTC8325 under sub-MIC of chloramphenicol treatment. **a** presents gene ontology (GO) terms significantly enriched in proteins decreased in abundance and (**b**) proteins increased in abundance. The coloured scale on the right represents negative logarithmic scale with a base 10 of false discovery rate (FDR) values for each of these terms. **c** presents STRING network of DAPs. Coloured scale on the bottom left refers to logarithmic scale with a base 2 of fold changes for each of these DAPs with blue colour denoting proteins decreased in abundance and red denoting proteins increased in abundance. Coloured ellipses represent subnetworks represented by GO term, KEGG pathway or STRING cluster enriched with DAPs. Abbreviations: MF molecular function, CC cell compartment, BP biological process.

GO and KEGG pathway enrichment analysis were performed to study central cellular and metabolic processes affected by sub-MIC of chloramphenicol on *S. aureus* NCTC8325 (Supplementary Data 4). Two KEGG pathways related to ribosome and *S. aureus* infection were enriched with proteins increased in abundance, while three KEGG pathways related to metabolic pathway, methane and carbon metabolism were enriched with proteins decreased in abundance (Supplementary Data 4). The most enriched GO terms for decreased proteins were related to phosphorus, carboxylic acid metabolism, catabolic processes, and purine/ribose phosphate metabolism (Fig. 4a, Supplementary Data 4); The most enriched GO terms for increased proteins were related to cytolysis, protein synthesis, gene expression and organonitrogen compound biosynthetic process (Fig. 4b, Supplementary Data 4). We explored PPI of all DAPs and functional enrichment of the sub-networks (Fig. 4c), and the corresponding summary of associated accessions and functional annotations is in Supplementary Data 5. A total of 45 proteins were singletons without an interacting partner, and 8 of those (CoaE, ABD29322.1, ABD29700.1, ABD31283.1, Hly, KatA, TrxA, HchA) were assigned to enriched clusters (Fig. 4c, Supplementary Data 5). A central sub-network enriched in DAPs was involved in protein synthesis and ribosome assembly (Fig. 4c). Proteins involved in translation were mainly increased (Fig. 4c), while ribosome hibernation promotion factor (HPF) that inhibits translation^53^ was decreased in abundance when treated with chloramphenicol (Fig. 4c). This central sub-network interacted with those associated with purine nucleotide metabolism, methane metabolism, reactive oxygen species (ROS) metabolism and antioxidant activity (Fig. 4c). Decreased putative peptidyl-prolyl cis-trans isomerase (PPIase; ABD30016.1) was a hub protein interacting with all sub-networks except the one related to cytolysis (Fig. 4c). PPiase is 100% identical to PpiB (Q2FIC1)^54^ necessary for optimal folding and activity of secreted virulence factor nuclease (Nuc)^55^. Decreased amount of PpiB negatively affected Nuc activity^54^. Additional protein folding catalyst protein/nucleic acid deglycase (HchA) was decreased in the chloramphenicol-treated group and belongs to a sub-network related to antioxidant activity (Fig. 4c). HchA repairs methylglyoxal-glycated proteins, producing lactate^56^, which aligns with decreased IC lactate abundance (Fig. 2c). An additional eight proteins involved in oxidative stress management were decreased: hydrogen-peroxide removal proteins catalase KatA, superoxide dismutases (SodA and SodM), three thioredoxins, bacilliredoxin and MsrA2 (Fig. 4c, Supplementary Data 5). Bacterial non-heme ferritin (FtnA) and iron-sulfur cluster regulator (IscR) were decreased (Supplementary Data 5), indicating altered iron storage capacity and iron homeostasis management^57,58^.

Abundances of proteins linked to biofilm formation, cell wall synthesis and cell growth were altered by chloramphenicol. Decreased and increased abundance of respective LuxS and immunodominant staphylococcal antigen A (IsaA) (Supplementary Data 5) indicate alteration of the biofilm signalling pathway^59–62^. Decreased abundance of lipid II isoglutaminyl synthase (glutamine-hydrolyzing) subunit GatD (Supplementary Data 5), which is a part of the bi-enzymatic complex MurT-GatD essential for synthesis of peptidoglycan in *S. aureus*, is indicative of altered peptidoglycan synthesis. Proteins essential for cell growth were altered (Supplementary Data 5): increased DnaD domain-containing protein essential for DNA replication and DNA repair in *S. aureus*^63^, decreased one cell-division associated protein SepF, a part of the early divisome^64^, and decreased anti-sigma-B factor antagonist (RsbV) that activates a global regulator sigma B factor involved in initiation of RNA transcription^65^. In *S. aureus*, sigma-B regulates a multitude of metabolic, survival and stress-related processes including cell wall synthesis, biofilm formation^66^, antibiotic resistance^67,68^ and virulence factors like α-hemolysin and serine protease SplB^69^. Hence, we observed chloramphenicol-altered proteins associated with altered sigma-B activity and found five proteases, including increased SplB, and virulence proteins and toxins including increased α-hemolysin (Supplementary Data 5). The purine nucleotide metabolic process was enriched with proteins decreased in abundance crucial for survival and growth (Fig. 4c). Of those, nucleoside diphosphate kinase (NDK) was a hub protein (Fig. 4c) previously determined to respond to sub-MIC levels of daptomycin in *S. aureus*^70^.

Additional metabolic changes were associated with proteomic changes. Decreased abundance of GatD and pyridoxal 5’-phosphate synthase subunit (PdxS) associates (Supplementary Data 5) with increased abundance of IC glutamine (Fig. 2c). Both catalyse reversible conversion of glutamine to glutamate. Decreased abundances of acetyl-CoA synthetase and phosphoribosylformylglycinamidine synthase subunit (PurS) associate with decreased abundance of EC acetate (Supplementary Data 5, Fig. 2d). Trimethylamine metabolism was altered with IC TMAO and methylamine being increased in abundance and EC TMAO decreased in abundance (Fig. 2c and d). Altered abundances of IC betaine, creatine, sarcosine and glycine indicate chloramphenicol-impacted glycine, serine and threonine metabolism, while altered IC isocitrate reflects an impact on the TCA cycle (Fig. 2c). FMN oxidoreductase belonging to the NADH:flavin oxidoreductase/NADH oxidase (IPR001155) family was decreased in abundance (Supplementary Data 5). It has a TIM-barrel fold present in trimethylamine dehydrogenase, dimethylamine dehydrogenase, and N,N-dimethylglycine/sarcosine dehydrogenase involved in betaine and sarcosine degradation with formaldehyde and glycine as products^71^. Thus, chloramphenicol-altered abundance of FMN oxidoreductase associates with altered abundances of IC betaine, sarcosine, glycine, methylamine and TMAO.

### Central biological processes and pathways affected by sub-MIC vancomycin in *S. aureus* NCTC8325

**Fig. 5:**
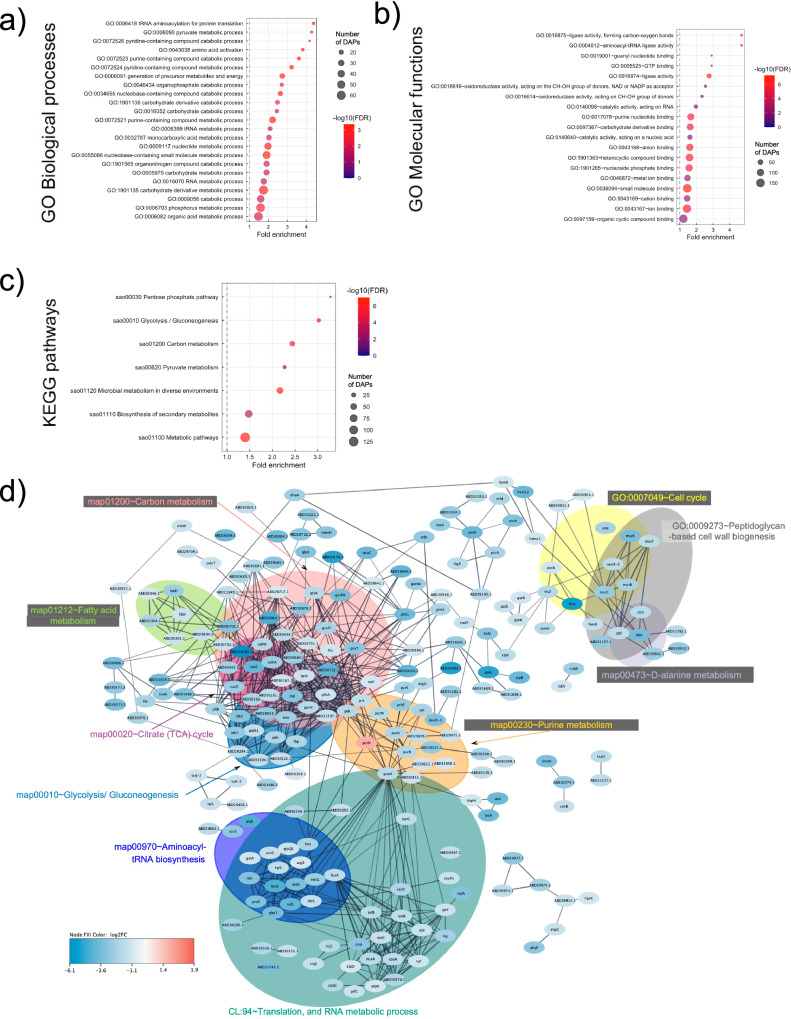
Functional annotation and protein-protein interactions prediction of differentially abundant proteins (DAPs) in *S. aureus* NCTC8325 under sub-MIC of vancomycin treatment. **a**, **b** present gene ontology (GO) terms and (**c**) KEGG pathways significantly enriched in proteins decreased in abundance. The coloured scale on the right represents negative logarithmic scale with a base 10 of false discovery rate (FDR) values for each of these terms. **d** presents STRING network of DAPs. Colored scale on the bottom left refers to logarithmic scale with a base 2 of fold changes for each of these DAPs with blue colour denoting decreased and red increased proteins. Coloured ellipses represent subnetworks represented by GO term, KEGG pathway or STRING cluster enriched with DAPs.

GO and KEGG pathway enrichment analysis were performed to study central cellular and metabolic processes affected by sub-MIC of vancomycin in *S. aureus* NCTC8325. These were enriched only in proteins decreased in abundance (Supplementary Data 4). The enriched GO terms were primary metabolic processes related to organic acid, phosphorus, RNA, nucleotide and carbohydrate derivative metabolic processes including energy-deriving catabolic processes (Fig. 5a, Supplementary Data 4); Similarly, the most enriched KEGG pathways were related to central energy-deriving metabolic processes: pyruvate and carbon metabolism, glycolysis and pentose phosphate pathway; and to secondary metabolism (Fig. 5c, Supplementary Data 4). Most enriched molecular functions were related to ligase, aminoacyl tRNA activity, purine nucleotide and nucleoside phosphate binding and oxidoreductase activity (Fig. 5b, Supplementary Data 4). Thus, sub-MIC of vancomycin supressed both primary and secondary metabolism of NCTC8325. The PPI of all DAPs and functional enrichment of the sub-networks is presented in Fig. 5d, and the corresponding summary of associated accessions and functional annotations is in Supplementary Data 5. A total of 58 DAPs were singletons, and 4 of those (ABD31743.1, ABD30487.1, ABD31936.1, Smc) were placed in enriched clusters (Fig. 5d, Supplementary Data 5). A central network was enriched in proteins decreased in abundance related to primary metabolic processes: carbon metabolism, glycolysis/gluconeogenesis, fatty acid metabolism, citrate cycle, purine metabolism, translation and RNA metabolic process (Fig. 5d). The central network interacted with a sub-network enriched in proteins decreased in abundance involved in cell cycle, peptidoglycan-based cell wall biogenesis and D-alanine metabolism (Fig. 5d). Due to a notable suppression of the primary metabolism, we explored associated global regulators. Transcriptional regulatory protein LytR, global transcriptional regulator CodY and its interacting partner catabolite control protein A (CcpA; ABD30916.1) were decreased in abundance (Fig. 5d, Supplementary Data 5). CodY and CcpA work together to modulate central metabolism, virulence gene expression, and biofilm-associated genes in *S. aureus*^72^, while LytR, upon phosphorylation by LytS, regulates genes involved in autolysis, programmed cell death, biofilm formation and cell wall metabolism^73^. CodY and CcpA connected primary-metabolism associated sub-networks with the one associated with cell cycle and cell wall biogenesis (Fig. 5d). Hence, cellular processes regulated by these transcription factors were explored in more detail. Decreased proteins known to be decreased in abundance in *S. aureus* in *codY* mutant or *lytS* mutant were explored using available sources^73,74^. We found 13.8% (*n* = 12) of 87 *codY*-decreased proteins and 20.3% (*n* = 85) of 418 *lytS-*decreased proteins were decreased in abundance in vancomycin treated *S*. *aureus* in our study (Supplementary Data 5). Out of 12 *codY-*decreased proteins 10 are involved in organic acid metabolism and two in DNA metabolism (Supplementary Data 5). The 85 *lytS-*decreased proteins include CodY and proteins involved in phosphorous metabolic process, translation, proteolysis, generation of precursor metabolites and energy, and metabolism of: porphyrin, DNA, nucleotides, organic acids and carbohydrates (Supplementary Data 5). CodY and CcpA regulate cell wall biosynthesis^72^, hence associated enzymes were explored. Alanine racemase 1 (Alr1) essential for conversion of L-alanine into D-alanine and D-ala-D-ala ligase (Ddl) were decreased in abundance (Fig. 5d), while abundance of EC alanine was decreased (Fig. 2d). IC metabolites increased and decreased by vancomycin treatment involved in type IV lipoteichoic acid (LTA) and TA-peptidoglycan synthesis (sao00552) are choline and O-phosphocoline (detected only in the control group), respectively (Figs. 1b, 2c), indicating metabolic perturbances associated with peptidoglycan synthesis^75^. The metabolite EC GTP modulates the activity of CodY^74^, and was increased in abundance in the vancomycin treated *S. aureus* (Fig. 2d). Purine metabolism (sao00230) enzymes using GTP as a substrate were decreased in abundance: anaerobic ribonucleoside-triphosphate reductase (ABD31936.1) and GTP pyrophosphokinase ((p)ppGpp synthase) (ABD30813.1) (Fig. 5d). Thus, altered abundances of GTP processing enzymes associate with altered abundance of EC GTP. Metabolic perturbances associated with decreased abundances of CodY and CcpA^72^ were observed: decreased abundances of EC glucose and acetate (Fig. 2d), and decreased and increased abundance of respective IC lactate and glycine (Fig. 2c). L-lactate permease (Q2G1N9; Supplementary Data 5) that imports lactate across the membrane was decreased in abundance, including glycolysis/gluconeogenesis (sao00010) enzymes L-lactate dehydrogenase 1 and 2 (Ldh-1 and Ldh-2; Fig. 5d) explaining increased EC lactate and decreased IC lactate abundance (Fig. 2c and d). Also, decreased abundance of putative aldehyde dehydrogenase (ABD31394.1, Fig. 5d) from the glycolysis/gluconeogenesis (sao00010) pathway that catalyzes reversible conversion of acetate, associates with altered abundance of EC acetate (Fig. 2d). Thus, metabolomic and proteomic data provides evidence that NCTC8325 responds to sub-MIC vancomycin by supressing the abundance, and subsequently the activity of master primary metabolism regulators CcpA and CodY, resulting in supressed metabolic and cell growth pathways.

We further investigated any proteomic and metabolomic evidence associated with suppressed metabolism upon vancomycin treatment in *S. aureus*. Increased abundances of EC adenosine (detected only in vancomycin-treated group, Fig. 1d) and GTP (Fig. 2d) and decreased abundance of EC hypoxanthine (although not significant, Fig. 1d) associate with suppression of purine metabolism (Fig. 5d), which was detected in vancomycin-treated *S. aureus*. Decreased abundance of purine nucleoside phosphorylase DeoD-type (PNP) (DeoD2) that produces adenine or hypoxanthine in a reversible reaction (Fig. 5d), associates with altered (although not significant) abundances of EC adenosine and EC and IC hypoxanthine (Fig. 1b, d; Supplementary data 3). Further, succinate-CoA ligase subunit alpha and beta (SucC and SucD) involved in the reversible synthesis of succinate were decreased in abundance, which associates with decreased abundance of EC succinate and suppression of the TCA-cycle (Fig. 5d). Altered abundances of amino acids were examined. Glutamate dehydrogenase (ABD30020.1), catalyzing the reversible reaction of 2-oxoglutarate synthesis from glutamate, and glutamine synthetase (ABD30386.1) catalyzing synthesis of glutamine from glutamate were decreased in abundance (Fig. 5d), which associates with decreased abundance of EC glutamate (Fig. 2d). These enzymes belong to the alanine, glutamate and aspartate metabolic pathway (sao00250). IC and EC glycine were increased in abundance, respectively (Fig. 2c, d). That associates with decreased abundance of enzymes using glycine as a substrate and belong to carbon metabolism pathway (sao01200; the glycine cleavage system proteins: GvcPA, GvcH, GvcT; and serine hydroxymethyltransferase, GlyA) and aminoacyl-tRNA biosynthesis pathway (sao00970; glycine-tRNA ligase, GlyQS) (Fig. 5d). In addition, the valine degradation pathway (sao00280) and associated enzymes were decreased in abundance: branched-chain amino-acid aminotransferase (ABD29684.1) which converts valine to glutamate in a reversible reaction, and downstream enzymes (2-oxoisovalerate dehydrogenase, ABD30691.1; dihydrolipoyl dehydrogenase, ABD30163.1) (Fig. 5d). Also, valine-tRNA ligase (ValS) using valine as a substrate was decreased in abundance (Fig. 5d). Thus, decreased EC valine abundance (although not significant, Fig. 1d) is associated with suppressed valine metabolism. Lastly, IC DMA and TMAO were increased in abundance, and EC TMAO was decreased (Fig. 2c, d). Thus, trimethylamine metabolism plays a role in the response to sub-MIC of vancomycin in *S. aureus* NCTC3825.

## Discussion

The emergence and spread of antibiotic resistance in bacteria are major global threats^2^. As it is a timely and expensive process to develop new antimicrobial agents, more research deciphering molecular mechanisms and key molecules responding to antibiotics is of upmost importance to identify early warning systems for the emergence of AMR pathogens. For this purpose, metabolomics and proteomics can be utilised^76,77^. Untargeted metabolomics, coupled with NMR analysis has proven to be useful for identifying key metabolome alterations in response to antibiotic and environmental stresses^78–81^. Proteomics is a powerful technology that enables comprehensive analysis of bacterial responses to antibiotics and identification of molecular targets for antimicrobial therapies^82–84^. The proteome and metabolome are interconnected, with changes in the upstream proteome often exerting downstream effects on the metabolome^85^. The metabolome is closely related to the biological phenotype^20^ and alterations in metabolic pathways have been implicated in bacterial resistance to antibiotics and stress agents^22,23,78,79^. Thus, in this study, we employed a multi-omics approach to characterise the global proteomic and metabolomic profiles of the priority pathogens *E. coli*, *K. pneumoniae, E. faecium* and *S. aureus* in response to sub-inhibitory concentrations of antibiotics with varied modes of action.

Despite significant metabolomic perturbation, proteomic analysis of *S. aureus* NCTC8325 under sub-MIC oxacillin may not be sensitive enough to detect statistically significant alterations. Similarly, relatively few significantly altered proteins were detected in *E. coli* MG1655 and *K. pneumoniae* NCTC418 upon exposure to sub-MIC of antibiotics. However, longer incubation times and different sub-inhibitory concentration could have resulted in significant alterations. Overall, proteomic changes in Gram(-) species and oxacillin-treated *S. aureus* NCTC8325 under chosen experimental conditions did not indicate major shifts in primary metabolism. Also, observed metabolomic changes did not describe significant shifts in metabolic fluxes. Overall, most metabolomic changes were not directly driven by proteomic changes. Some of the altered metabolites lack a characterized enzyme responsible for their synthesis in the studied species, such as those involved in trimethylamine metabolism. Nevertheless, fluctuations in metabolite abundance do not necessarily correlate with corresponding enzyme levels, as enzyme activity is extensively regulated post-translationally and allosterically through interactions with metabolites^86–88^. This regulation provides an evolutionary advantage, allowing bacteria to rapidly adapt to environmental changes^88^. Additionally, recent discoveries of non-enzymatic metabolism further complicate the integration of proteomic and metabolomic data^89,90^.

Of the significant proteins in Gram(-) species, ciprofloxacin-increased SbmC in NCTC418 is interesting due to its high similarity to antimicrobial resistance protein GyrI from *E. coli*^30^. Hence, SbmC is likely to have a role in reducing the number of lethal double-stranded breaks formed due to ciprofloxacin, which needs further investigation. Further, SOS protein RecA was increased in abundance in both Gram(-) species when treated with ciprofloxacin. A similar finding was already demonstrated suggesting RecA as a promising drug target^91^. Ciprofloxacin-increased abundances of UvrA and UvrD are linked to an increased abundance of RecA^92^. Thus, the SOS response is a primary response to sub-MIC of ciprofloxacin in MG1655 and NCTC418. Cell-wall acting imipenem mainly negatively affected the abundance of motility associated proteins only in MG1655, which associates with a development of functional flagella being dependent on cell wall integrity^93^.

All studied pathogens responded to antibiotics by altering trimethylamine metabolism, which to our knowledge has not been previously reported. Moreover, trimethylamine metabolism has not been well characterised and annotated in any of the investigated bacterial species. Thus, further research should explore its contribution in their survival to antibiotic stress. *Klebsiella pneumoniae* NCTC418 had a consistent increase of IC acetamide, glycine and TMAO abundance upon treatment with all examined antibiotics. Acetamide was recently proven as an antimicrobial molecule due to its effect on DNA ligase involved in DNA replication, but also acts as an antioxidant scavenging free radicals^94^. Similarly, *S. aureus* NCTC8325 responded to all tested antibiotics with increased abundances of IC glycine and TMAO, among other metabolites. Elevated levels of IC glycine and TMAO can influence bacterial survival in both negative and positive ways. Glycine exhibits bactericidal properties, and TMAO can inhibit the electron transport system^95,96^. Conversely, these metabolites also act as osmoprotectants and protein stabilizers, promoting survival under stress^97,98^. However, TMAO has been shown to induce misfolding of slow-folding, proline-rich proteins and causes cell cycle arrest in HeLa cells^99^. Also, TMAO has been proven to reduce susceptibility of wild-type *E. coli* to antibiotics, including kanamycin and ciprofloxicin^100^. Thus, increased abundance of TMAO detected in MG1655 under sub-inhibitory concentration of kanamycin and ciprofloxacin may positively contribute in survival against these antibiotics. Consequently, these metabolites may play a complex and multifaceted role in bacterial survival when exposed to sub-MIC levels of antibiotics. Eight metabolites, of which betaine was decreased and seven other metabolites were increased in abundance, were commonly responsive to imipenem and cefotaxime in NCTC418. Both antibiotics inhibit bacteria by targeting the cell wall synthesis pathway^27^. Of these metabolites, choline is involved in the synthesis of precursors of the peptidoglycan layer, while 3-hydroxybutyrate is a monomer of polyhydroxybutyrate used as a nitrogen and carbon source in times of starvation^101^. Due to its important cell signalling properties and its antioxidant activity against hydroxyl radicals^101^, 3-hydroxybuyrate may play a positive role in the bacterial response to imipenem and cefotaxime. The abundance of IC growth inhibiting-creatinine^102^ increased exclusively in *E. coli* MG1655 in response to the DNA synthesis inhibitor ciprofloxacin. Hence, creatinine may influence DNA replication upon ciprofloxacin exposure.

Chloramphenicol causes impaired ribosome function and translation leading to impaired management of cellular oxidative balance^15,103^. Impaired protein synthesis exacerbates oxidative and disulphide stress, causing the accumulation of non-native disulfide bonds in the cytoplasm^104^. Proteomic and metabolomic evidence from *E. faecium* NCTC13169 and *S. aureus* NCTC8325 highlights overlapping and species-specific responses to sub-MIC of chloramphenicol involving translation, protein folding and repair, oxidative stress, biofilm formation, cell growth and osmoprotection associated cellular machinery. In both species, chloramphenicol disrupted translation and ribosome assembly but triggered adaptive responses. Both species counteracted negative effects on translation by increasing abundance of ribosomal proteins and essential translation initiation factors, while NCTC8325 had decreased abundance of translation-inhibiting ribosome hibernation promotion factor (HPF)^53^. Both species experienced an effect on protein folding catalysts with NCTC13169 to a greater extent. However, NCTC8325 exhibited decreased abundance of a hub chaperone PPIase, potentially impairing enzymatic activity of nuclease (Nuc), which in turn may diminish virulence and biofilm formation capacity^54^. Additional proteomic and metabolomic evidence suggests an effect on biofilm formation capacity under sub-MIC of chloramphenicol. In both species, LuxS was decreased in abundance, which should have a negative and positive effect on biofilm formation in NCTC13169 and NCTC8325, respectively^36,61^. Regarding NCTC13169, increased abundance of PheDC/TyrDC could negatively affect biofilm formation^45,47,48^. Also, increased and decreased abundance of respective glutathione and hypoxanthine may reflect a reduced biofilm formation capacity, as these metabolite trends inhibited biofilm formation in *E. faecalis*^45^. Moreover, increased glutamine abundance associates with the weak biofilm formation phenotype in *E. faecalis*^45^. In NCTC8325, increased abundance of IsaA should positively affect cell wall turnover, virulence, biofilm formation and probably cell separation due to its accumulation in the septal region of dividing staphylococcal cells^59,60,62^. Thus, increased abundance of LuxS and IsaA may positively influence biofilm formation capacity under chloramphenicol stress in NCTC8325.

Cell growth machinery including the divisome was affected by chloramphenicol in both species. Decreased abundance of the early divisome protein SepF in NCTC8325, likely implicated in cell division as previously reviewed^64^, and divisome-associated factors DivIVA and GpsB in NCTC13169, points to reduced cell growth and/or biofilm formation capacity^35^. Regarding NCTC13169, decreased abundance of GTP-hydrolysing ribosome small subunit-dependent GTPase A (RsgA) that couples sub-networks associated with cell division/peptidoglycan synthesis and ribosome/translation may be another key protein in efficient chloramphenicol response. It is bound and inhibited by guanosine pentaphosphate and tetraphosphate ((p)ppGpp), which contributes to the induction of the stringent response. Its deletion in *S. aureus* caused slower growth, decreased ribosome assembly and increased antimicrobial resistance^105^. Thus, an interplay between ribosome biogenesis, RsgA and divisome may play a role in survival under sub-MIC of chloramphenicol. Also, decreased abundance of RNA polymerase sigma factor RpoD essential for bacterial cell viability and proliferation^106^ suggests its major role in survival under chloramphenicol stress. Metabolomic changes are in line with decreased abundance of CcpA indicating its important role in regulating adaption of central metabolism to survive sub-MIC of chloramphenicol. Regarding NCTC8325, decreased abundance of GatD could negatively affect survival and antibiotic resistance under chloramphenicol stress. Its impairment in *S. aureus* negatively affected bacterial growth rate, resistance to β-lactam antibiotics and to lysozyme^107^. The MurT-GatD complex was proposed as a potential new drug target to fight multidrug resistant bacteria^108^. Also, decreased anti-sigma-B factor antagonist (RsbV) may play an important role in chloramphenicol survival. RsbV activates sigma B factor involved in an initiation of RNA transcription^65^. Thus, sigma B activity should be negatively affected, which in turn should negatively impact most cell machinery and metabolic processes including virulence and response to antibiotics^66–69^. A misuse of antibiotics can influence the expression of virulence factors by activating sigma B in drug-resistant *S. aureus* strains, potentially resulting in poorer clinical outcomes^109^. Our study showed chloramphenicol-altered abundance of virulence proteins and toxins associated with Sigma B activity highlighting its potentially critical role in response to sub-MIC of chloramphenicol.

Interestingly, altered glycine metabolism appears to be a shared adaptive mechanism in NCTC13169 and NCTC8325. The major glycine metabolic pathway includes reversible reaction of oxidative cleavage of glycine catalysed by the glycine cleavage system. The products of glycine oxidation catalysed by the glycine cleavage system (GCS) are carbon, nitrogen and energy sources, and methylene-tetrahydrofolate used for the biosynthesis of purine, thymidylate and methionine^49^. Glycine-cleavage system protein H was greatly reduced in chloramphenicol treated NCTC13169, hence maintenance of appropriate quantities of intracellular glycine may be impaired. That could negatively affect purine/pyrimidine synthesis, which is evident in decreased abundance of enzymes involved in purine/pyrimidine synthesis and decreased abundance of IC hypoxanthine. Free glycine has a toxic effect on bacterial cells as it is being incorporated into the peptidoglycan precursor instead of alanine and ultimately weakens the cell wall and impairs cell growth^95^. Thus, due to impaired GCS evident in IC glycine detected only in chloramphenicol-treated group of NCTC13169 and increased IC glycine in NCTC8325, the cell possibly tried to maintain non-toxic levels of free glycine by increasing abundance of EC glycine observed in both species. A possibility of chloramphenicol being degraded to glycine could be further investigated, as this and altered expression of *gcs* genes has been reported in chloramphenicol-treated *Sphingomonas sp*.^110^. Thus, the glycine cleavage system possibly plays a role in survival under sub-MIC chloramphenicol stress in both Gram-positive species. Additionally, increased abundance of EC glycine may affect susceptibility to chloramphenicol. Glycine treatment of MDR *K. pneumoniae* restored susceptibility to meropenem, cefiderocol, or colistin, and restored susceptibility of *E. coli* K-12 JE2100 strain harbouring the R100-1 factor to chloramphenicol^111,112^. Thus, proper management of glycine metabolism may be critical in response to chloramphenicol.

Proteomic changes suggest an induced oxidative stress in both NCTC13169 and NCTC8325 due to exposure to sub-MIC chloramphenicol. However, NCTC8325 also experienced changes in iron homeostasis and iron storage capacity. In NCTC13169, decreased abundance of MarR could contribute greatly to cell survival under sub-MIC of chloramphenicol since its deletion in *E. faecium* caused decreased susceptibility to several antibiotics, increased biofilm formation and decreased resistance to oxidative stress^40^. Despite that, increased and decreased abundance of respective glutathione and hypoxanthine suggest an efficient metabolomic response to counteract oxidative damage^44,45^.

**Fig. 6:**
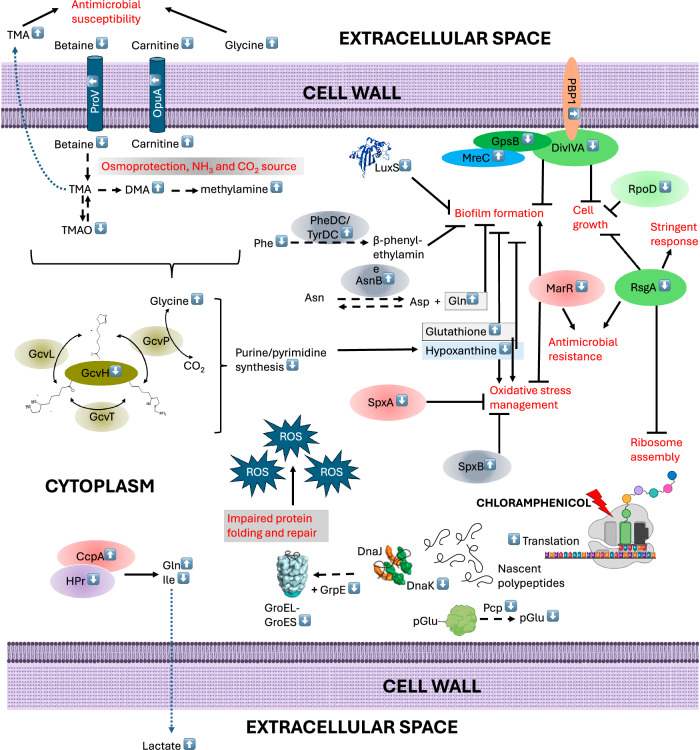
Schematic representation of hypothetical cellular mechanism that may occur upon exposure to sub-MIC of chloramphenicol in *E. faecium* NCTC13169. Proteins and metabolites with adjacent white arrow pointing up and down are increased and decreased in abundance, respectively, based on metabolomic and proteomic data obtained in this study. Continuous arrows and brackets denote positive effect, while T-shaped arrows denote negative effect. Black dashed arrow denotes enzymatic reaction, while blue dashed arrow denotes hypothetical transmembrane transport.

**Fig. 7:**
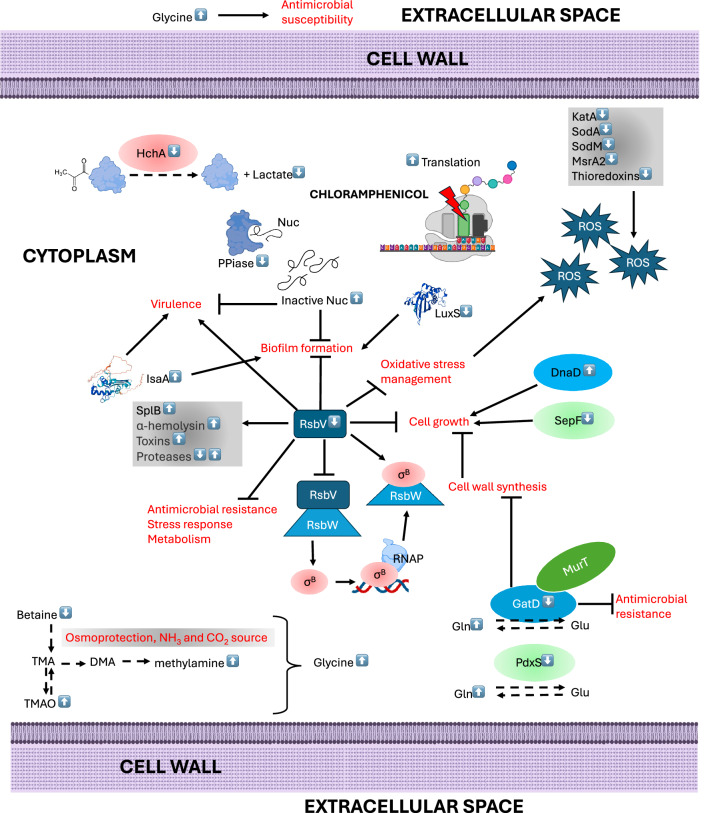
Schematic representation of hypothetical cellular mechanism that may occur upon exposure to sub-MIC of chloramphenicol in *S. aureus* NCTC8325. Proteins and metabolites with adjacent white arrow pointing up and down are increased and decreased in abundance, respectively, based on metabolomic and proteomic data obtained in this study. Continuous arrows and brackets denote positive effect, while T-shaped arrows denote negative effect. Black dashed arrow denotes enzymatic reaction.

Increased abundance of ProV homolog and OpuA indicates induced osmotic stress under sub-MIC chloramphenicol in NCTC13169, since those osmoprotectant transporters are up-regulated in response to high osmolarity^46^. They transport quaternary amines: glycine betaine, choline, carnitine and/or proline. ProV was experimentally validated as a chloramphenicol resistance gene in *Salmonella enterica* and by machine learning in *E. coli*^113^. OpuA is orthologous (64% identical) to OpuCA (Q9KHT9) from *Listeria monocytogenes* involved in carnitine uptake^114^. Thus, increased abundance of OpuA may be associated with chloramphenicol-decreased and -increased abundances of EC and IC carnitine in NCTC13169. Interestingly, decreased abundance of IC betaine was specific to chloramphenicol response in both Gram-positive species, highlighting a role of osmoprotection in response to chloramphenicol. Decreased IC betaine abundance also suggests its metabolic utilisation as a carbon and nitrogen source^115^. However, enzymes associated with betaine processing were not differentially abundant. Betaine utilisation may be linked to altered trimethylamine (TMA) metabolism detected in both Gram-positive species. TMA metabolism is common among the mammalian gut microbiome^116^, while *S. aureus* can produce TMA^117,118^. Associated staphylococcal and enterococcal genes have not been identified and functionally characterised yet. Several metagenomic and meta transcriptomic studies predicted genes involved in carnitine/choline/betaine to TMA degradation in some Firmicutes, but not Enterococcus species^116,119,120^. Human gut-isolated *E. faecalis* could degrade TMA anaerobically^121^. Also, mammalian gut reduction of TMA to TMAO by Enterococcus has been proven in mice in vivo and ex vivo^122^. To our knowledge, this study for the first time detected altered TMA metabolism as a response to chloramphenicol treatment in *E. faecium* and *S. aureus*. Overall, quaternary amines metabolism coupled with TMA metabolism may be an alternative nitrogen and carbon generating pathway aiding in survival in response to sub-MIC chloramphenicol. However, chloramphenicol-increased abundance of EC TMA in NCTC13169 could contribute to increased susceptibility to antibiotics. Gram-positive and negative bacteria treated with TMA had increased susceptibility to chloramphenicol most likely due to increased pH of extracellular space caused by TMA^123^. Also, increased abundance of IC TMAO in NCTC3825 may negatively affect survival^96^. In summary, both NCTC13169 and NCTC8325 exhibit adaptive responses to sub-MIC levels of chloramphenicol, involving translation optimization, oxidative stress management, biofilm modulation, and osmoprotection. While shared mechanisms like glycine metabolism and osmoprotectant regulation underscore conserved strategies, species-specific differences as in iron homeostasis, divisome-associated proteins, and sigmaB activity suggest nuanced adaptations tailored to their respective cellular and ecological contexts. Further experimental validation is warranted to confirm these phenotypes and elucidate their contributions to sub-MIC chloramphenicol survival. Previously discussed hypothetical cellular events that may occur upon exposure to sub-MIC of chloramphenicol are explained in Figs. 6 and 7 for *E. faecium* NCTC13169 and *S. aureus* NCTC8325, respectively.

**Fig. 8:**
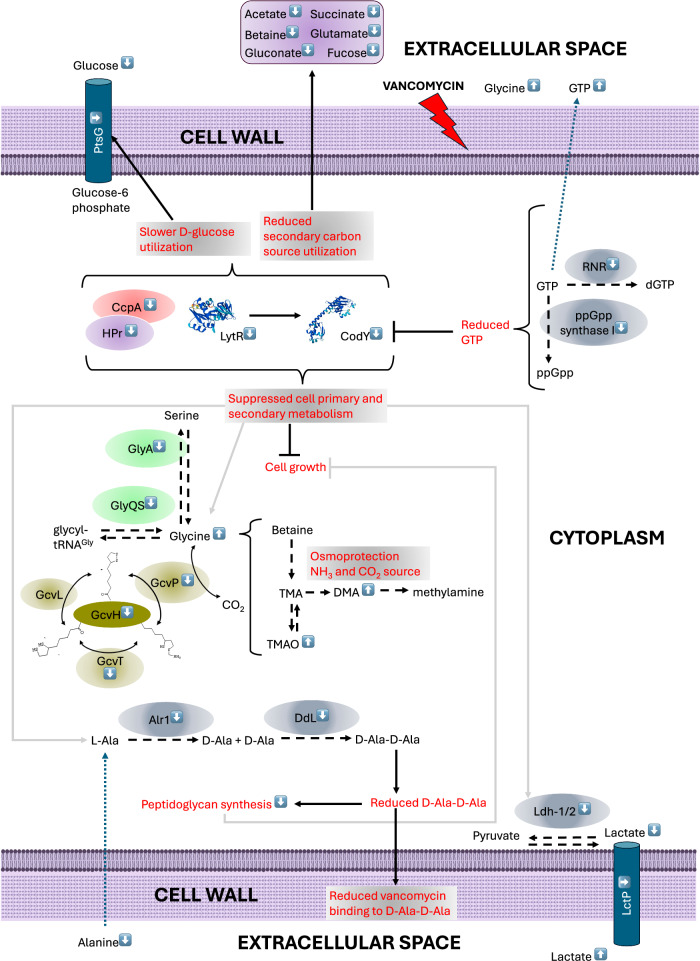
Schematic representation of hypothetical cellular mechanism that may occur upon exposure to sub-MIC of vancomycin in *S. aureus* NCTC8325. Proteins and metabolites with adjacent white arrow pointing up and down are increased and decreased in abundance, respectively, based on metabolomic and proteomic data obtained in this study. Continuous arrows and brackets denote positive effect, while T-shaped arrows denote negative effect. Black dashed arrow denotes enzymatic reaction, while blue dashed arrow denotes hypothetical transmembrane transport.

The glycopeptide vancomycin binds to the terminal D-Ala-D-Ala residue of Lipid II, an intermediate in the peptidoglycan layer maturation process, blocking access to penicillin-binding proteins (PBPs) and thereby inhibiting transglycosylation and transpeptidation reactions. That compromises the integrity of the cell wall and causes osmotic stress and ultimately cell death as previously reviewed^124^. Results in this study imply that survival of *S. aureus* NCTC8325 exposed to sub-MIC of vancomycin is primarily managed via decreased abundance of global regulators LytTR, CodY and CcpA. Suppression of those caused primary and secondary metabolism to slow down, which is reflected in proteomic and metabolomic results. Previous study reported that CodY- and CcpA-associated regulons are responsive to sub-lethal concentration of vancomycin in *S. aureus*^125^. Despite decreased abundance of CcpA, decreased activity of CcpA is also evident in decreased abundance of HPr kinase necessary for activation of HPr protein that enhances binding affinity of CcpA to a promoter of target genes as described for *Bacillus subtilis*^126^. Deletion of staphylococcal *ccpA* and *codY* caused reduced utilisation of D-glucose and increased utilisation of secondary carbon sources like acetate^72^, which is in line with vancomycin-decreased abundance of EC acetate but not with that of decreased abundance of EC glucose. Thus, EC glucose is being more utilised but likely at a slower pace compared to the untreated control due to vancomycin-decreased phosphotransferase system and glycolysis/gluconeogenesis pathway. EC succinate, gluconate, betaine, fucose and glutamate are decreased in abundance and potentially utilised as a secondary carbon source aiding in survival under sub-MIC vancomycin stress. Moreover, fucose may be utilised as a building unit of the cell wall^127^. Altered abundance of choline and O-phosphocholine implicated in teichoic acid synthesis suggests perturbances in teichoic acid synthesis that could affect susceptibility of *S. aureus* to vancomycin^128,129^. Also, trimethylamine metabolism was altered by vancomycin, highlighting its important role in antibiotic stress survival in *S. aureus*. As previously mentioned, increased abundance of IC TMAO may negatively affect survival^96^. Overall, the systematic suppression of cellular metabolic pathways and cellular growth machinery including peptidoglycan biosynthesis and D-Ala-D-Ala biosynthesis targeted by vancomycin likely puts NCTC8325 in a dormant state enabling survival to sub-MIC vancomycin^130^. The potentially beneficial effect of decreased abundance of CodY on sub-MIC vancomycin survival was already corroborated in staphylococcal *codY* deletion mutants showing enhanced resistance to cell-wall targeting antibiotics, including vancomycin and slower growth in vitro^131^. CodY activity is regulated by branched-chain amino acids and GTP, thus vancomycin-increased abundance of EC GTP may be a strategy to maintain low levels of active CodY^74^. Also, we observed a decrease in GTP pyrophosphokinase ((p)ppGpp synthase) involved in synthesis of ppGpp, which is involved in stringent response functionally linked to CodY. That should result in decreased abundance of ppGpp playing a pivotal role in mediating resistance to antibiotics, including vancomycin in *S. aureus*^132,133^. Decreased abundance of CodY and CcpA is likely to negatively affect biofilm formation and virulence under sub-MIC of vancomycin^72^. CodY is a repressor of the *sae* operon, thus decreased abundance of CodY should affect virulence pathways regulated by the SaeR/SaeS two-component regulatory system^134^. However, SaeS abundance is reduced in vancomycin treated *S. aureus*, thus the amount of active SaeR should be reduced, which should affect its ability to activate transcription of the *sae* operon^134^. Also, sigma B regulators RsbW and RsbV were reduced in abundance in the presence of vancomycin, which should affect activity of Sigma-B factor implicated in regulation of the *sae* operon^135^. SaeR target genes coded by the *sae* operon^136^ were not vancomycin-altered in our study, which could be due to decreased abundance of SaeS, CodY, and Sigma-B regulators resulting in coordinated regulation of the virulence genes coded by the *sae* operon. As previously mentioned, increased abundance of IC glycine associated with a decreased abundance of glycine cleavage system should negatively affect survival due to glycine bactericidal activity^95^. Increased abundance of IC glycine upon 4x MIC of vancomycin treatment in *S. aureus* was already reported^137^. The study also reported supressed glycolysis/gluconeogenesis and purine biosynthesis pathways, similar to our findings, and decreased uptake of EC glycine and EC glutamate, which is in line with increased abundances of those extracellular metabolites in our study. Further, purine metabolism was proven to play a significant role in antibiotic resistance in *S. aureus*^138^. A decreased abundance of purine nucleoside phosphorylase DeoD2 and altered abundance of associated purines could affect susceptibility of *S. aureus* to vancomycin since *deoD2* mutants significantly increased β-lactam resistance in methicillin-resistant *S. aureus*^138^. Enzymes involved in TCA metabolism play a significant role in resistance to antibiotics in *S. aureus*, and decreased abundance of succinate-CoA ligase subunits SucC and SucD could affect susceptibility to vancomycin as deletion of these enzymes altered antibiotic susceptibility in *S. aureus*^139,140^. Schematic representation of hypothetical cellular events in *S. aureus* NCTC8325 upon exposure to sub-MIC of vancomycin is visualised in Fig. 8.

In conclusion, this study delved into the poorly understood complexity and diversity of bacterial adaptive responses to sub-inhibitory concentrations (sub-MIC) of a range of different antibiotics, emphasizing the interplay between proteomic and metabolomic changes. While shared adaptive mechanisms, such as alterations in glycine and trimethylamine metabolism, were observed across species, species-specific responses reflected adaptations to antibiotic-induced stress. The identification of key proteins, such as ciprofloxacin-responsive SbmC in *K. pneumoniae*, as well as chloramphenicol- and vancomycin-specific adaptations in Gram-positive species, provides novel information on bacterial responses to antibiotics outside the direct mutations or up-regulation of efflux. These mechanisms are associated directly with survival in sub-MIC antibiotics or the mutational development of resistance to antibiotics. This study underscores the interconnectedness of metabolic and proteomic pathways and their roles in bacterial survival under antibiotic stress, offering promising avenues for future research and therapeutic intervention.

## Methods

### Strains and culture media

The following antibiotic-susceptible strains were used for all experiments: *Escherichia coli* MG1655*, Klebsiella pneumoniae* NCTC418, *Enterococcus faecium* NCTC13169 and *Staphylococcus aureus* NCTC8325. These susceptible strains were chosen due to well characterised genomes and proteomes. For all proteomics and metabolomics experiments, strains were cultured in a modified M9 minimal medium containing 1X M9 salts solution (M6030, Sigma Aldrich), 0.4% glucose, 2 mM MgSO_4_, 0.1 mM CaCl_2_ and 0.5% yeast extract. This media was chosen instead of nutrient rich media to mimic natural bacterial environment with low glucose concentration, and to reduce the effect of nutrients present in the media on the metabolome and metabolomic analysis. Yeast extract was added in the media to allow growth of Gram positive (+) species^26^. For minimum inhibitory concentration (MIC) assays, strains were initially cultured overnight in Luria-Bertani (LB) broth (L3022-Sigma Aldrich). Assays were then performed in 96 well plates using cation-adjusted Mueller Hinton broth 2 (MH 2 broth) (90922, Millipore). Overnight and diluted cultures were normally grown in aerobic conditions in culture tubes and non-baffled flasks, respectively, in a flask/tube-to-medium ratio 5:1.

### Minimum inhibitory concentration (MIC) assays

All MIC assays were performed in accordance with CLSI guidelines^141^. Shortly, colonies were inoculated in LB broth (4 mL) overnight at 225 RPM and 37 °C. A 100 μl of antibiotics diluted at a concentration 8x the breakpoint concentration was pipetted in all wells of column 1 of the 96-well plate, and 50 μl of MH 2 was added in all wells in columns 2-12. Microdilutions of the antibiotic were performed by aliquoting 50 μl of antibiotic solution starting from the column 1. To prepare inoculum, cultures were 100x diluted and incubated for 3–4 h at 225 RPM and 37 °C. Cultures were further diluted to OD_600_ = 0.002. Then, 50 μL of inoculum was added to all wells in rows A-G of a 96-well plate containing doubling dilutions of antibiotic, and row H was used as a negative control. The plate was incubated for 18 h at 37 °C in a static incubator and the MIC values were determined as the lowest concentration that inhibited bacterial growth.

### Bacterial cultures growth and treatment

Similar culture growth and treatment conditions were used for extractions of proteins and metabolites. Isolated colonies were cultured overnight in three biological replicates of 5 mL and 3 mL M9 minimal medium at 225 RPM and 37 °C for metabolite and protein extractions, respectively. For metabolite extractions, cultures were diluted in a total of 100 mL M9 minimal medium and incubated at 225 RPM and 37 °C until they reached OD_600_ = 0.35. For protein extractions, cultures were diluted to OD_600_ = 0.2 and incubated at 225 RPM and 37 °C until they reached OD_600_ = 0.35. Antibiotics were added to the cultures at a concentration of 0.5 MIC specific for each antibiotic and strain, while equal volume of water was used as a negative control. Samples were incubated at 225 RPM and 37 °C for 1 h, a duration shown to be sufficient for eliciting early metabolic responses while keeping cells in the exponential phase when their proteome and metabolome is the most active^142,143^. This approach also minimizes the risk of antibiotic-induced cell death and avoids confounding effects associated with the onset of the post-exponential or stationary phase^143–145^.

### Isolation of intracellular and extracellular metabolites

After 1 h of treatment incubation, a 100 mL of culture was centrifuged at 3000 RPM for 10 minutes at 4 °C. To isolate extracellular metabolites (EM), supernatant (20 mL) was filtered through a 0.2 μm filter and frozen with liquid nitrogen. These EM extracts were then lyophilised and stored at −80 °C. To isolate intracellular metabolites (IM), cells were quenched using previous protocol^146^ with some variations. Methanol (800 μL stored at -80°C) and ice-cold double distilled water (170 μL) were used to resuspend the cell pellet and to quench the cells. The resuspended sample was vortexed followed by sonication in a water bath for 10 minutes. Chloroform (800 μL stored at −80 °C) and ice-cold double distilled water (400 μL) were added to the sample, vortexed and incubated on ice for 15 min. Samples were then centrifuged for 20 minutes at 4 °C, 3000 RPM. The top aqueous methanol layer containing the intracellular metabolites was dried in a SpeedVac. The dried extracts were stored at −80 °C.

### Acetone precipitation

Dried sample was resuspended in 250 μL of ice-cold nuclear magnetic resonance (NMR)-grade deuterium oxide (Thermo Scientific) and acetone (1.25 mL stored at −80°C)^146^. Samples were incubated at −80 °C overnight, thawed on ice and centrifuged at 2000 RPM for 30 minutes at 4 °C. The sample was transferred to a new 2 mL tube and dried in a SpeedVac. Dried samples were stored at −80 °C.

### Sodium periodate (NaIO_4_) treatment

NaIO_4_ treatments were performed according to ref. ^147^. Prior to treatment, all samples were equilibrated to room temperature. All IM samples were resuspended in deuterium oxide (750 μL) containing 1 mM 3-(Trimethylsilyl)-1-propanesulfonic acid sodium salt (DSS) (Sigma Aldrich) and NaIO_4_ (50 mM; Sigma Aldrich). All EM samples were resuspended in deuterium oxide (750 μL) containing DSS (1 mM) and NaIO_4_ (300 mM). Samples were incubated in the dark at room temperature for 4 hours. Glass Pasteur pipettes were used to add samples into NMR tubes (Norell).

### NMR data acquisition and analysis

1D ^1^H NMR spectra were recorded using a Bruker Ascend 500 spectrometer (500 Mhz) at 293 K. ^1^H NMR spectra were acquired using the Bruker-automated pulse program ZG30 over 16 scans in NMR grade deuterium oxide with DSS as internal standard. ^1^H NMR spectra were Fourier transformed and processed using TopSpin Sofware (3.0). Chemical shifts were reported in parts per million (ppm). All ^1^H NMR spectra were processed and profiled using the Chenomx NMR Suite software version 9.0 (Chenomx, Inc. Alberta, Canada). The Chenomx Processor module was used to manually correct the spectral data, while the Profiler module was used to identify and quantify metabolites and to generate a fitted spectrum for each sample. The concentrations of the metabolites were quantified by using DSS (1 mM) as an internal standard. Several identified compounds were deemed contaminants/incorrect match and were removed from further analysis. Those were synthetic compounds (N,N-dimethylformamide, propylene glycol, 2-ethylacrylate) and human or plant metabolites (chlorogenate, vanillate, 4-aminohippurate, tiglylglycine and caffeine). Metabolites that were identified in at least two biological replicates in at least one experimental group were kept for the analysis. MetaboAnalyst 6.0^148^ was used for data processing and visualisation of Principle component analysis (PCA) score plots and Volcano plots. Samples with >50% missing values were removed from the analysis and all missing values were replaced with 1/5 of the minimum value of the corresponding sample. Normalization method that resulted with a normal distribution of the data was chosen and varied between datasets (Supplementary Data 1). Principal component analysis score plots were calculated on normalized data. T-test with unequal variance, log_2_ fold change (FC) >|1| and false discovery rate (FDR) < 0.05 was performed on each normalized dataset to identify significant differentially abundant metabolites. To produce heatmaps missing values were replaced with zeros and visualised in SRplot web-based tool^149^ with default parameters.

### Protein extractions

After the 1 h of treatment incubation, 1 mL of each culture was centrifuged at 13000 RPM for 2 minutes at room temperature. The supernatants were discarded, and cell pellets were resuspended in 1 mL deionised water. The samples were centrifuged again using the same settings. The supernatants were discarded, and the pellets were resuspended in 1.2 mL of 75% ethanol, vortexed for 5 min and centrifuged at 16,000 × *g* for 2 min. The supernatants were discarded, and the pellets were air dried at room temperature for 10 min. Then, 100 μL of 70% formic acid was added to each pellet, vortexed briefly and incubated at room temperature for 5 min. Following this, 100 μL of acetonitrile was added to each sample and samples were vortexed briefly and centrifuged at 16,000 × *g* for 2 min. For each sample, the supernatant was added to 5 volumes of ice-cold acetone and incubated at −20 °C overnight. Samples were centrifuged at 10,000 × *g* for 5 min at 4 °C, the supernatants were discarded, and pellets were air dried for 30 minutes.

### Sample preparation and analysis for LC-MS/MS protein identification

Preparation of protein samples for LC-MS/MS protein identification was performed as described by^150^, with following variations: samples were incubated in iodoacetamide in the dark for 20 instead of 15 minutes; After incubation in trifluoroacetic acid (TFA), samples were immediately centrifuged and dried in a SpeedVac for 2 h; Dried peptide samples resuspended in 0.5% TFA were sonicated for 5 instead of 3 minutes, followed by a brief centrifugation. A 500 ng of each peptide sample was loaded on a high-resolution quantitative LC-MS (Thermo Fisher Q-Exactive). Loading of the samples and LC-MS/MS analysis was performed as previously described^151^, with the exception that elution time was 90 minutes. Each individual experimental set of specific antibiotic- and water-treated sample groups were ran in the same run and analysed separately of other experimental sets.

### Protein identification, data processing and visualisation

Proteome FASTA files for *E. coli* MG1655 (Proteome ID: UP000000625) and *S. aureus* NCTC8325 (Proteome ID: UP000008816) were downloaded from the Universal Protein Knowledgebase (UniprotKB) web database^152^; proteome FASTA files for *K. pneumoniae* NCTC418 (RefSeq ID: GCF_900635995.1) and *E. faecium* NCTC13169 (RefSeq ID: GCF_900447945.1) were downloaded from the National Center for Biotechnology Information (NCBI) web database^153^. Protein identification and label-free quantification (LFQ) normalization were performed using MaxQuant v1.5.2.8 quantitative proteomic software^154^ with default parameters. Amica web-based platform was used for proteomics LFQ output quality control, protein differential abundance analysis and data visualisation. The ‘minimum MS/MS counts’ and ‘minimum razor/unique peptides’ parameters were set to 3 and 2, respectively. Only proteins identified in a at least two replicates in at least one treatment group were kept for analysis. Randomized sampling from a normal distribution with 1.8 downshift and 0.3 width for each sample was used as an imputation method. Imputation of missing values was avoided when it significantly reduced intra-group correlation, as shown by Pearson correlation or PCA score plots, or when the median coefficient of variation (CV) exceeded 30% post-imputation. Differential Expression analysis of quantitative Mass Spectrometry data (DEqMS)^155^ was used for differential abundance analysis with log2FC > |1| and FDR < 0.05 cutoffs.

### Gene ontology (GO)/Kyoto Encyclopaedia of Genes and Genomes (KEGG) pathway enrichment analysis and KEGG pathway analysis

Gene ontology and KEGG pathway enrichment analysis of differentially abundant proteins (DAPs) was performed with DAVID^156^ with default parameters, using the Benjamini–Hochberg-adjusted *P*-value threshold of 0.05. Proteins that were increased or decreased in abundance were analysed separately for enrichment. For *E. faecium* NCTC13169, RefSeq accessions of proteins were converted to UniProt accession in UniProtKB with Taxonomy filter for *E. faecium*. Hits with UniParc accessions were BLASTed in UniProtKB against UniProt ‘eubacteria’ and the top BLAST hit with E-value less then 0.001 and minimum 90% identity was used as a gene ID conversion hit. The test set was a list of significant DAPs and the reference set was the entire genome for the corresponding bacterial species. To study hierarchical descent more informative GO terms, enriched FAT GO terms were analysed and the list of significant terms was reduced to semantically non-redundant list of representative FAT GO terms with REVIGO^157^. The enriched KEGG pathways and representative FAT GO terms were visualized in R using the script described by ref. ^158^. The ‘Search’ tool in the KEGG mapper^159^ was used to aid in integrated analysis of DAPs and differentially abundant metabolites. KEGG gene IDs or KEGG orthology (KO) IDs and KEGG compound IDs were used as an input for *S. aureus* NCTC8325 and *E. faecium* NCTC13169, respectively. KofamKOALA was used for KO assignments with default parameters. Detailed functional annotation for each differentially abundant protein was downloaded from UniProtKB.

### Protein network analysis

STRING database^160^ was used to create protein networks of DAPs. A list of UniprotKB accessions for *S. aureus* NCTC8325 or protein FASTA sequences for *E. faecium* NCTC13169 were used as an input for mapping DAP sequences onto STRING identifiers. To focus on highly homologous hits, FASTA query sequences with less than 70% identity to the hit sequence were excluded from the network analysis. The protein network was created with default parameters except for the confidence score being set to 0.7 to investigate only high-confidence interactions. STRING network was further analysed and visualised in Cytoscape software^161^ to identify GO-terms, KEGG pathways and functionally predicted STRING local neighbourhood clusters significantly enriched (FDR < 0.05) with DAPs. Selected non-redundant terms (redundancy cutoff set to 0.7) enriched with DAPs were highlighted in the STRING network. DAPs were manually re-arranged into sub-networks based on their functional annotation. For the purposes of exploring function of sub-networks not annotated after enrichment analysis of the whole DAPs network, those were individually analysed for functional enrichment (FDR < 0.05). Singletons, proteins that lack a protein partner, were excluded from the network unless they belonged to a group of proteins associated with an enriched term and forming a sub-network.

## Supplementary information

Supplementary information

Supplementary data 1

Supplementary data 2

Supplementary data 3

Supplementary data 4

Supplementary data 5

## Data Availability

The mass spectrometry proteomics data have been deposited to the ProteomeXchange Consortium via the PRIDE partner repository with the dataset identifier PXD061388.
