# Supplementary material for: Proteomic and metabolomic responses of priority bacterial pathogens to subinhibitory concentration of antibiotics
Source: NPJ Antimicrob Resist. 2025 Sep 16;3:80. doi: 10.1038/s44259-025-00147-7 (PMC12441150; doi:10.1038/s44259-025-00147-7)
Supplement: Supplementary file 1 — Supplementary information [file 44259_2025_147_MOESM1_ESM.pdf]

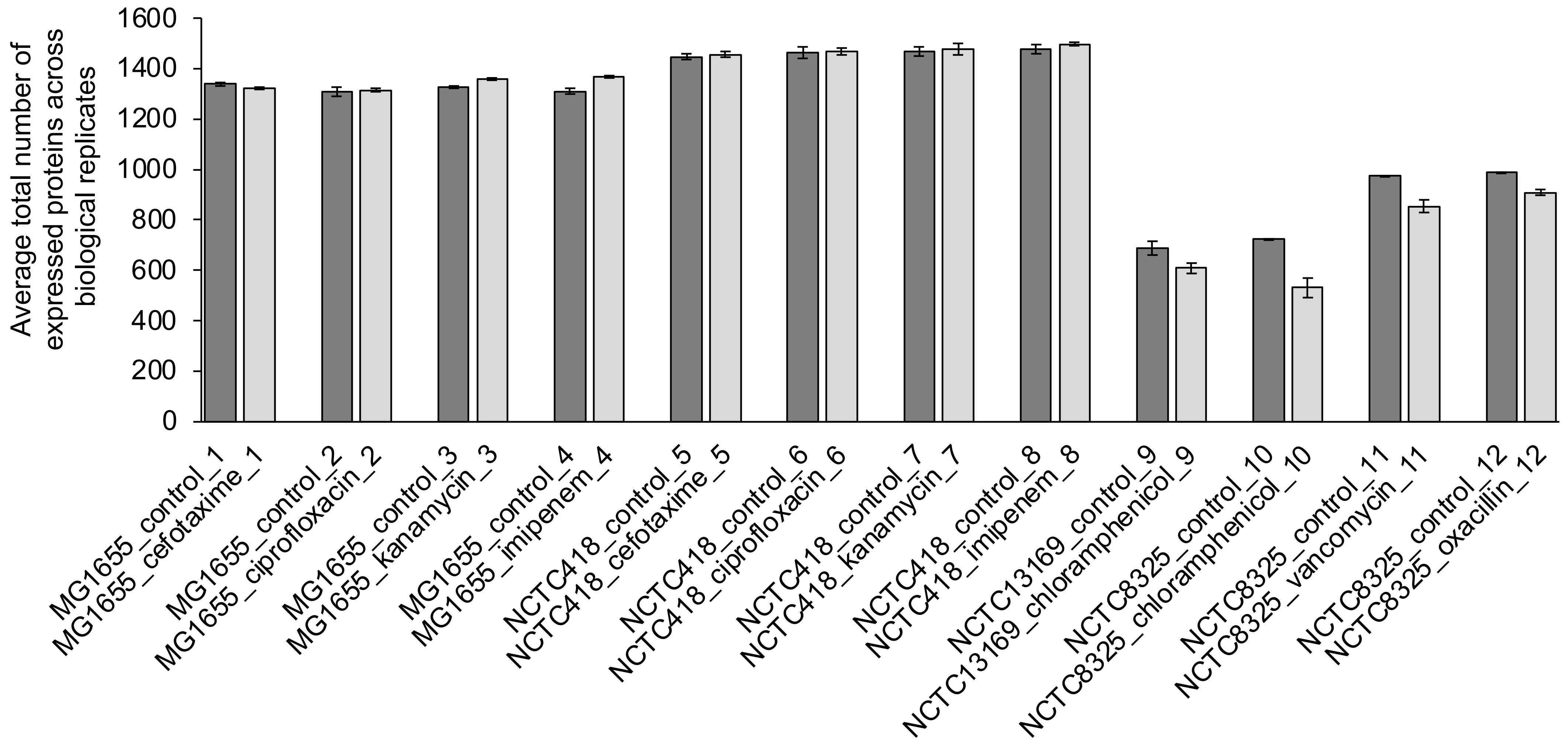

Supplementary Figure 1. Average total amount of proteins across all replicates in 12 experimental groups determined prior to filtering and imputation. Results represent the mean of three biological replicates and error bars indicate ± SEM.

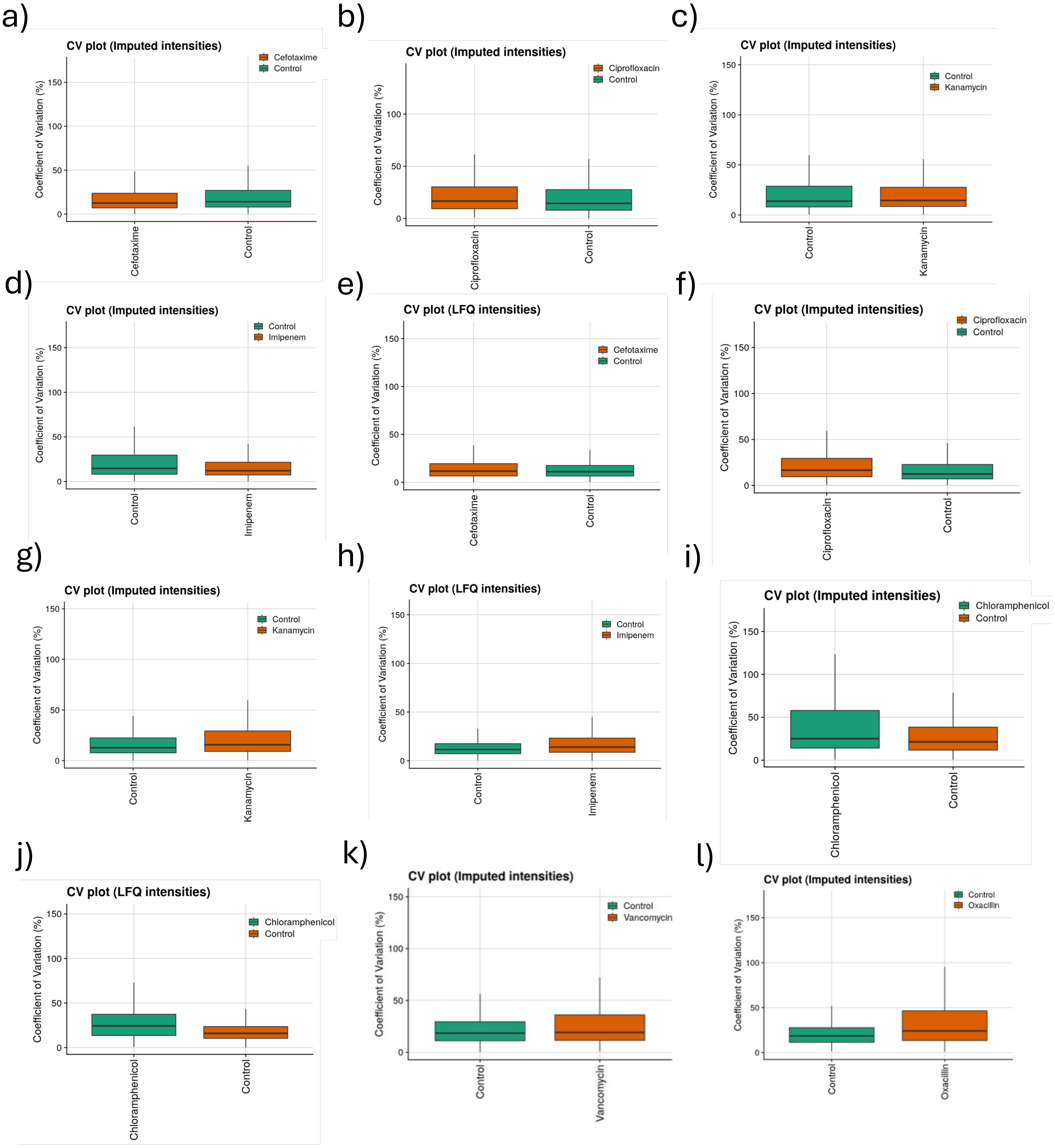

Supplementary Figure 2. Percent of coefficient of variation (CV) distribution of raw or imputed LFQ intensities in sub-MIC antibiotic-treated vs. control biological samples. Figures present CV plots of data obtained from **(a-d)** *E. coli* MG1655, **(e-h)** *K. pneumoniae* NCTC418, **(i)** *E. faecium* NCTC13169, and **(j-l)** *S. aureus* NCTC8325. Coefficient of variation (CV) plots gets calculated by the standard deviation of replicates divided by their mean per protein.

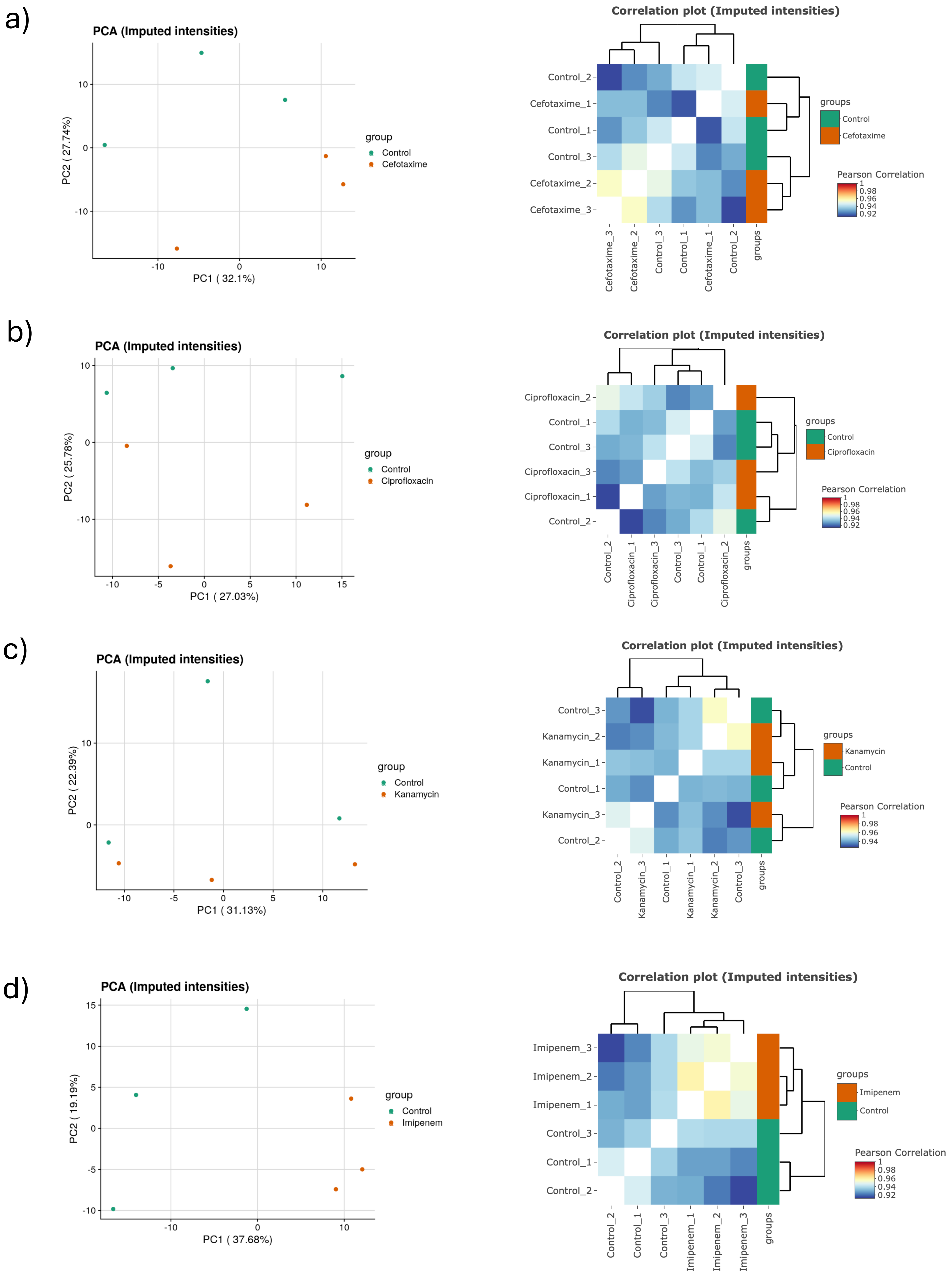

Supplementary Figure 3. Principal component analysis (PCA) score plots and heatmaps of Pearson correlation coefficients calculated on raw or imputed LFQ intensities in sub-MIC antibiotic-treated vs. control biological samples. PCA plots were calculated on data obtained for *E. coli* MG1655: **(a)** cefotaxime vs. control, **(b)** ciprofloxacin vs. control, **(c)** kanamycin vs. control, **(d)** imipenem vs. control.

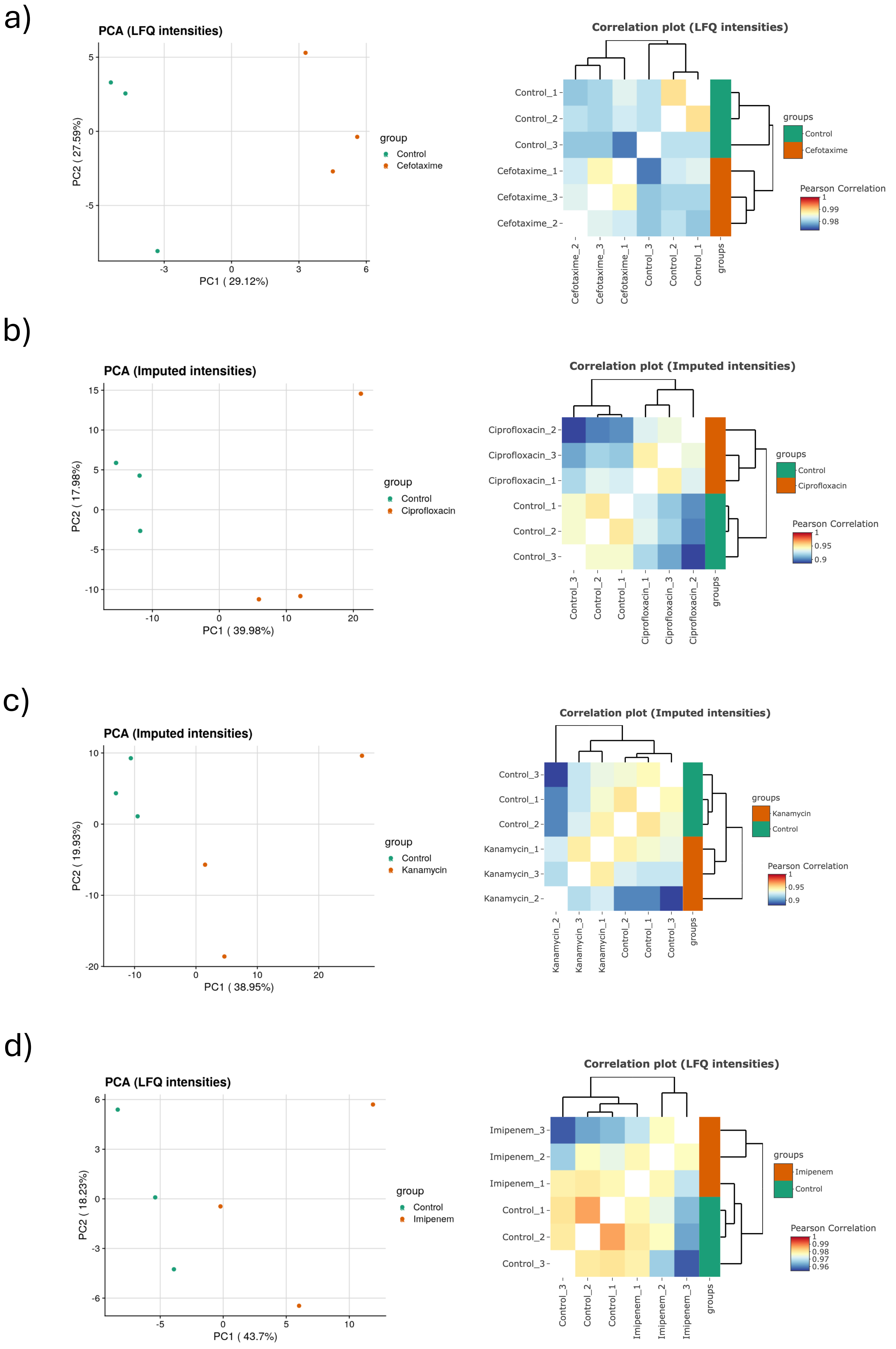

Supplementary Figure 4. Principal component analysis (PCA) score plots and heatmaps of Pearson correlation coefficients calculated on raw or imputed LFQ intensities in sub-MIC antibiotic-treated vs. control biological samples. PCA plots were calculated on data obtained for *K. pneumoniae* NCTC418: **(a)** cefotaxime vs. control, **(b)** ciprofloxacin vs. control, **(c)** kanamycin vs. control, **(d)** imipenem vs. control.

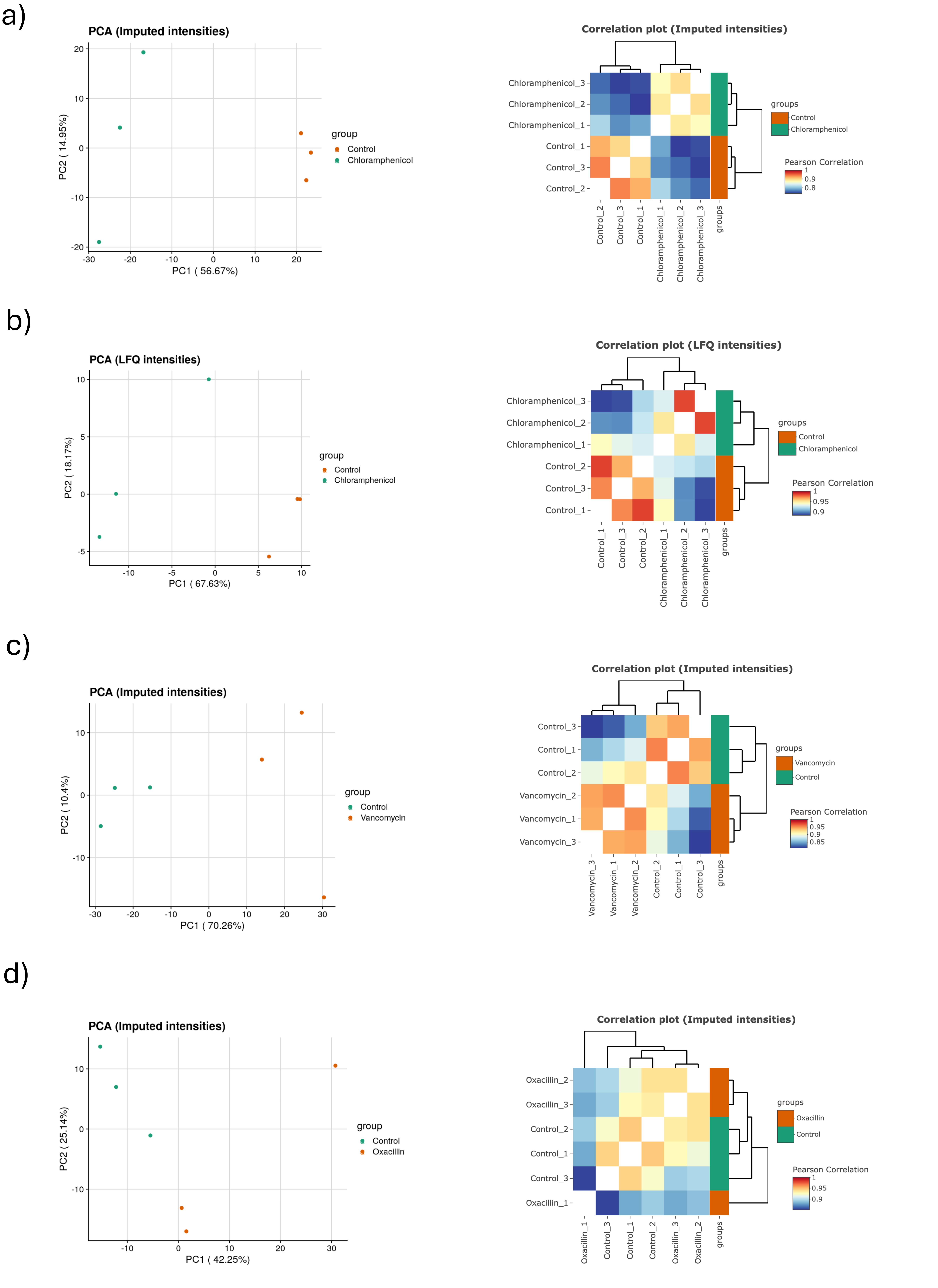

Supplementary Figure 5. Principal component analysis (PCA) score plots and heatmaps of Pearson correlation coefficients calculated on raw or imputed LFQ intensities in sub-MIC antibiotic-treated vs. control biological samples. PCA plots were calculated on data obtained for **(a)** *E. faecium* NCTC13169 and **(b-d)** *S. aurues* NCTC8325**: (a,b)** chloramphenicol vs. control, **(c)** vancomycin vs. control, **(d)** oxacillin vs. control.

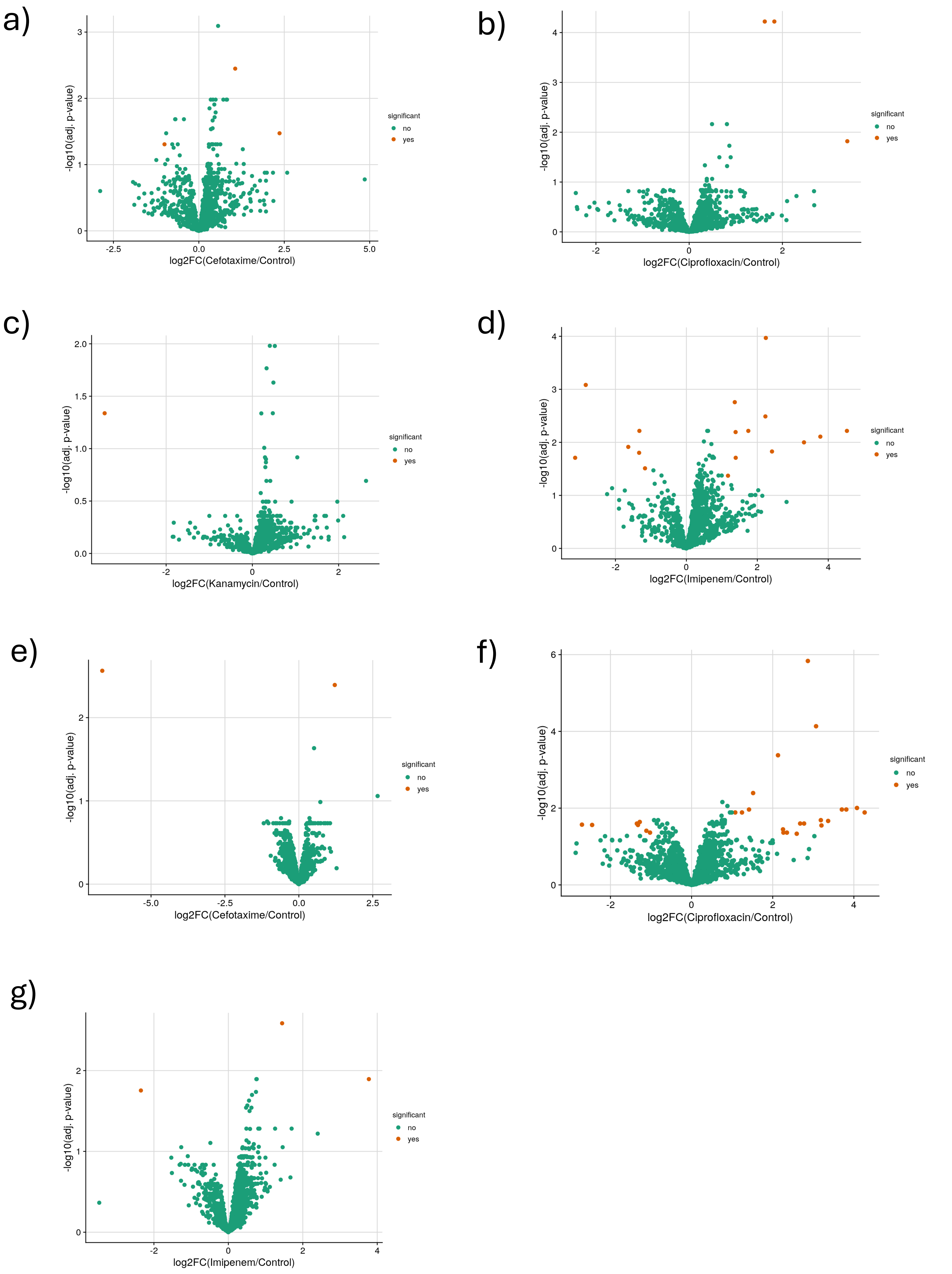

Supplementary Figure 6. Volcano plots of significantly differentially abundant proteins (DAPs). Figures **(a-d)** correspond to data obtained for *E. coli* MG1655 and **(e-g)** for *K. pneumoniae* NCTC418 treated with sub-MIC of antibiotic: **(a, e)** cefotaxime, **(b, f)** ciprofloxacin, **(c)** kanamycin, **(d, g)** imipenem. The $-\log_{10}$ (adj. *P*-value) is plotted against the log2FC between antibiotic- vs. control-treated groups. Orange- and green-coloured dots denote significant and non-significant DAPs, respectively. Missing values were imputed prior differential abundance analysis for **(a-d)** and **(f)** experimental groups.

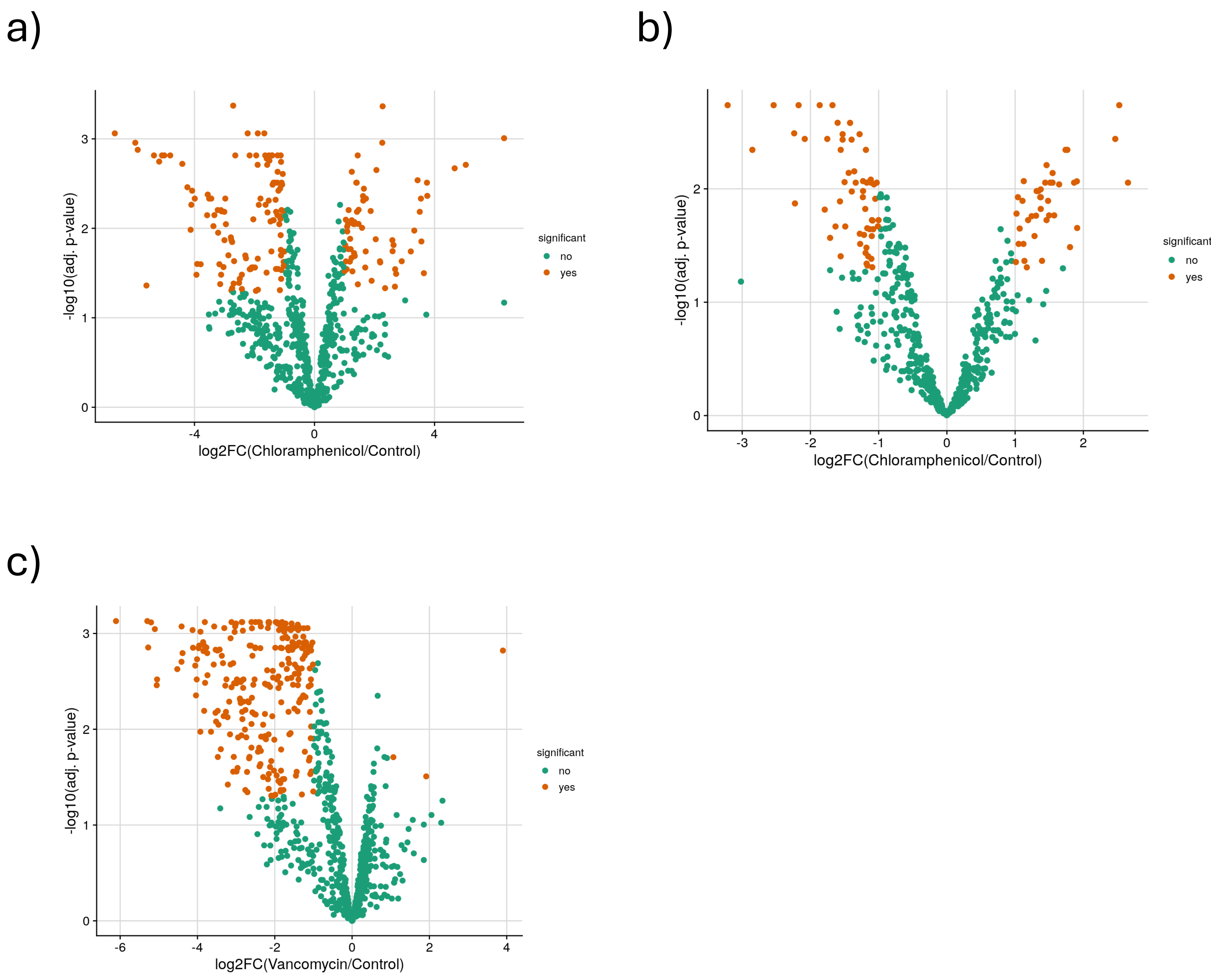

Supplementary Figure 7. Volcano plots of significantly differentially abundant proteins (DAPs). Figure **(a)** corresponds to data obtained for *E. faecium* NCTC13169 and **(b-c)** for *S. aureus* NCTC8325 treated with sub-MIC of antibiotic: **(a-b)** chloramphenicol, **(c)** vancomycin. The $-\log_{10}$ (adj. *P*-value) is plotted against the log2FC between antibiotic- vs. control-treated groups. Orange- and green-coloured dots denote significant and non-significant DAPs, respectively. Missing values were imputed prior differential abundance analysis for **(a)** and **(c)** experimental groups.

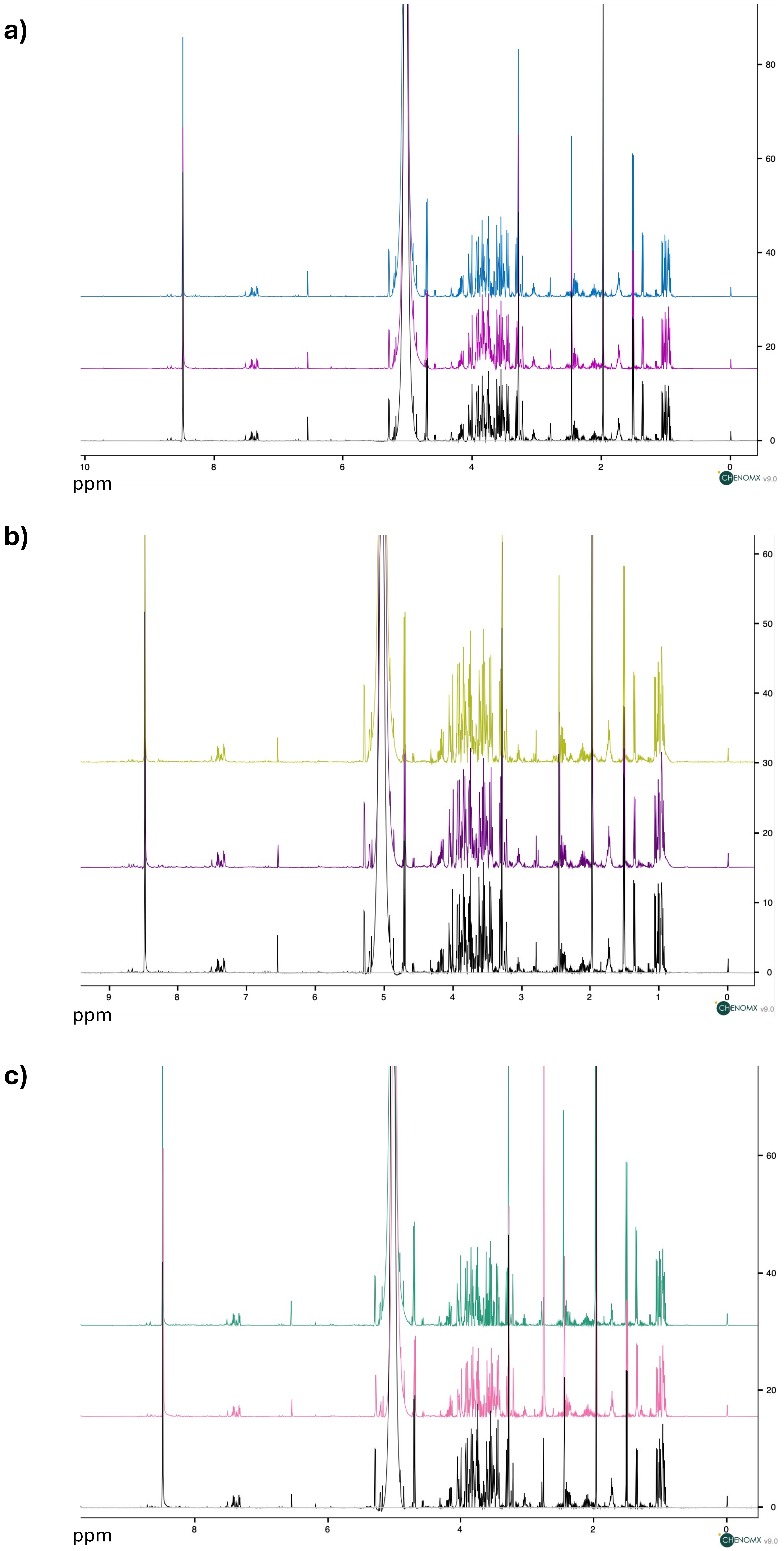
a)
80
60
40
20
0
10
8
6
4
2
0
ppm
CHENOMX v9.0
b)
60
50
40
30
20
10
0
9
8
7
6
5
4
3
2
1
0
ppm
CHENOMX v9.0
c)
60
40
20
0
8
6
4
2
0
ppm
CHENOMX v9.0

d)

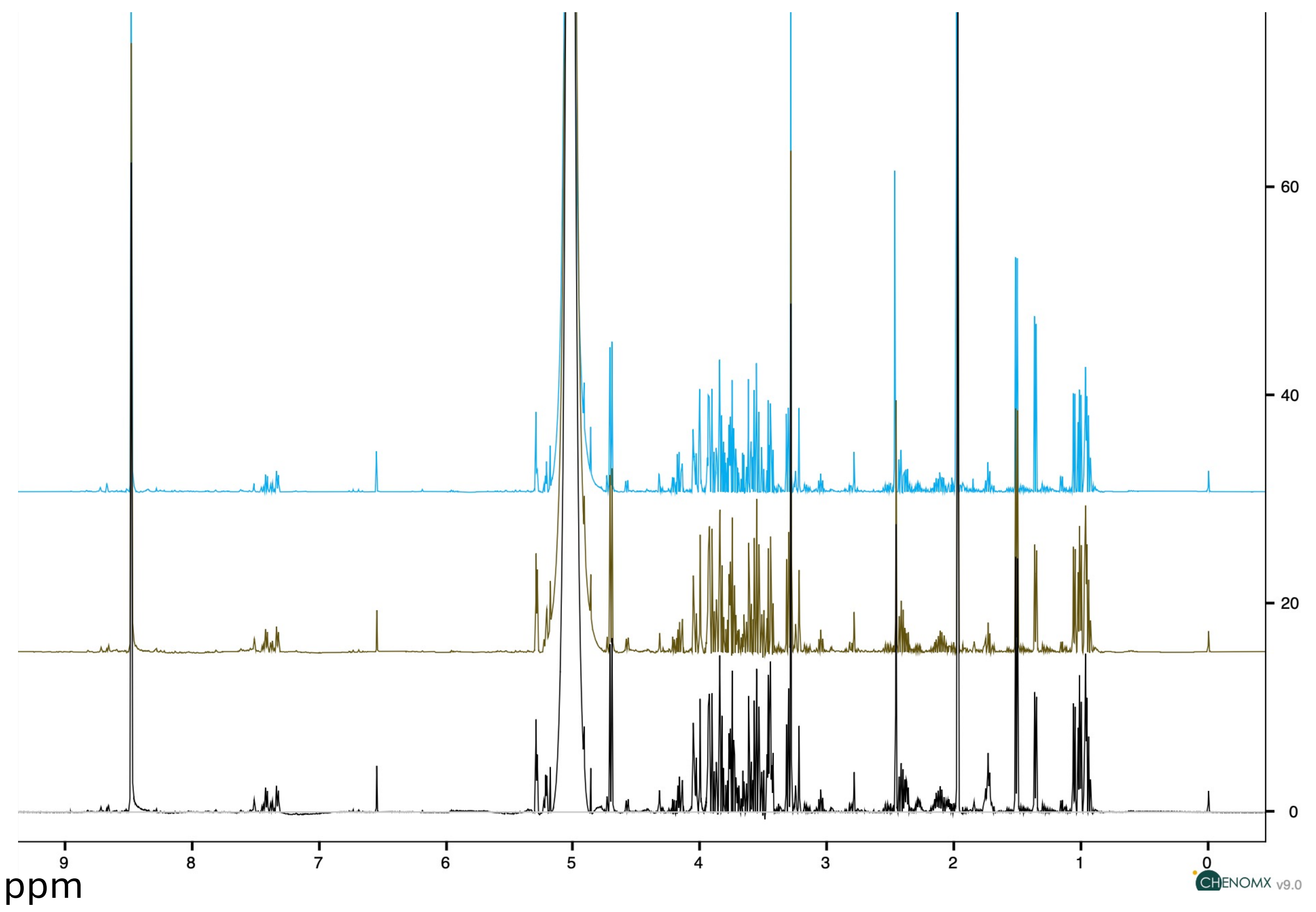

60
40
20
0
9 8 7 6 5 4 3 2 1 0
ppm

e)

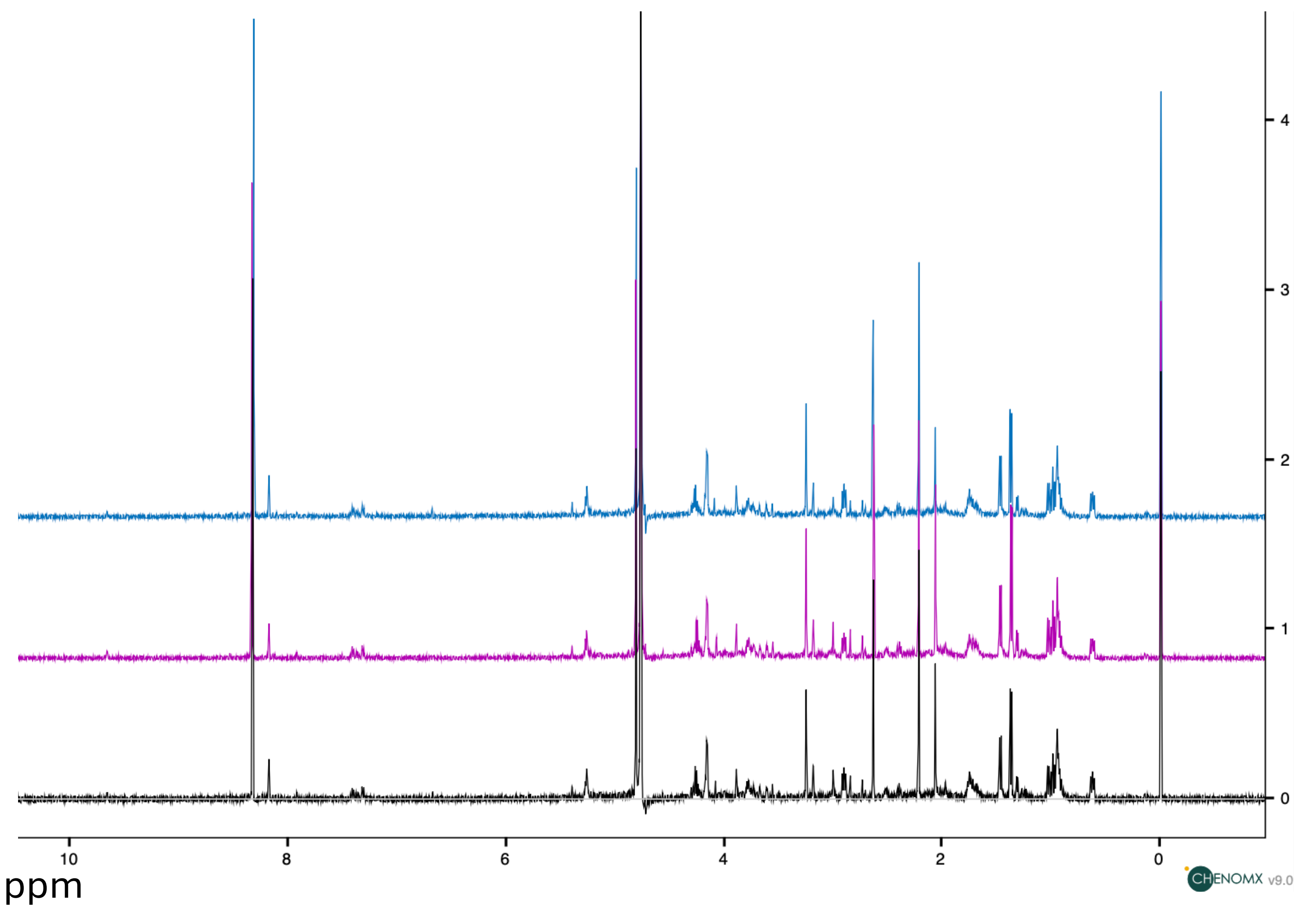

4
3
2
1
0
10 8 6 4 2 0
ppm

f)

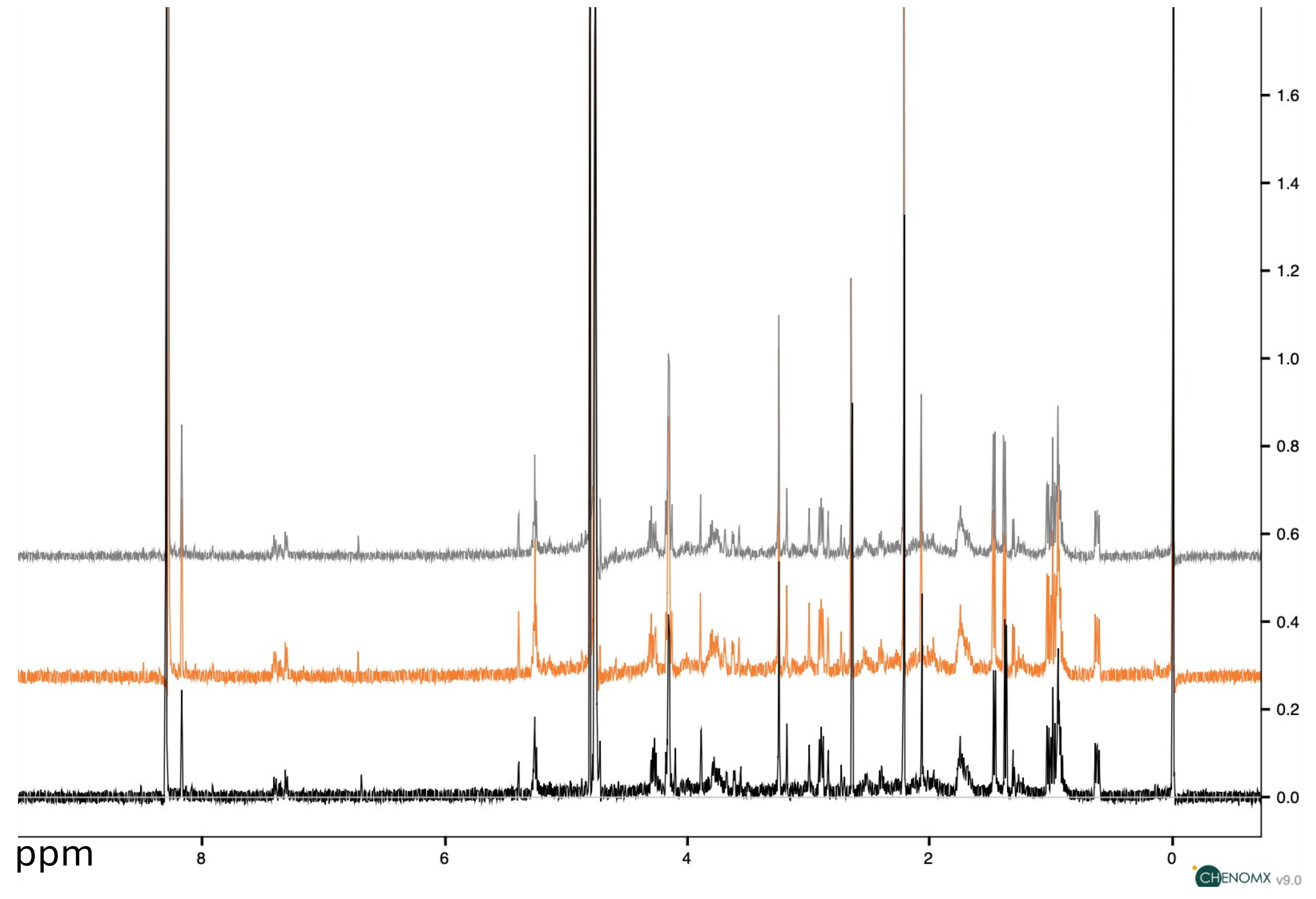

1.6
1.4
1.2
1.0
0.8
0.6
0.4
0.2
0.0
8 6 4 2 0
ppm

g)

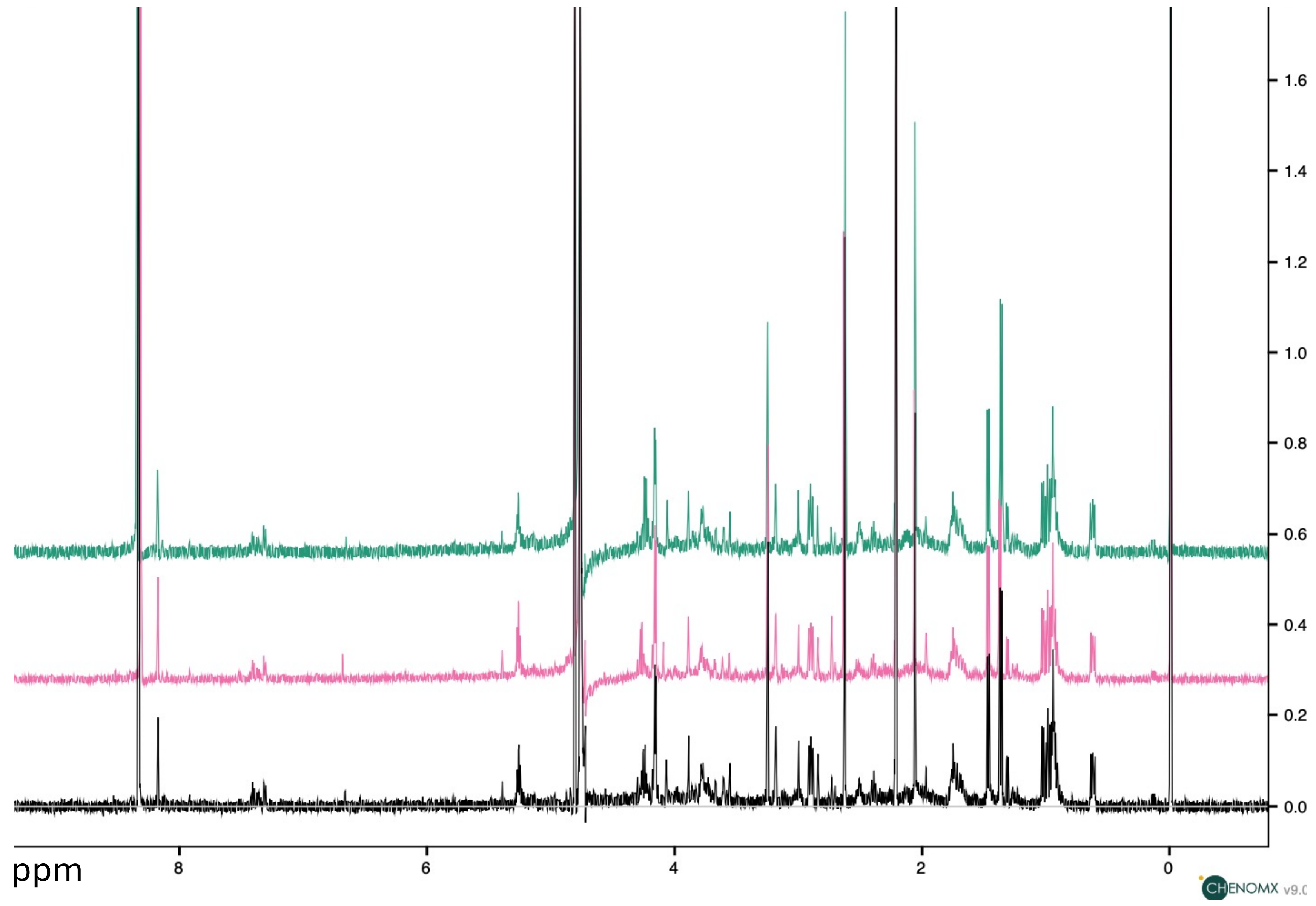
ppm
8
6
4
2
0
1.6
1.4
1.2
1.0
0.8
0.6
0.4
0.2
0.0
CHENOMX v9.0

h)

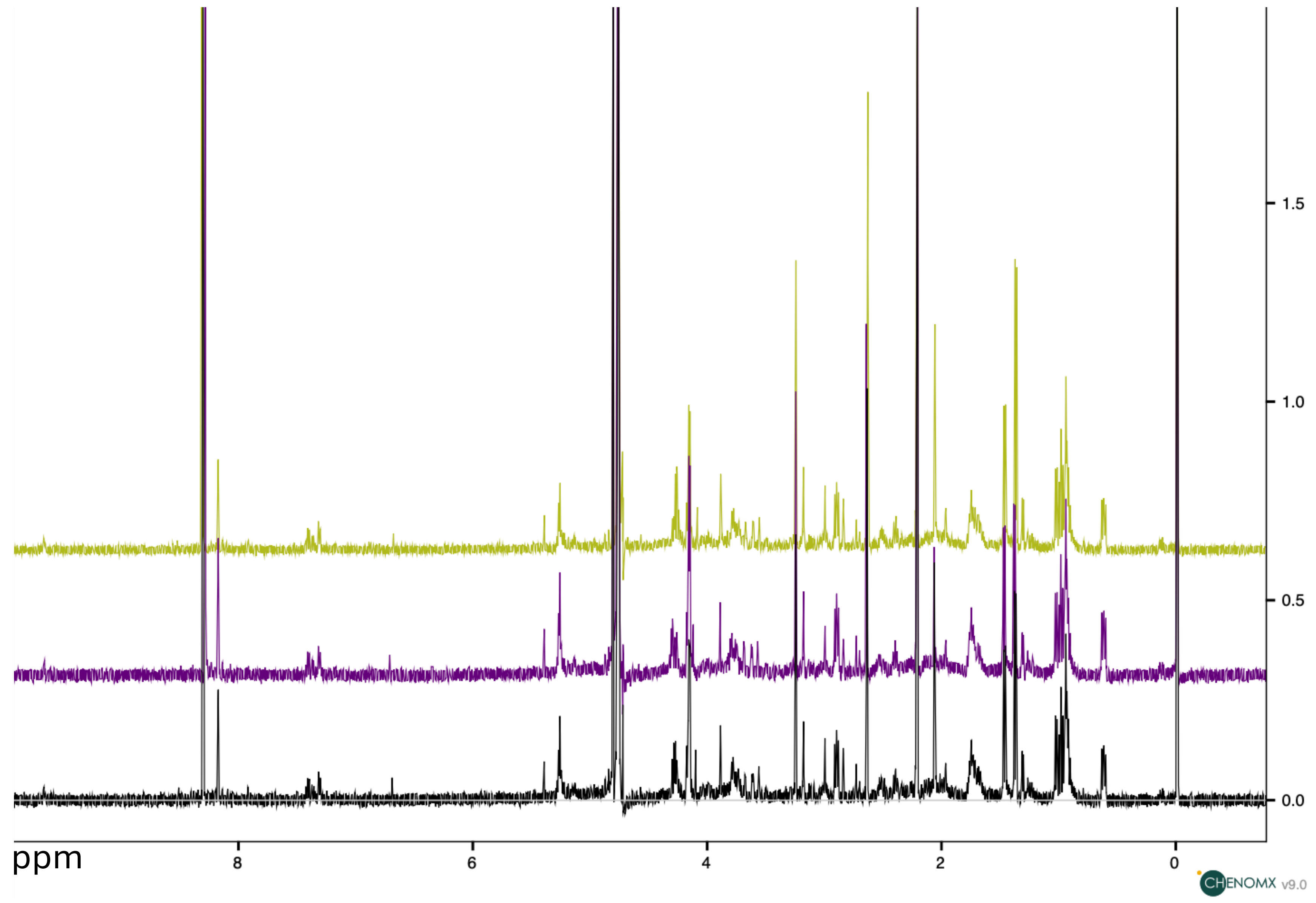
ppm
8
6
4
2
0
1.5
1.0
0.5
0.0
CHENOMX v9.0

i)

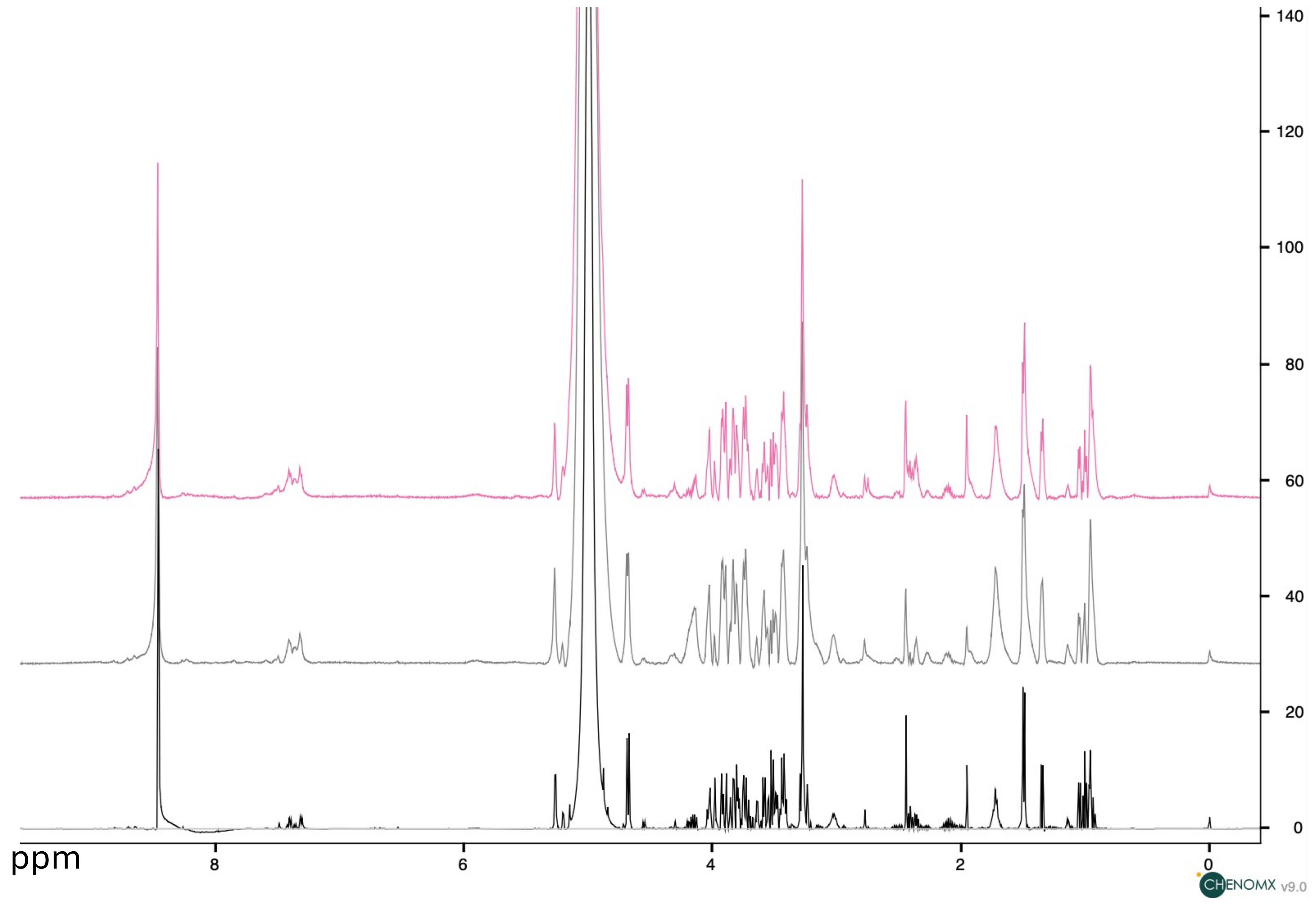
ppm
8
6
4
2
0
140
120
100
80
60
40
20
0
CHENOMX v9.0

j)

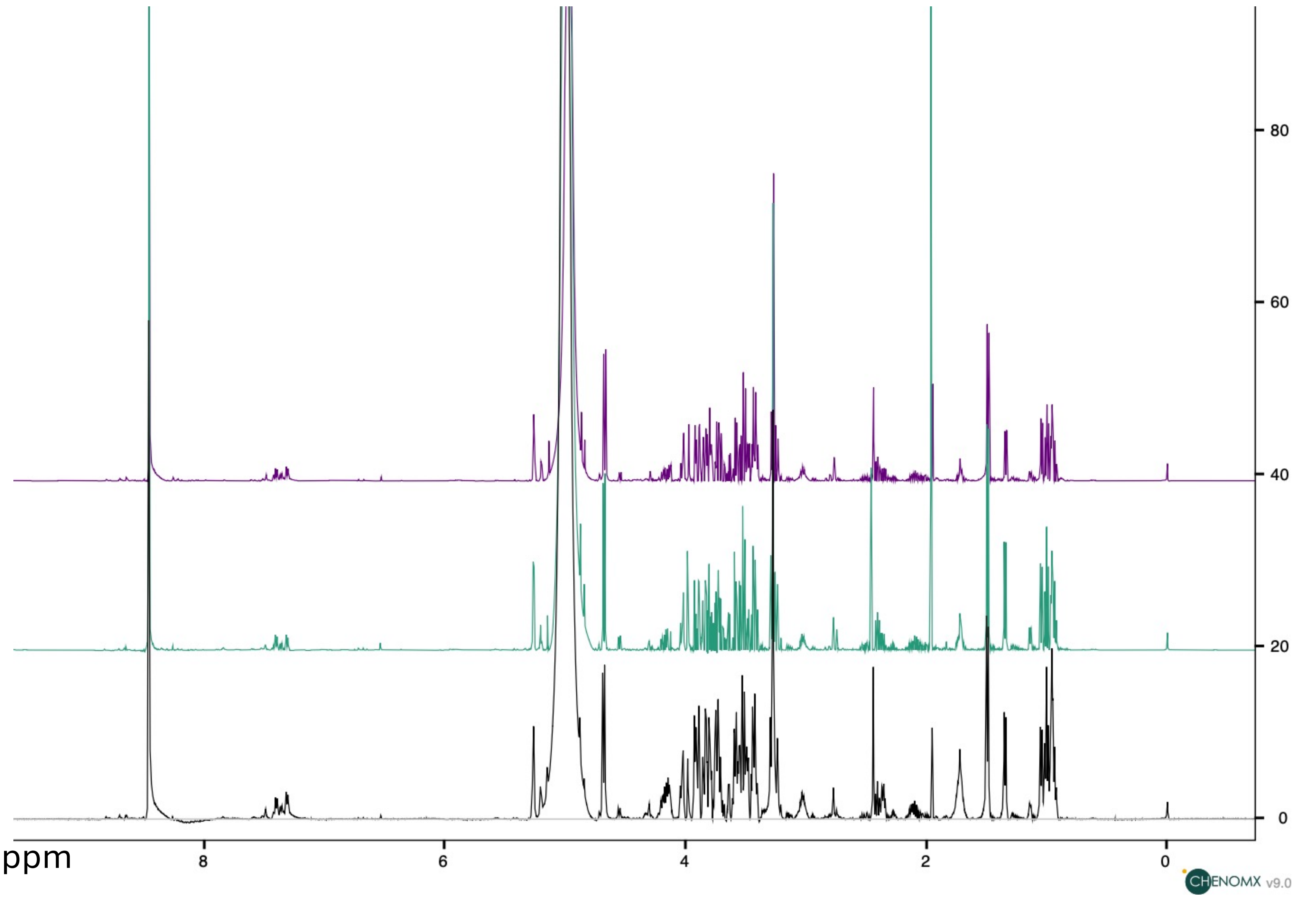

ppm
8
6
4
2
0
80
60
40
20
0
CHENOMX v9.0

k)

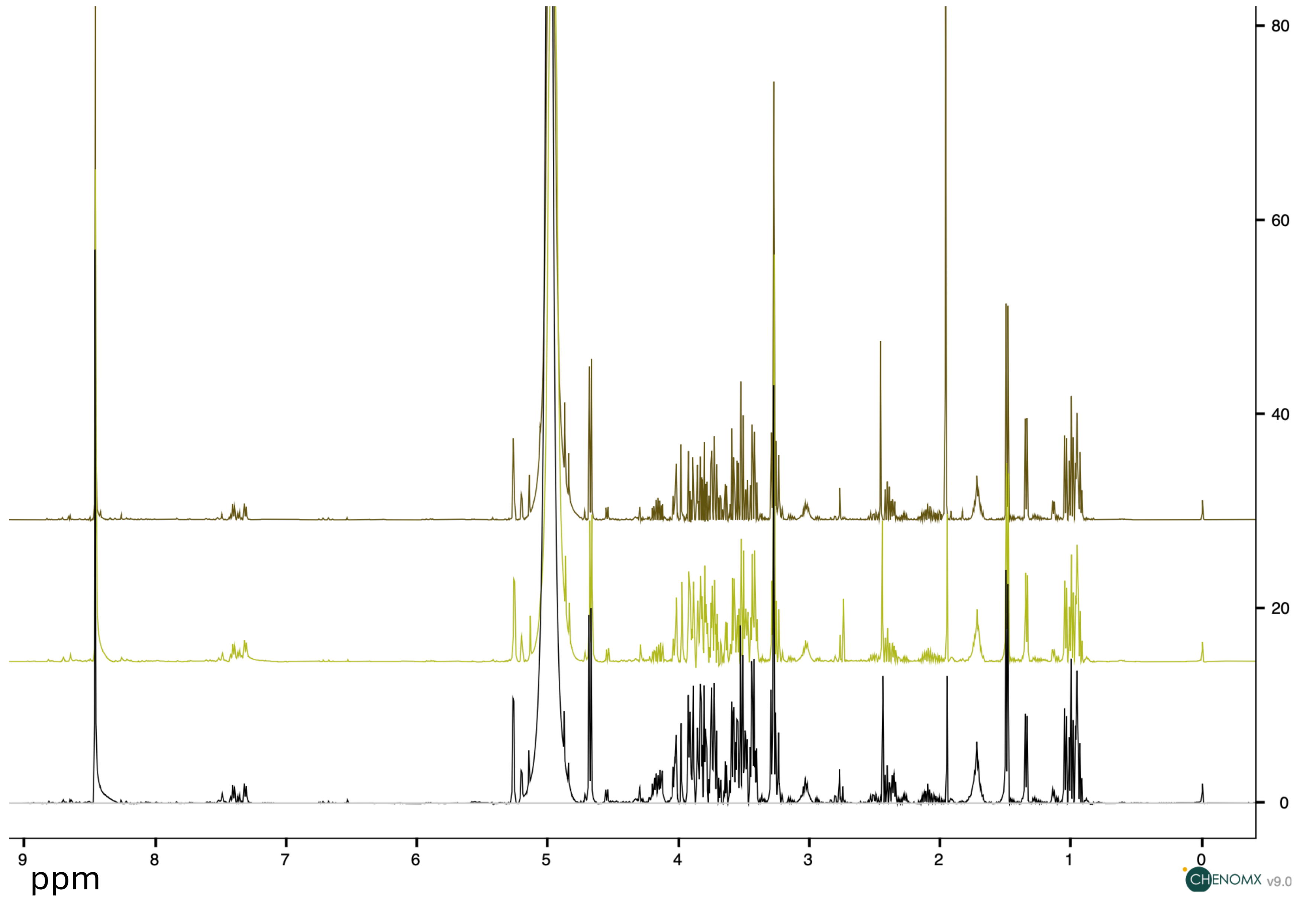

ppm
9
8
7
6
5
4
3
2
1
0
80
60
40
20
0
CHENOMX v9.0

l)

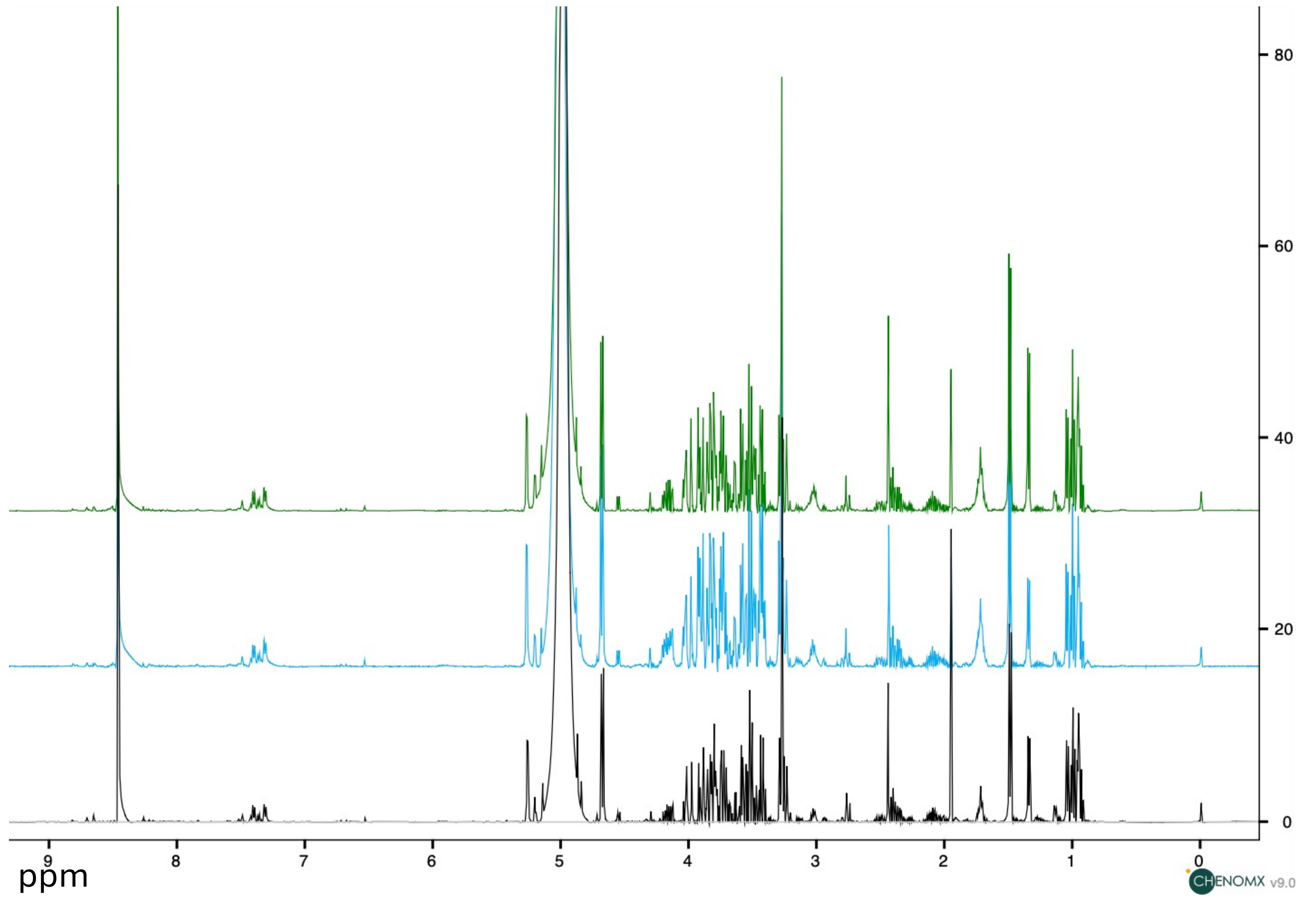

ppm
9
8
7
6
5
4
3
2
1
0
80
60
40
20
0
CHENOMX v9.0

m)

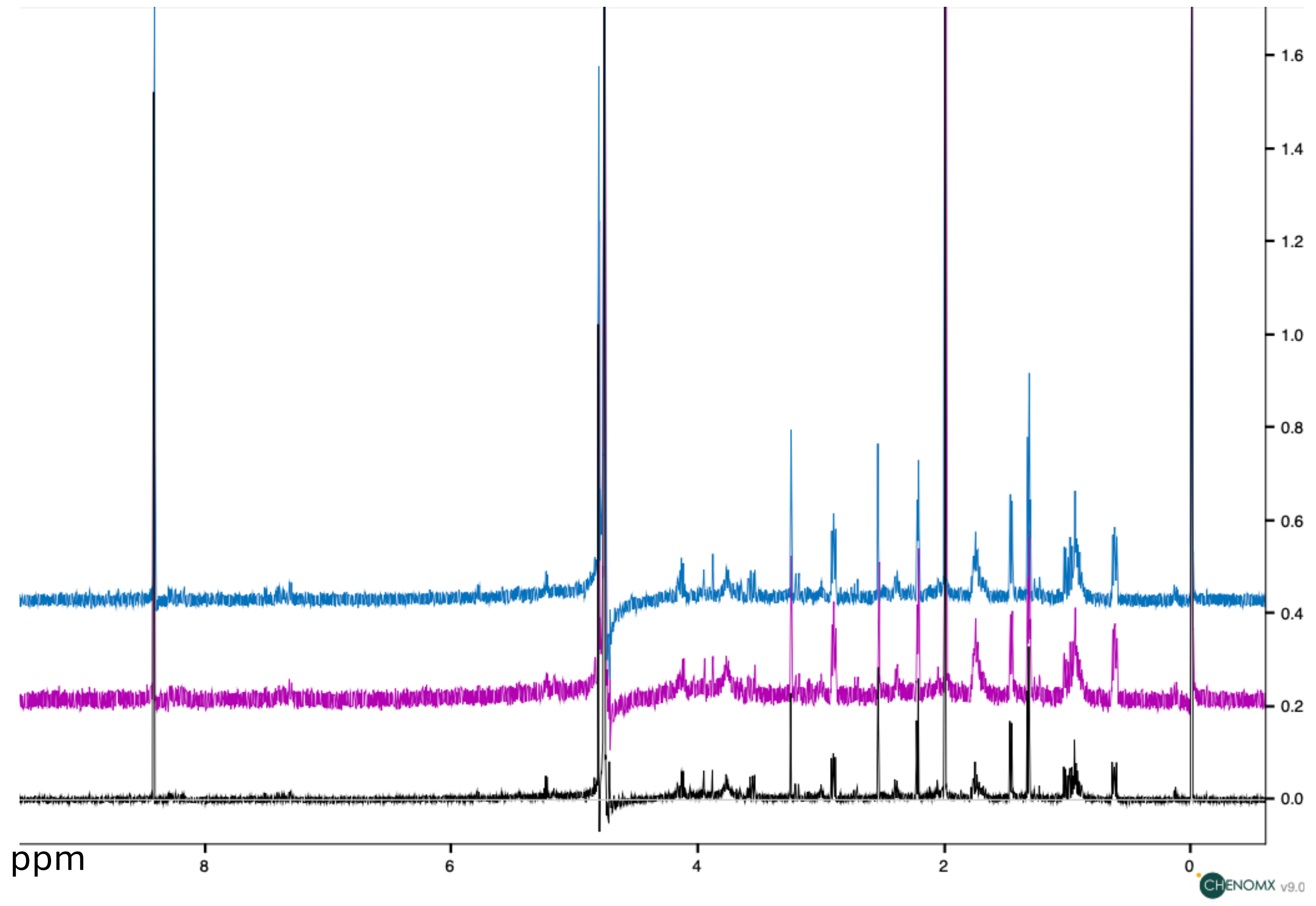
1.6
1.4
1.2
1.0
0.8
0.6
0.4
0.2
0.0
ppm
8
6
4
2
0
CHENOMX v9.0

n)

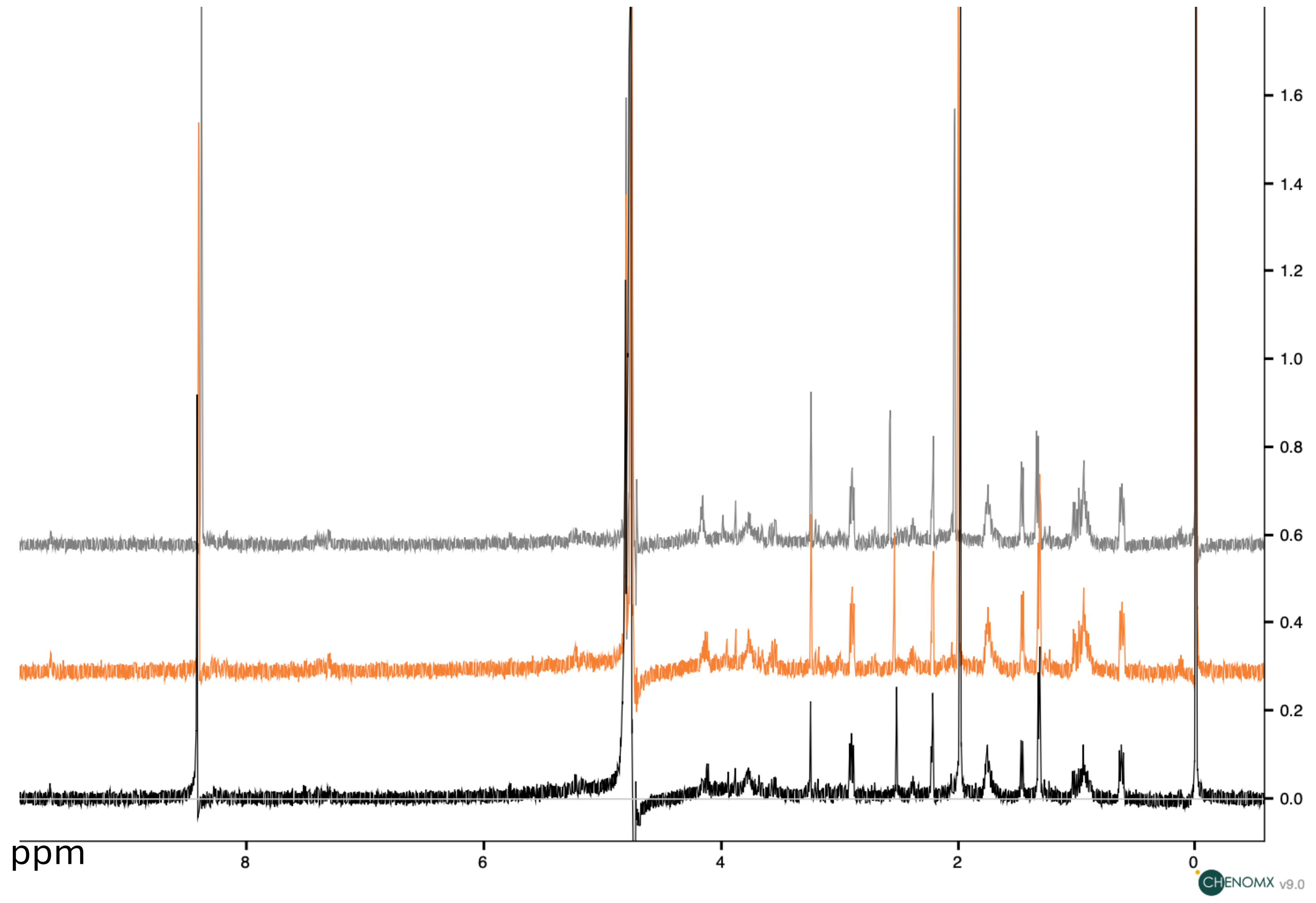
1.6
1.4
1.2
1.0
0.8
0.6
0.4
0.2
0.0
ppm
8
6
4
2
0
CHENOMX v9.0

o)

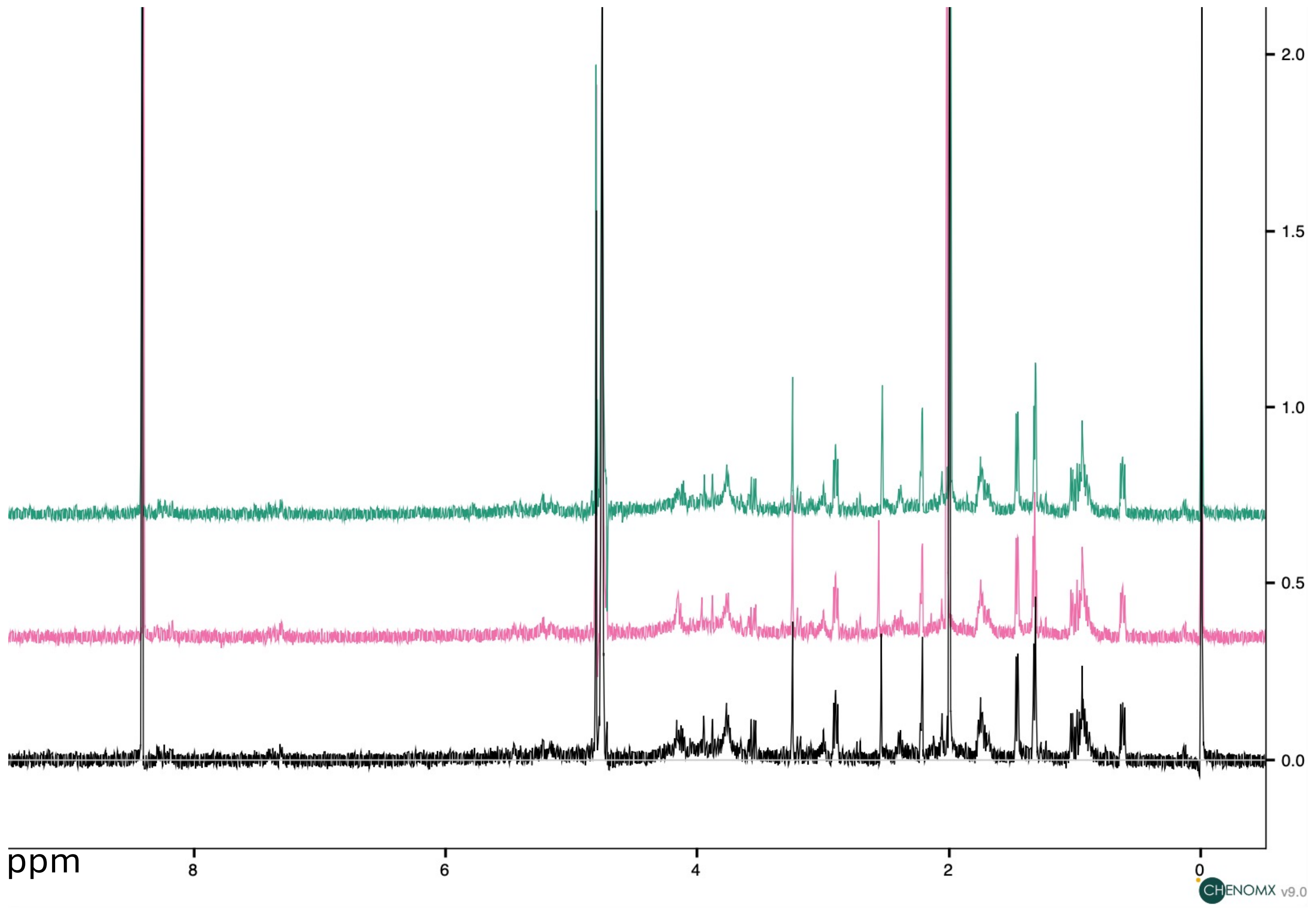
2.0
1.5
1.0
0.5
0.0
ppm
8
6
4
2
0
CHENOMX v9.0

p)

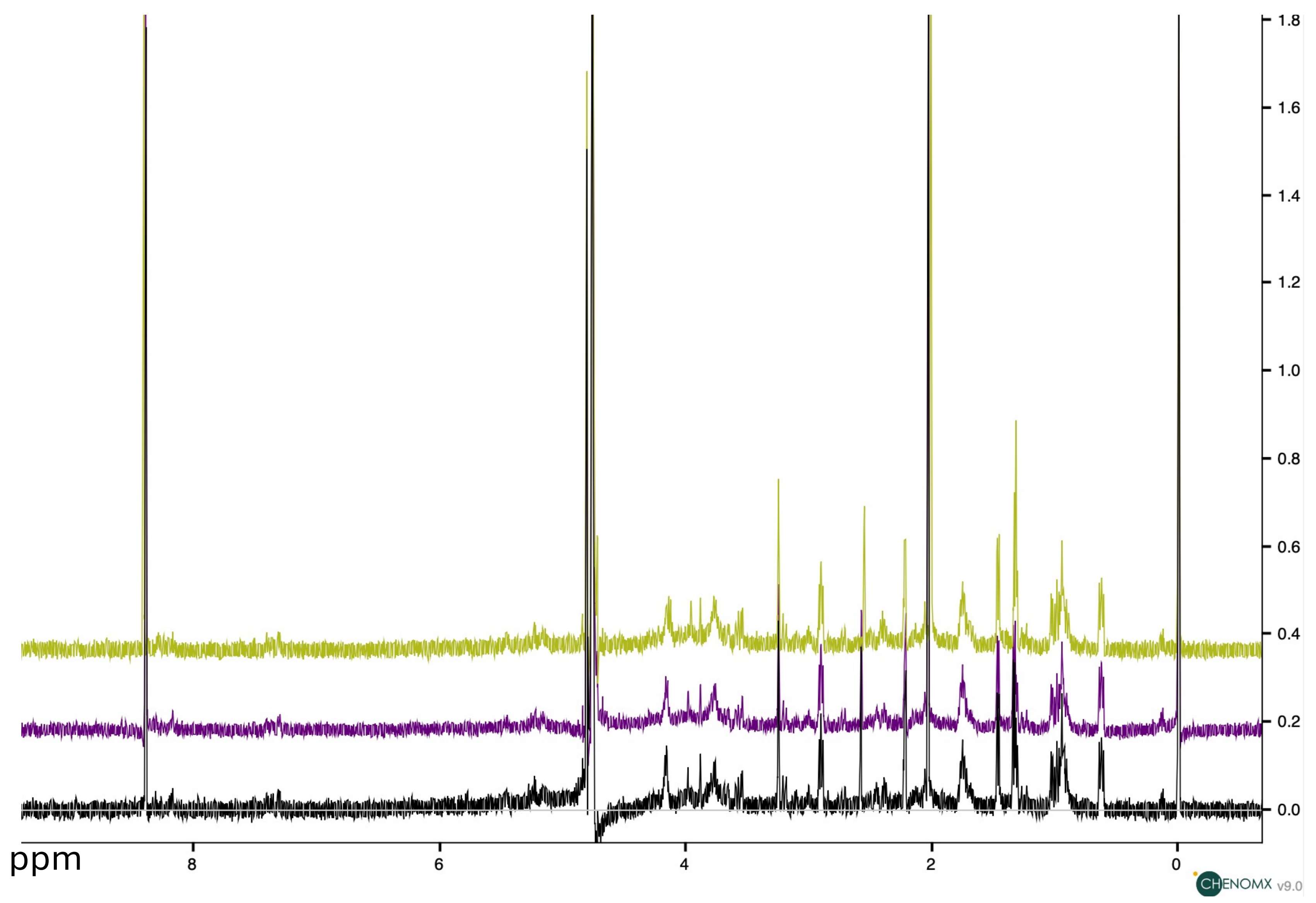
1.8
1.6
1.4
1.2
1.0
0.8
0.6
0.4
0.2
0.0
ppm
8
6
4
2
0
CHENOMX v9.0

q)

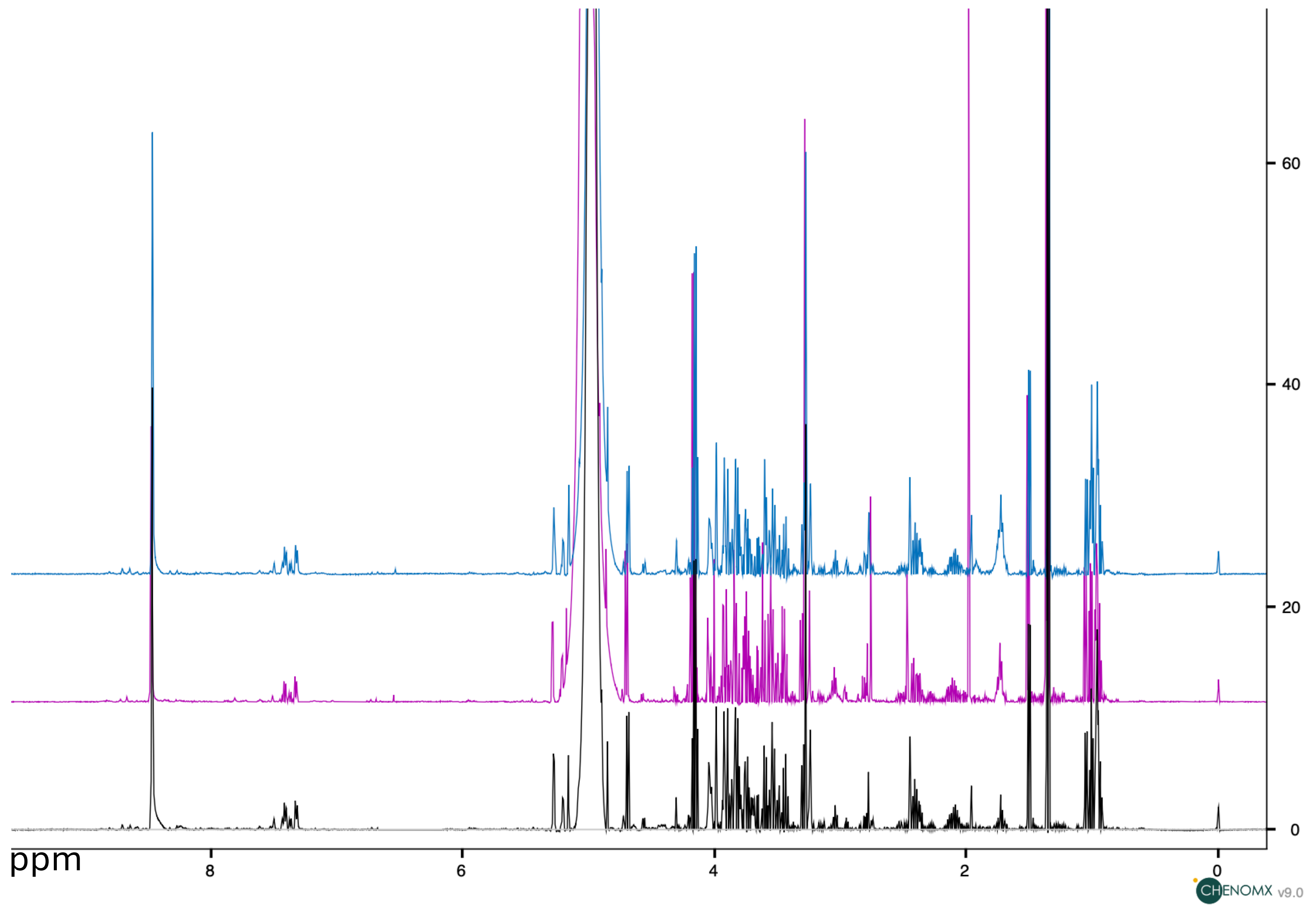
60
40
20
0
ppm
8
6
4
2
0
CHENOMX v9.0

r)

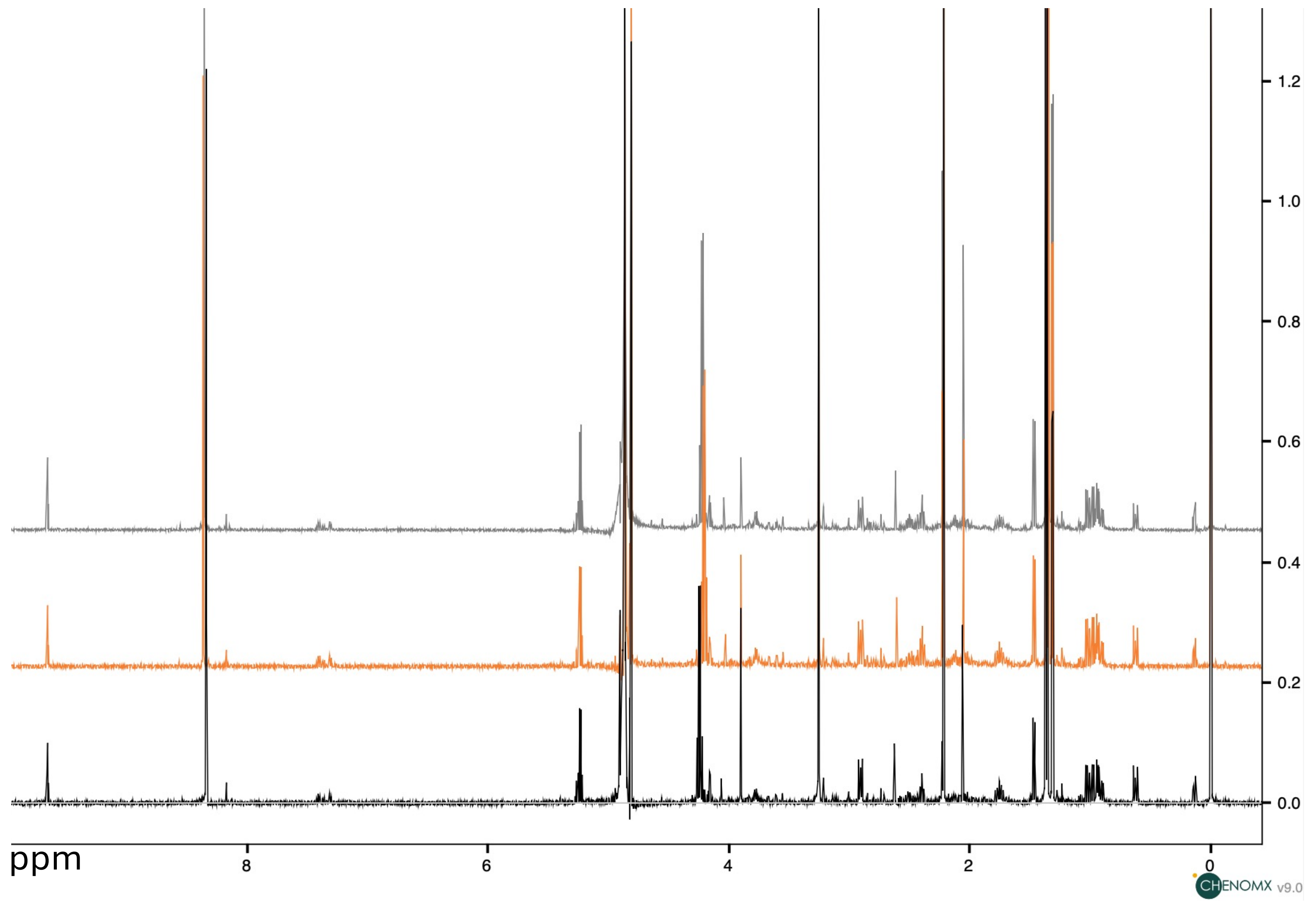
1.2
1.0
0.8
0.6
0.4
0.2
0.0
ppm
8
6
4
2
0
CHENOMX v9.0

s)

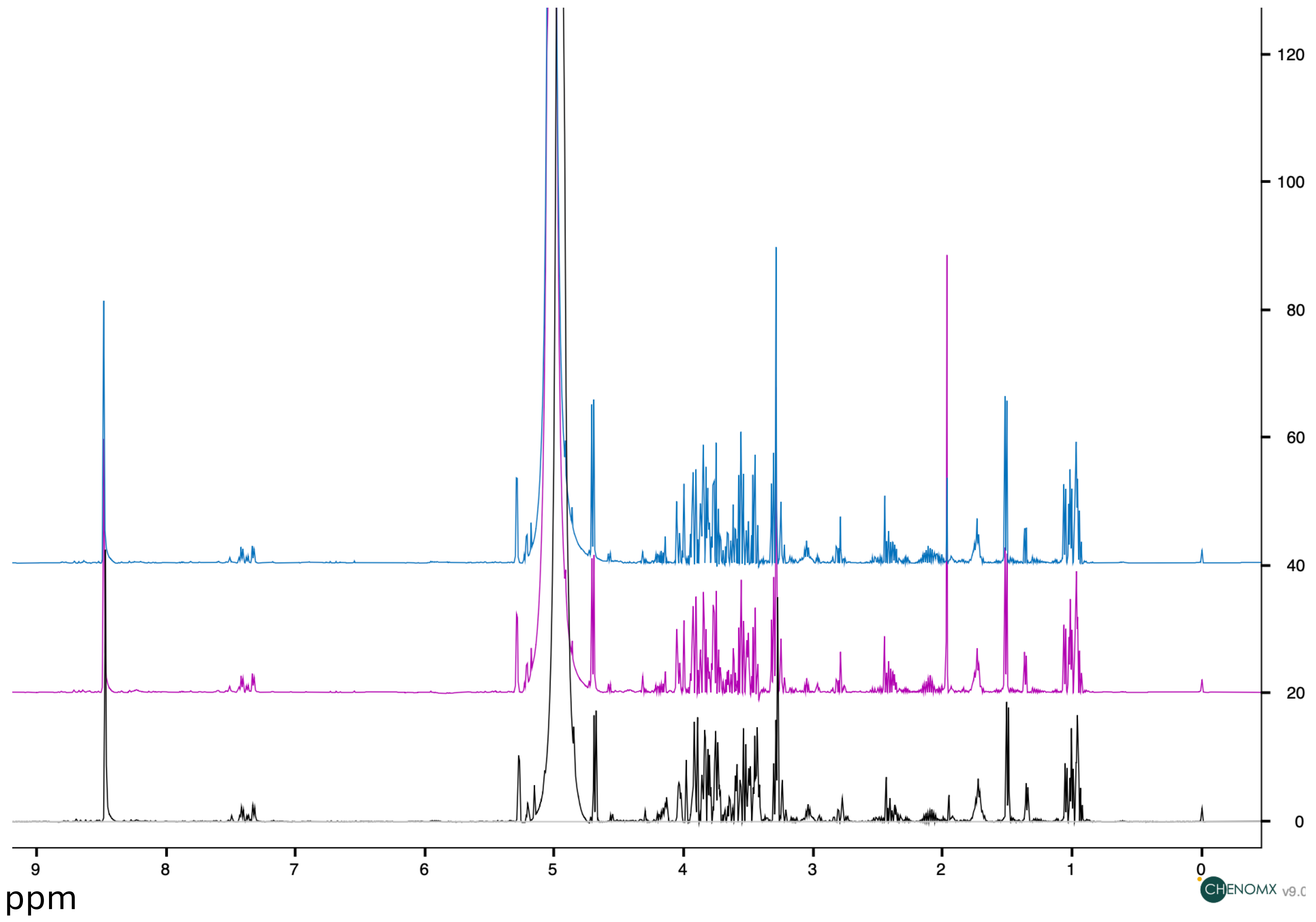
120
100
80
60
40
20
0
9
8
7
6
5
4
3
2
1
0
ppm
CHENOMX v9.0

t)

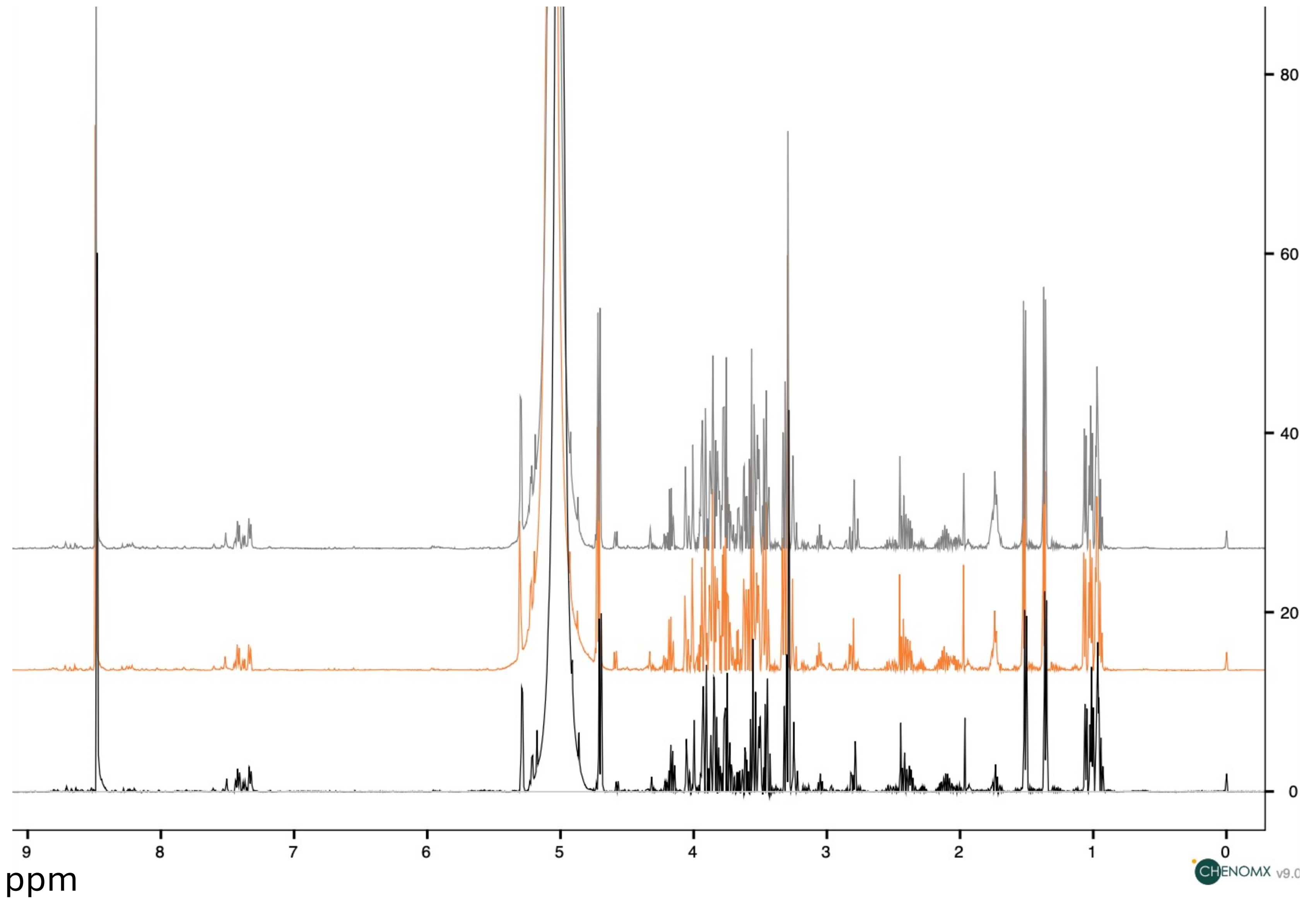
80
60
40
20
0
9
8
7
6
5
4
3
2
1
0
ppm
CHENOMX v9.0

u)

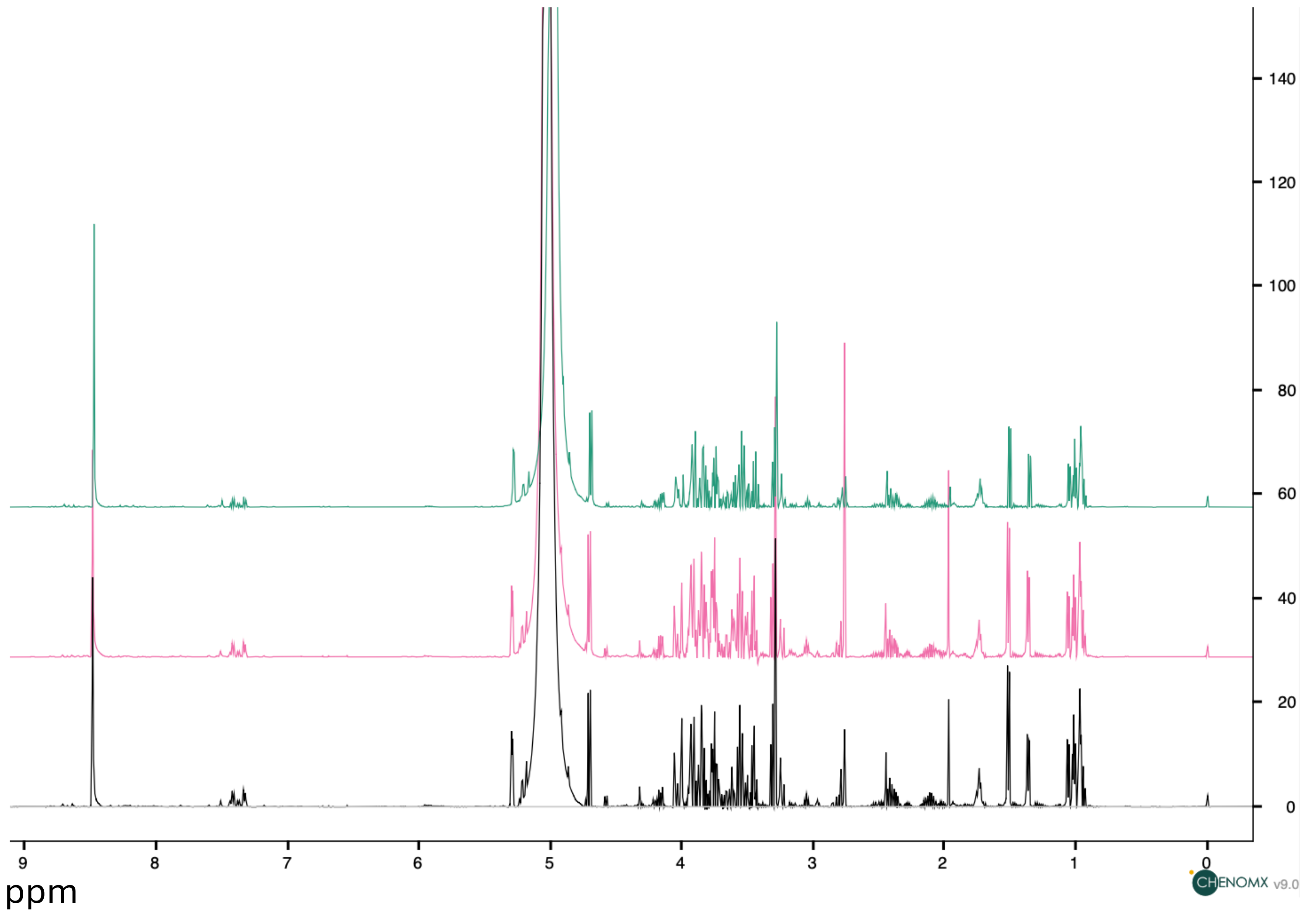
140
120
100
80
60
40
20
0
9
8
7
6
5
4
3
2
1
0
ppm
CHENOMX v9.0

v)

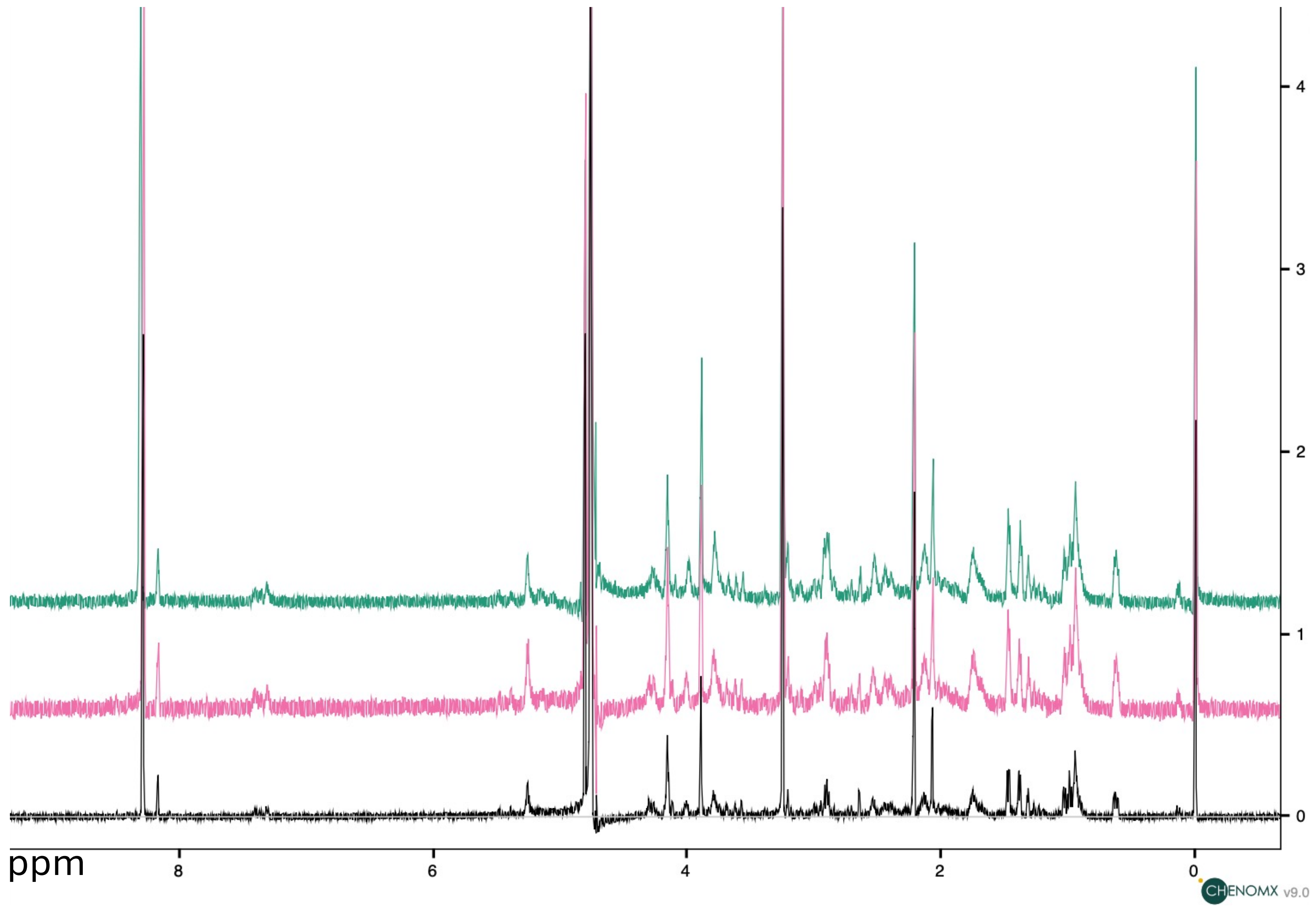

ppm
8 6 4 2 0
0 1 2 3 4
CHENOMX v9.0

w)

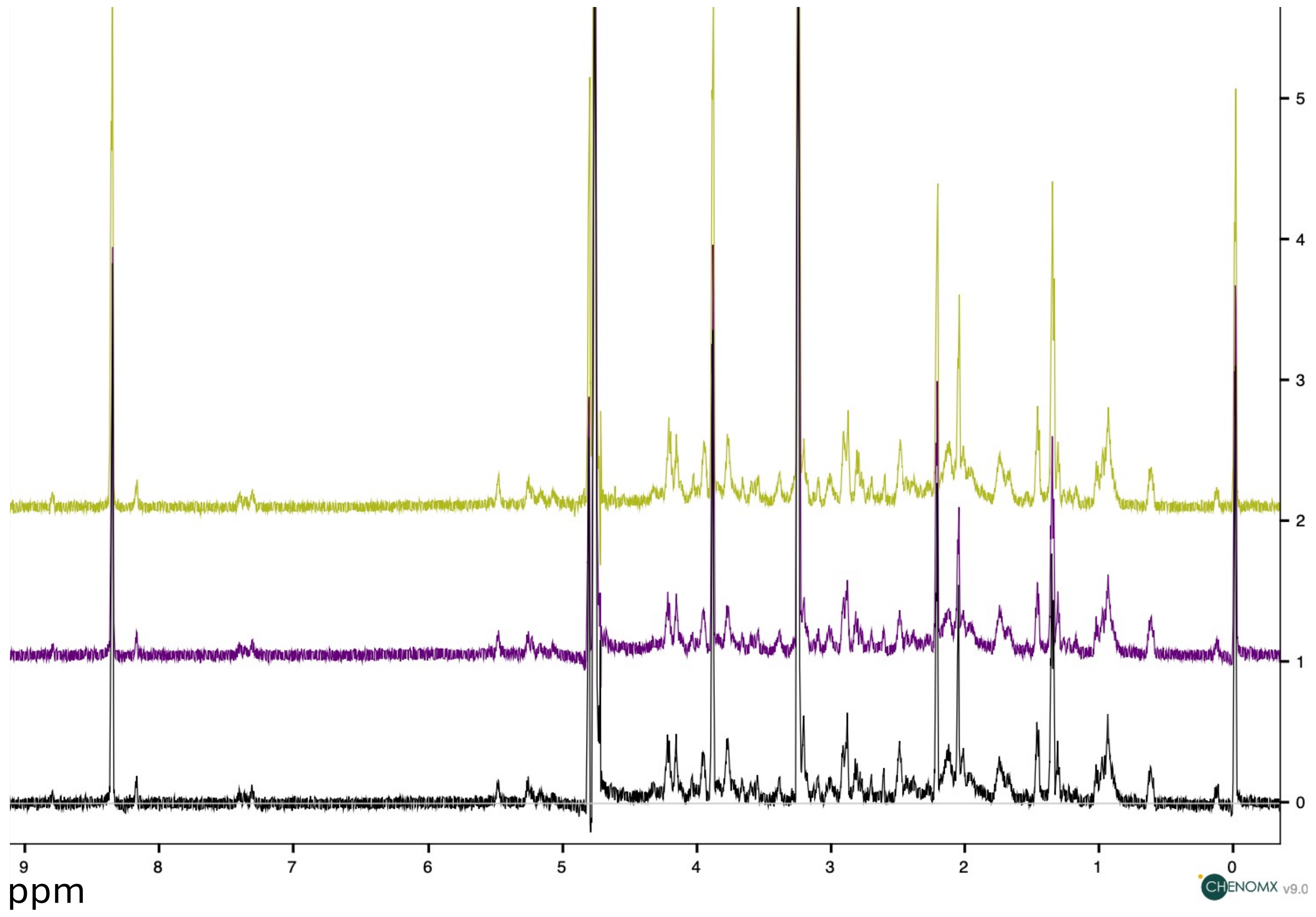

ppm
9 8 7 6 5 4 3 2 1 0
0 1 2 3 4 5
CHENOMX v9.0

x)

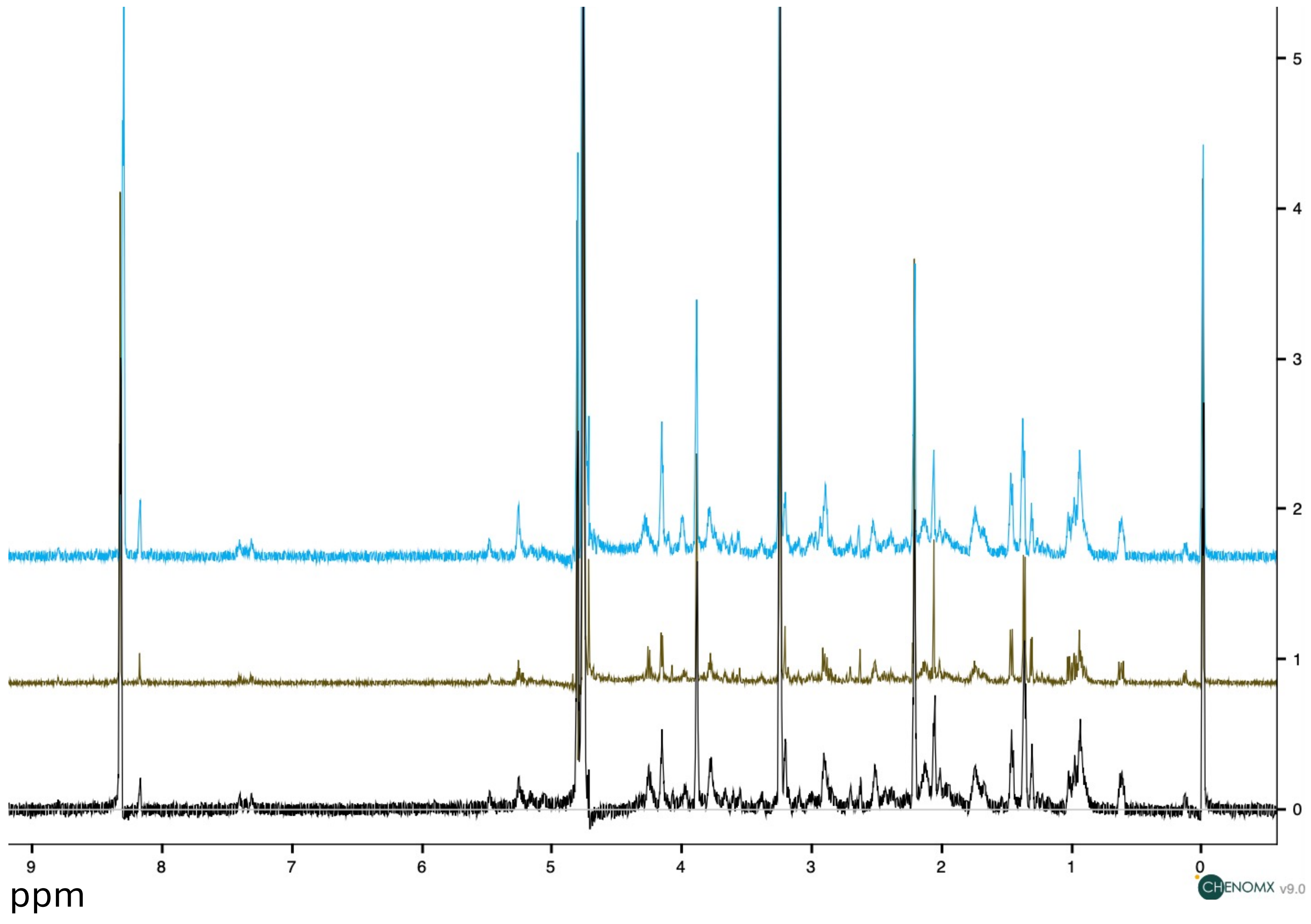

ppm
9 8 7 6 5 4 3 2 1 0
0 1 2 3 4 5
CHENOMX v9.0

Supplementary Figure 8. ^{1}H NMR spectral overlays of three biological replicates for each studied antibiotic treatment group and species. Spectral overlays of: **(a-d)** Extracellular (EC) and **(e-h)** intracellular (IC) metabolites detected in *E. coli* MG1655 treated with **(a,e)** cefotaxime, **(b,f)** ciprofloxacin, **(c,g)** imipenem, and **(d,h)** kanamycin; **(i-l)** EC and **(m-p)** IC metabolites detected in *K. pneumoniae* NCTC418 treated with **(i,m)** cefotaxime, **(j,n)** ciprofloxacin, **(k,o)** imipenem, and **(l,p)** kanamycin; **(q)** EC and **(r)** IC metabolites detected in *E. faecium* NCTC13169 treated with chloramphenicol; **(s-u)** EC and **(v-x)** IC metabolites detected in *S. aureus* NCTC8325 treated with **(s,v)** chloramphenicol, **(t,w)** oxacillin, and **(u,x)** vancomycin.

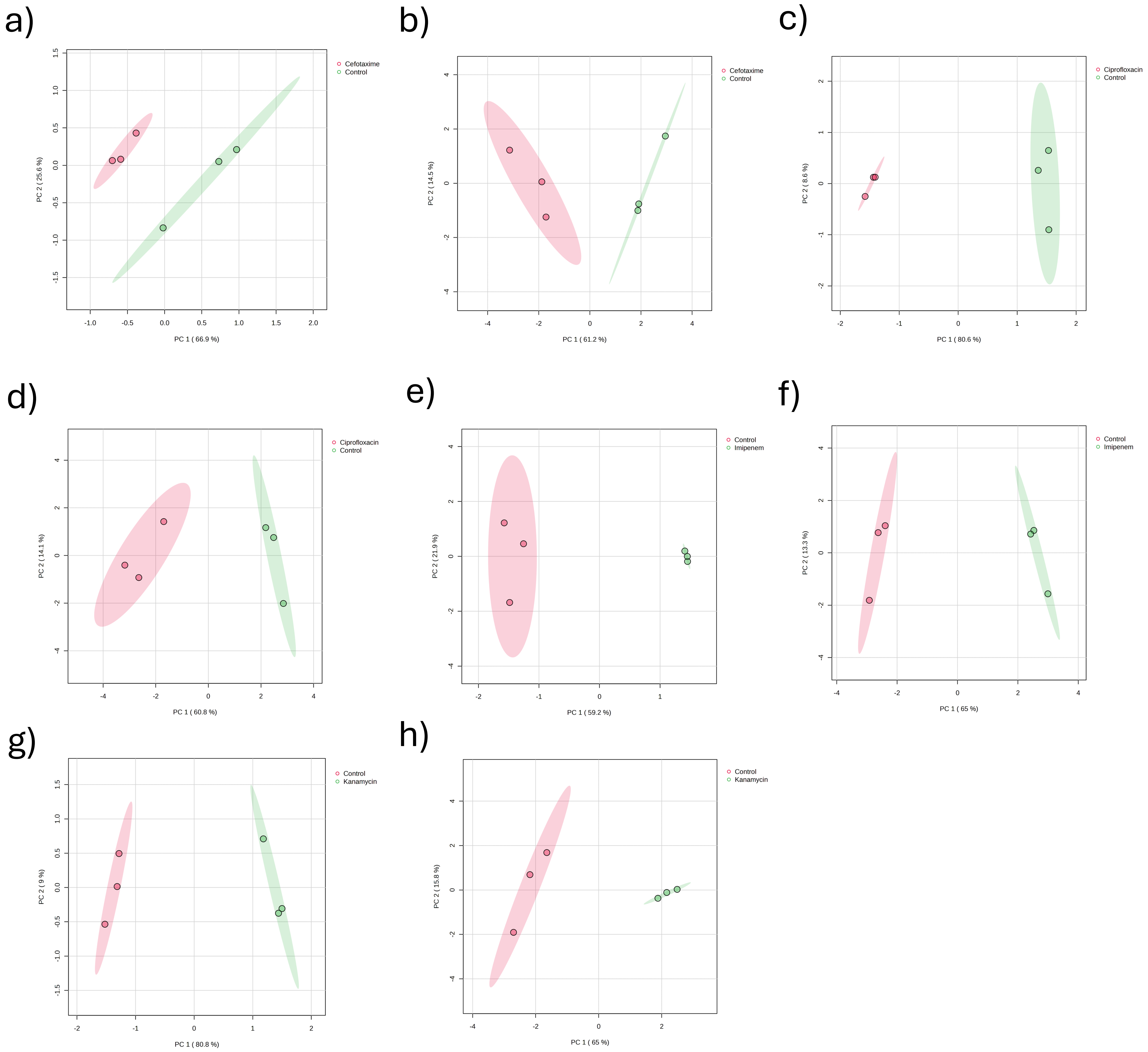

Supplementary Figure 9. Principal component analysis (PCA) score plots of normalised metabolite concentrations in *E. coli* MG1655. Figures present PCA plots of intracellular **(a, c, e, g)** and extracellular **(b, d, f, h)** metabolite concentrations across three biological samples per sub-MIC antibiotic vs. control treatment group: **(a,b)** cefotaxime vs. control; **(c,d)** ciprofloxacin vs. control; **(e, f)** imipenem vs. control; **(g, h)** kanamycin vs. control. Shaded areas indicate the 95% confidence region. Colours refer to treatment groups.

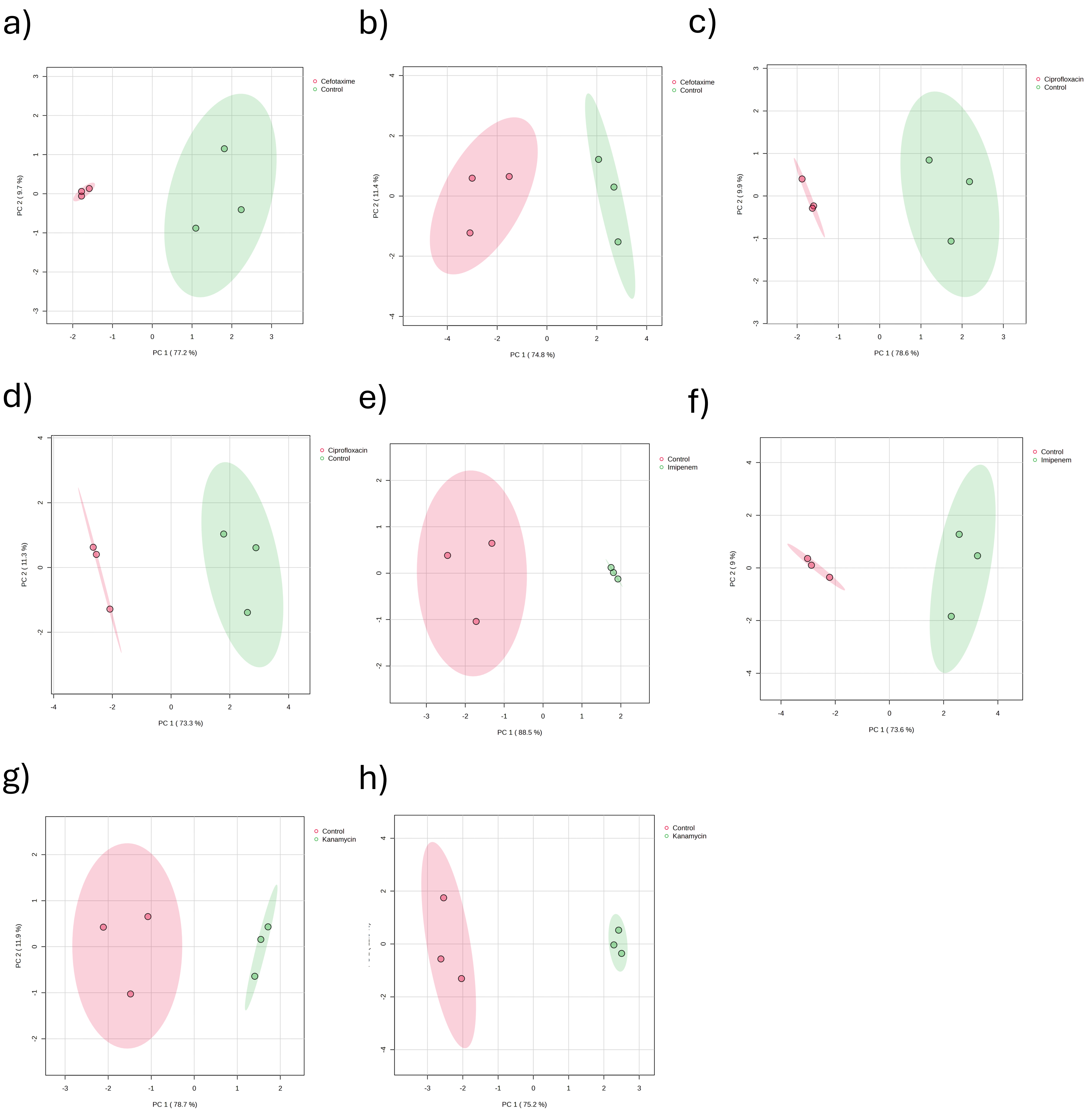

Supplementary Figure 10. Principal component analysis (PCA) score plots of normalised metabolite concentrations in *K. pneumoniae* NCTC418. Figures present PCA plots of intracellular **(a, c, e, g)** and extracellular **(b, d, f, h)** metabolite concentrations across three biological samples per sub-MIC antibiotic vs. control treatment group in : **(a,b)** cefotaxime vs. control; **(c,d)** ciprofloxacin vs. control; **(e, f)** imipenem vs. control; **(g, h)** kanamycin vs. control. Shaded areas indicate the 95% confidence region. Colours refer to treatment groups.

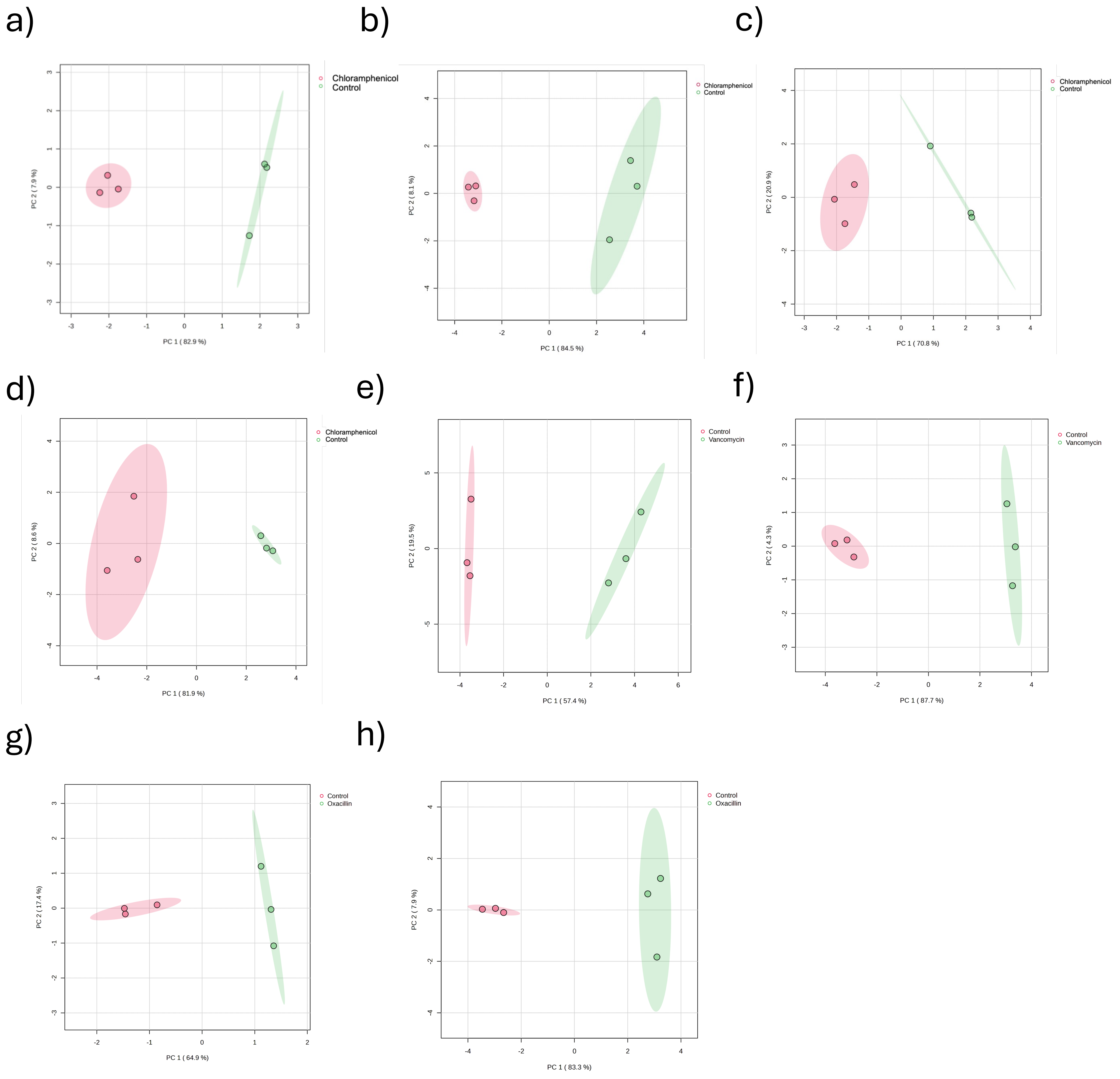

Supplementary Figure 11. Principal component analysis (PCA) score plots of normalised metabolite concentrations in *E. faecium* NCTC13169 and *S. aureus* NCTC8325. Figures present PCA plots of intracellular **(a, c, e, g)** and extracellular **(b, d, f, h)** metabolite concentrations across three biological samples per sub-MIC antibiotic vs. control treatment group in **(a-b)** *E. faecium* NCTC13169 and **(c-h)** *S. aureus* NCTC8325: **(a-d)** chloramphenicol vs. control; **(e, f)** vancomycin vs. control; **(g-h)** oxacillin vs. control. Shaded areas indicate the 95% confidence region. Colours refer to treatment groups.

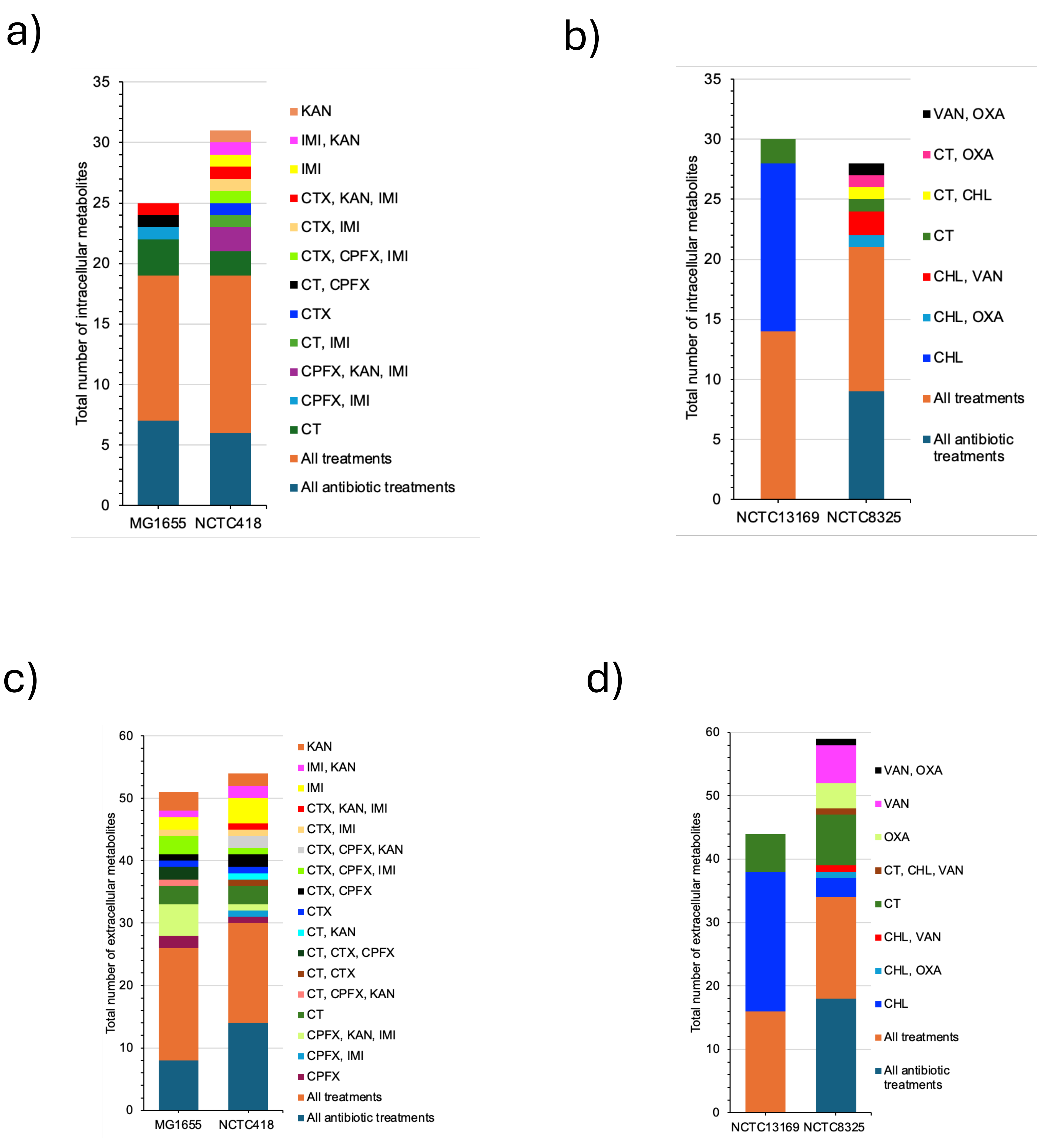

Supplementary Figure 12. Total number of metabolites detected during different treatment conditions or specific to a condition. Figures **(a, b)** present total number of intracellular and **(c, d)** extracellular metabolites detected in Gram(-) **(a,c)** *E. coli* MG1655 and *K. pneumoniae* NCTC418 and in Gram(+) **(b, d)** *E. faecium* NCTC13169 and *S. aureus* NCTC8325 treated with sub-MIC doses of different classes of antibiotics. Abbreviations: KAN= kanamycin; CT= control; IMI= imipenem; CTX= cefotaxime; CPFX= ciprofloxacin, CHL= chloramphenicol; VAN= vancomycin; OXA= oxacillin.

Supplementary data legend

Supplementary data 1 Parameters used for normalisation, transformation and scaling of metabolomic datasets prior to the differential abundance analysis in MetaboAnalyst 6.0.

Supplementary data 2 Summary statistics generated by amica proteomic analysis tool containing MaxQuant(MQ)-generated summarised 'razor+unique' counts, 'razor+unique' counts per sample, MS/MS ('spectra') counts, potential contaminants, raw and LFQ intensities, DEqMS-generated statistics, and imputed intensities. All proteins passing filter by valid values, MS/MS count and 'razor+unique' count thresholds that have been quantified are set to "+" in the 'Quantified' column. 'NA' refers to non-available values. Additional columns filtering proteins that are significantly 'Increased in abundance' and 'Decreased in abundance' and that passed the defined threshold were added in the table and those were marked with '+'. 'Protein description' column was added to include protein description and names for the corresponding protein accessions located under 'Majority.protein.ID' column.

Supplementary data 3 Averaged concentrations (mM) of intracellular (IC) or extracellular (EC) metabolites detected in at least two biological replicates in at least one treatment condition per species in Gram(-) and Gram(+) bacterial species.

Supplementary data 4 Gene ontology and KEGG pathway enrichment analysis results generated using the DAVID tool

Supplementary data 5 Summary of chloramphenicol-responsive proteins in *E. faecium* NCTC13169 (Data 4.1) and *S. aureus* NCTC8325 (Data 4.2), and vancomycin-responsive proteins in *S. aureus* NCTC8325 (Data 4.3) with protein accessions mapped to a corresponding STRING, KEGG/KO, functional annotation and domain accessions; and a list of genes down-regulated in *lytS* mutant (Pubmed ID:PMC2715716) and in *codY* mutant (Pubmed ID:PMC2876493), which are vancomycin-responsive in *S. aureus* NCTC8325.
